# Author Correction: An autonomous laboratory for the accelerated synthesis of inorganic materials

**DOI:** 10.1038/s41586-025-09992-y

**Published:** 2026-01-19

**Authors:** Nathan J. Szymanski, Bernardus Rendy, Yuxing Fei, Rishi E. Kumar, Tanjin He, David Milsted, Matthew J. McDermott, Max Gallant, Ekin Dogus Cubuk, Amil Merchant, Haegyeom Kim, Anubhav Jain, Christopher J. Bartel, Kristin Persson, Yan Zeng, Gerbrand Ceder

**Affiliations:** 1 Department of Materials Science and Engineering, University of California, Berkeley, Berkeley, CA USA; 2 Materials Sciences Division, Lawrence Berkeley National Laboratory, Berkeley, CA USA; 3 Energy Technologies Area, Lawrence Berkeley National Laboratory, Berkeley, CA USA; 4 Google DeepMind, London, UK

**Keywords:** Design, synthesis and processing, Characterization and analytical techniques, Computational methods

Correction to: *Nature* 10.1038/s41586-023-06734-w Published online 29 November 2023

Following publication of this article, concerns were raised about the unambiguous identification of the compound structures using diffraction as well as the original claims of material novelty. We acknowledge that the original claims of material novelty were subject to misinterpretation—their intention was to indicate that the materials were new to the prediction platform, not necessarily new to science. The article text has been updated to reflect this in the HTML and PDF versions of the article. For a detailed breakdown of the textual changes, please see the annotated PDF article file available as Supplementary Information accompanying this amendment.

In addition, we have manually re-analyzed the diffraction patterns and have confirmed that the prediction platform came to the correct conclusion in 36 of its 40 reported successes, with 4 compounds being inconclusive. This re-analysis was peer-reviewed post-publication. The total number of compounds successfully made by the platform has now been updated to exclude four target materials whose presence is inconclusive from XRD alone. We have also removed one compound from the discussion (Zn2Cr3FeO8) that was mistakenly included in the training data. These changes are reflected in Fig. 2, which has been updated in the HTML and PDF versions of the article, as well as the updated Supplementary Information and Supplementary Data. The edits to the Supplementary Information and Supplementary Data are shown in the PDF files accompanying this amendment.

*Nature* thanks the correspondents who brought these issues to our and the authors’ attention, and the reviewers who assessed the re-analyzed PXRD data post-publication.

## Supplementary information

Annotated, uncorrected article

Edits to Supplementary Information

Edits to Supplementary Data

