# Supplementary figures and images for: An autonomous laboratory for the accelerated synthesis of inorganic materials
Source: Nature. 2023 Nov 29;624(7990):86–91. doi: 10.1038/s41586-023-06734-w (PMC10700133; doi:10.1038/s41586-023-06734-w)

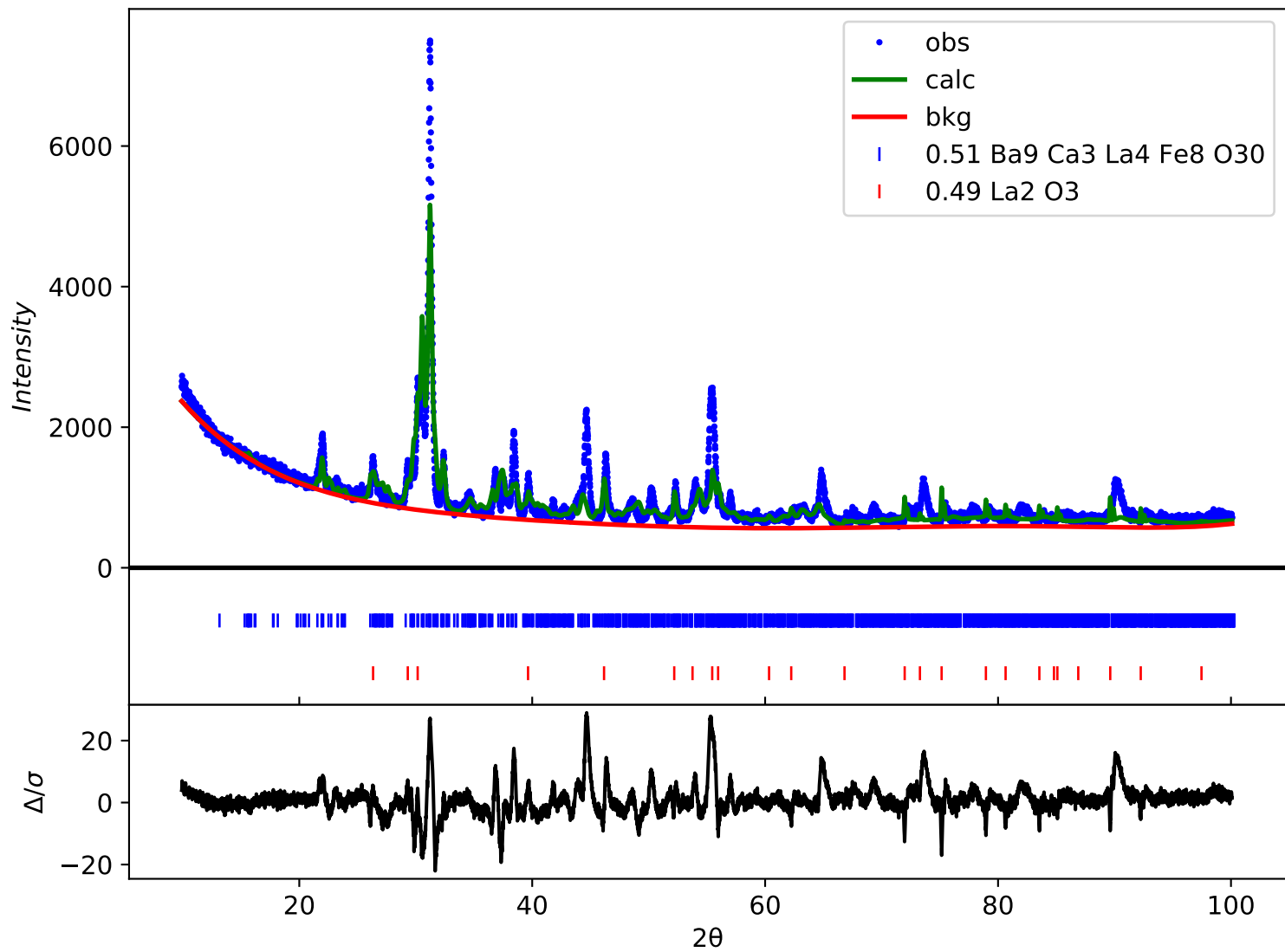

Supplement: Supplementary file 3 — This file contains the refined X-ray diffraction data from the successful syntheses performed by the A-Lab. The corresponding crystal structures used during refinement are also included in CIF format. [file 41586_2023_6734_MOESM3_ESM.zip › Automated_Refinement_Results/Ba9Ca3La4(Fe4O15)2/Ba9Ca3La4(Fe4O15)2_1100_240_BaCO3_CaCO3_Fe2O3_La2O3_recipe171_112bc40c-f92d-4efe-b350-1ae867e6c0ba.pdf]

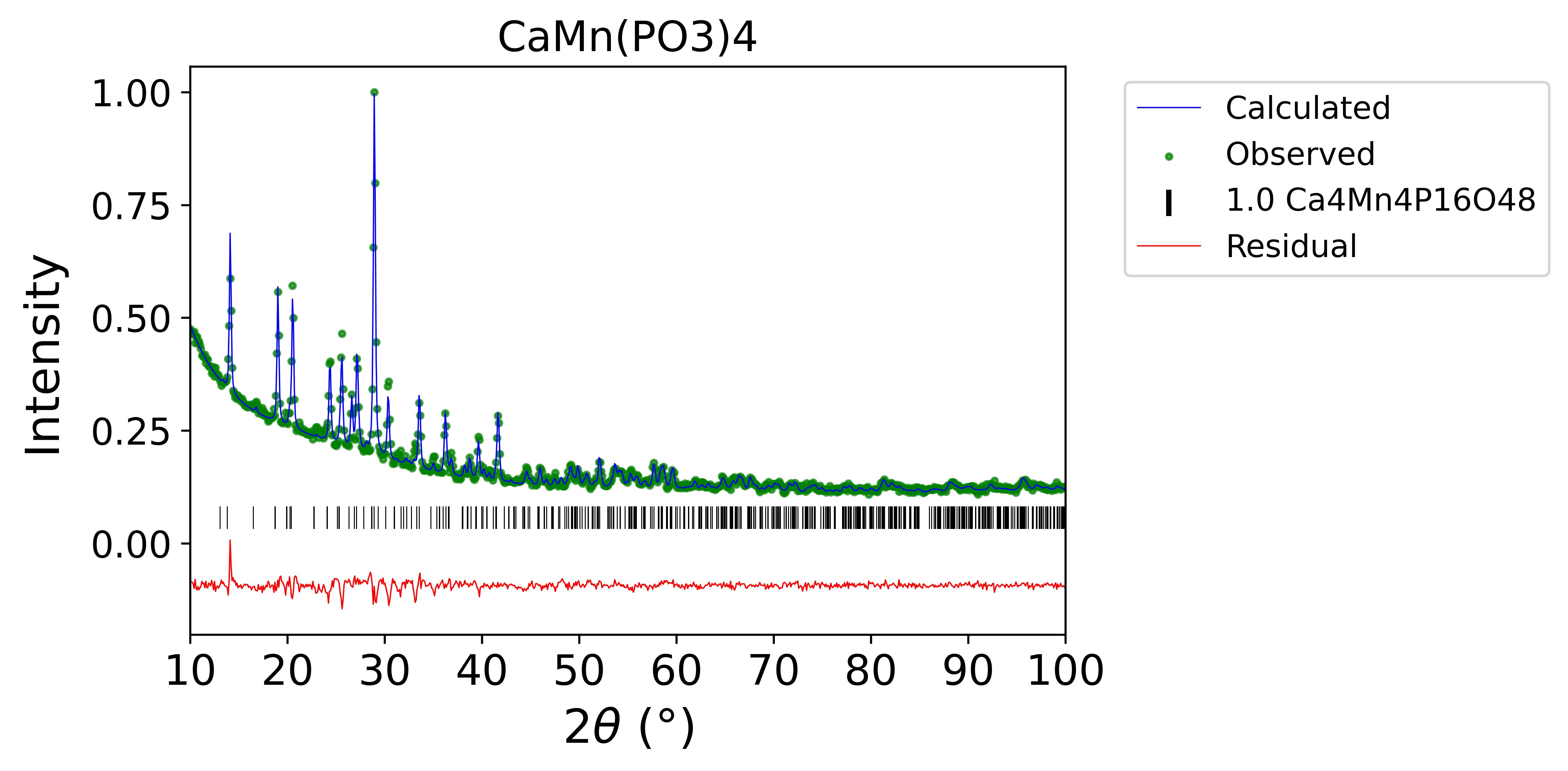

Supplement: Supplementary file 3 — This file contains the refined X-ray diffraction data from the successful syntheses performed by the A-Lab. The corresponding crystal structures used during refinement are also included in CIF format. [file 41586_2023_6734_MOESM3_ESM.zip › Automated_Refinement_Results/CaMn(PO3)4/CaMn(PO3)4_700_240_CaCO3_Mn2O3_(NH4)2HPO4_recipe27_35e3f1a7-cd2f-458b-a733-0f02acc2cdba.jpg]

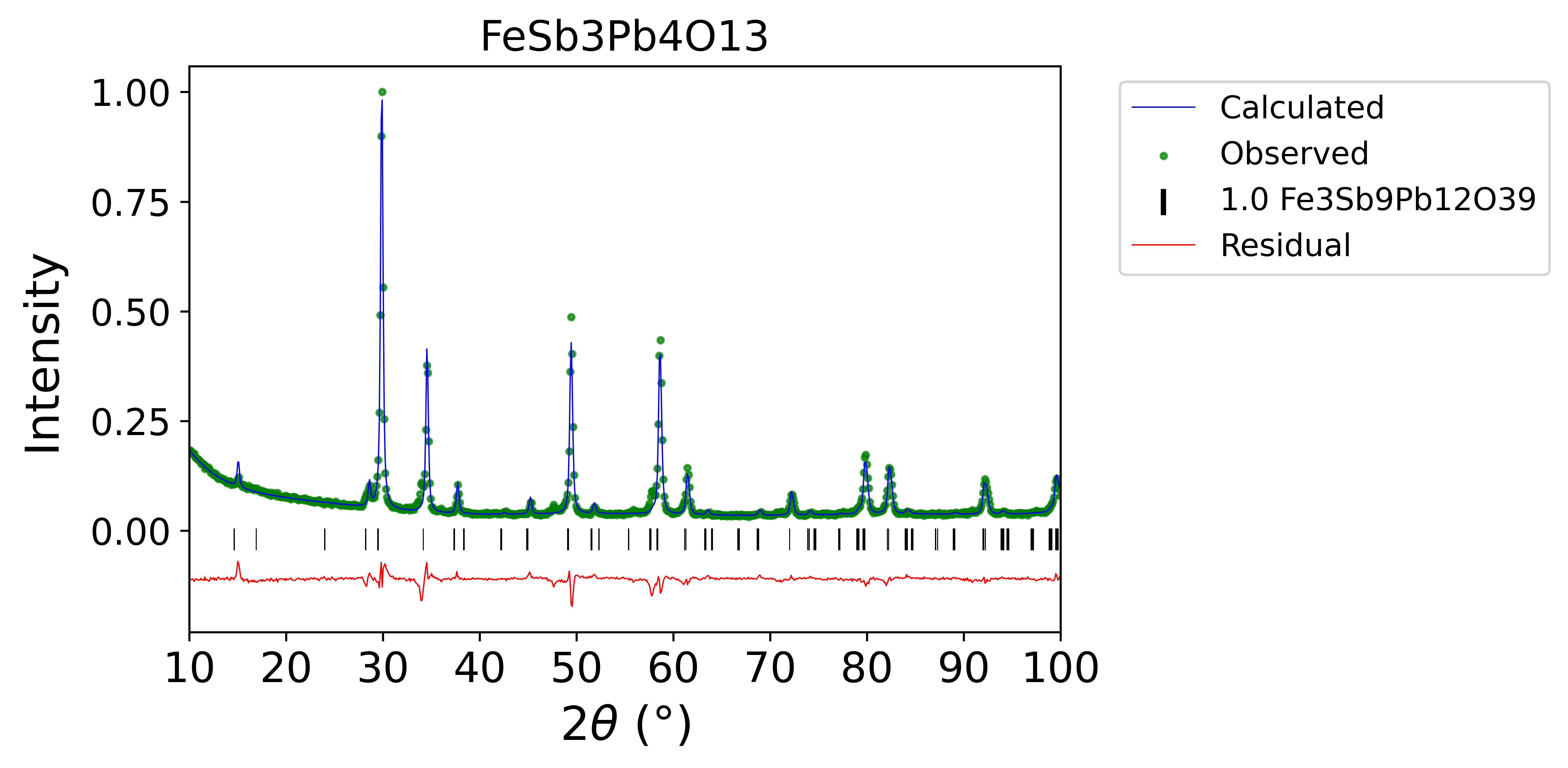

Supplement: Supplementary file 3 — This file contains the refined X-ray diffraction data from the successful syntheses performed by the A-Lab. The corresponding crystal structures used during refinement are also included in CIF format. [file 41586_2023_6734_MOESM3_ESM.zip › Automated_Refinement_Results/FeSb3Pb4O13/FeSb3Pb4O13_800_240_Fe2O3_PbCO3_Sb2O3_recipe216_be70afee-2220-42f8-b4c1-7a1b5f474a19.jpg]

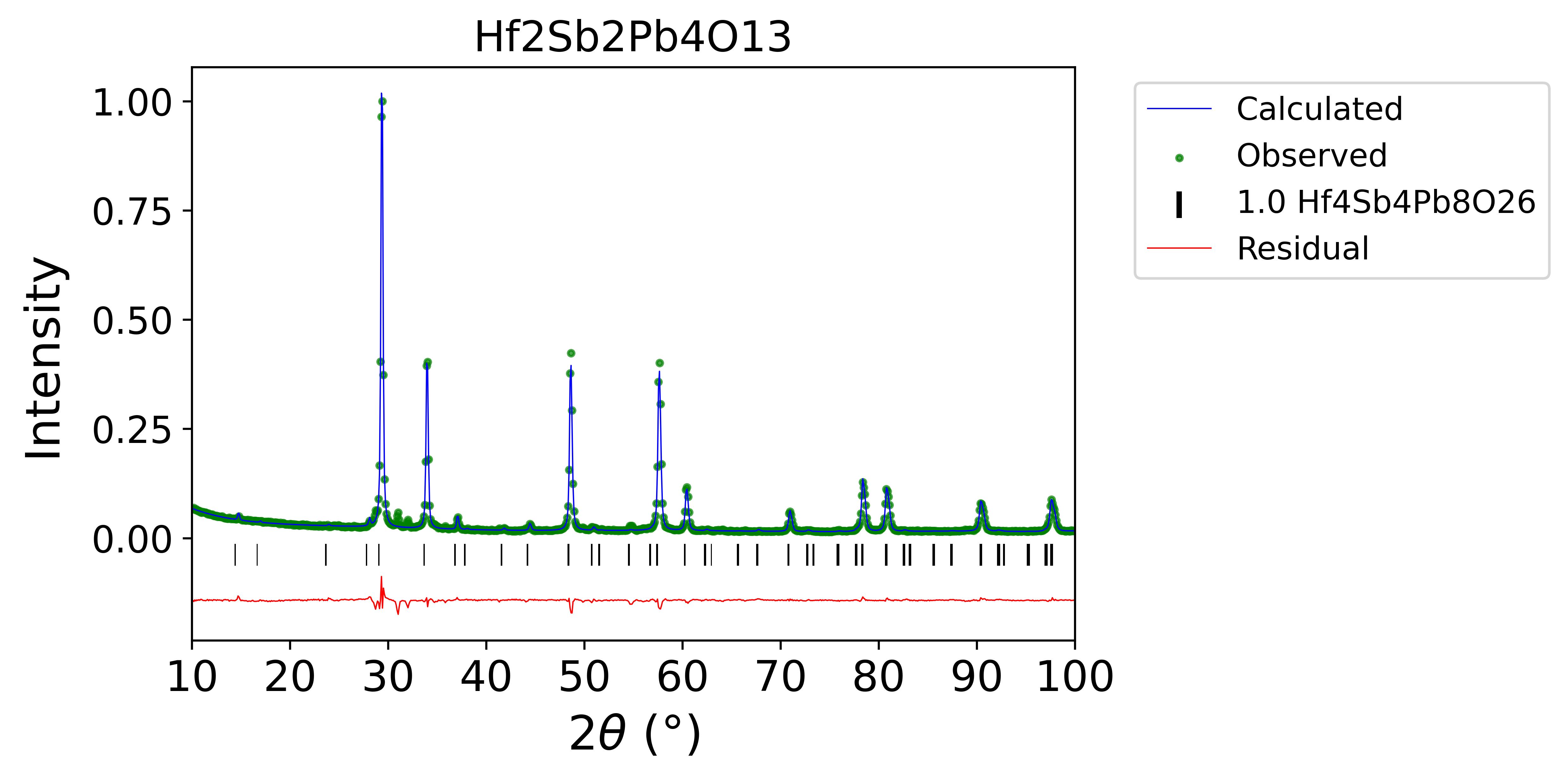

Supplement: Supplementary file 3 — This file contains the refined X-ray diffraction data from the successful syntheses performed by the A-Lab. The corresponding crystal structures used during refinement are also included in CIF format. [file 41586_2023_6734_MOESM3_ESM.zip › Automated_Refinement_Results/Hf2Sb2Pb4O13/Hf2Sb2Pb4O13_900_240_HfO2_PbCO3_Sb2O3_recipe127_709b3ce1-e59c-488f-9448-dd87b4f12ece.jpg]

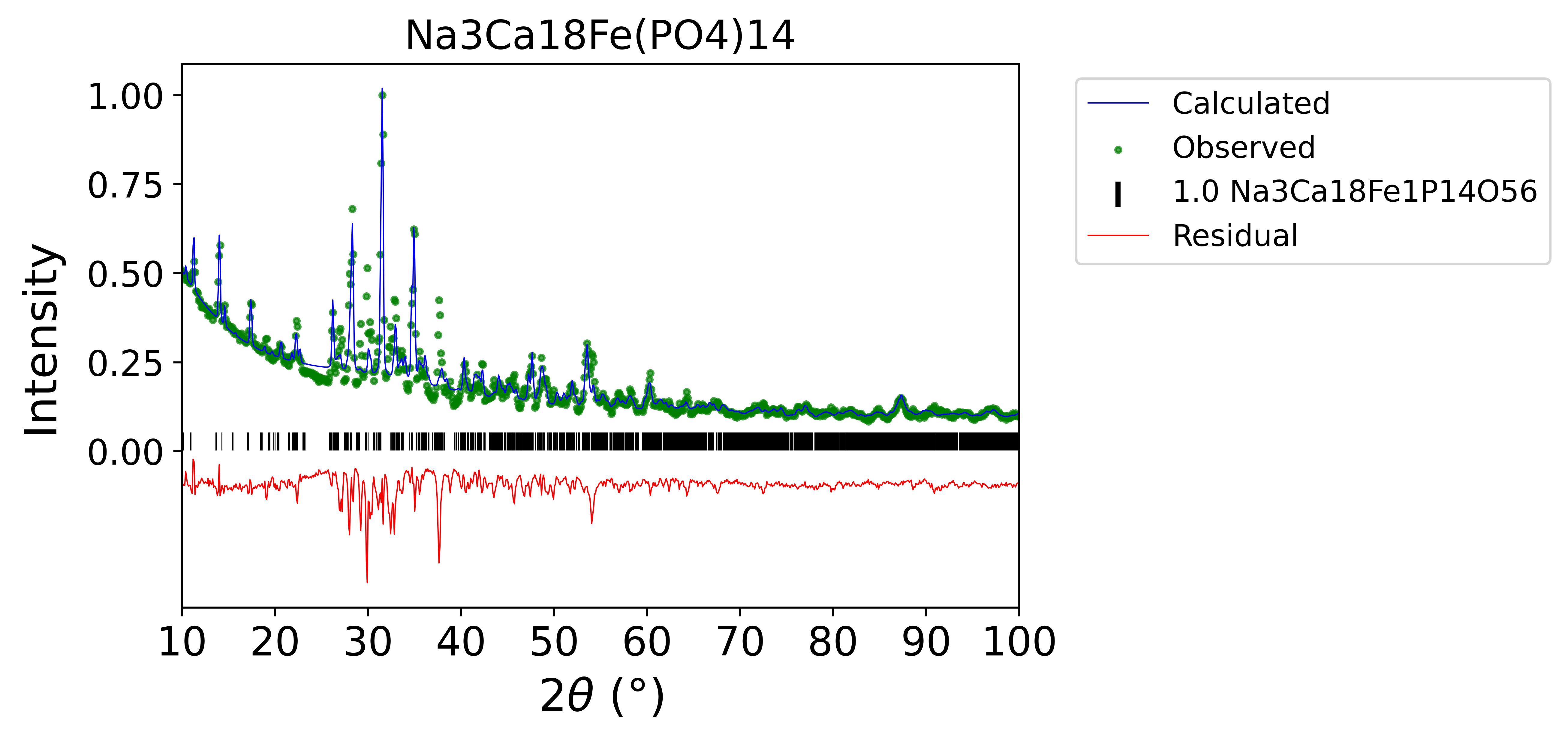

Supplement: Supplementary file 3 — This file contains the refined X-ray diffraction data from the successful syntheses performed by the A-Lab. The corresponding crystal structures used during refinement are also included in CIF format. [file 41586_2023_6734_MOESM3_ESM.zip › Automated_Refinement_Results/Na3Ca18Fe(PO4)14/Na3Ca18Fe(PO4)14_700_240_CaCO3_Fe2O3_NH4H2PO4_Na2CO3_recipe57_5a411c43-0dd1-4a3a-9b20-474aaa8ed3f5.jpg]

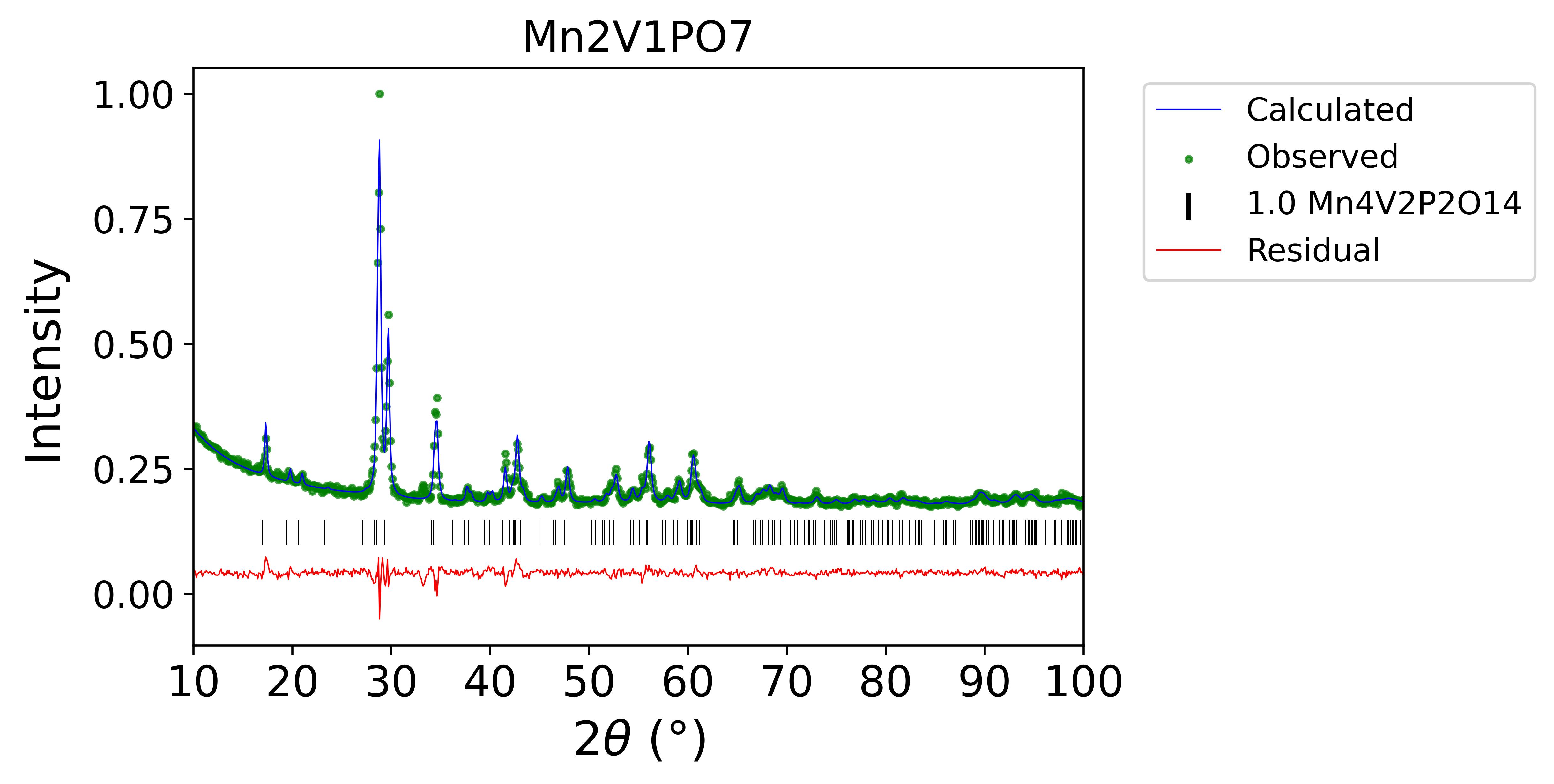

Supplement: Supplementary file 3 — This file contains the refined X-ray diffraction data from the successful syntheses performed by the A-Lab. The corresponding crystal structures used during refinement are also included in CIF format. [file 41586_2023_6734_MOESM3_ESM.zip › Automated_Refinement_Results/Mn2VPO7/Mn2V1PO7_700_240_MnO2_V2O5_NH4H2PO4_recipe77_a612be01-4c76-458e-8800-c9aa5737183e.jpg]

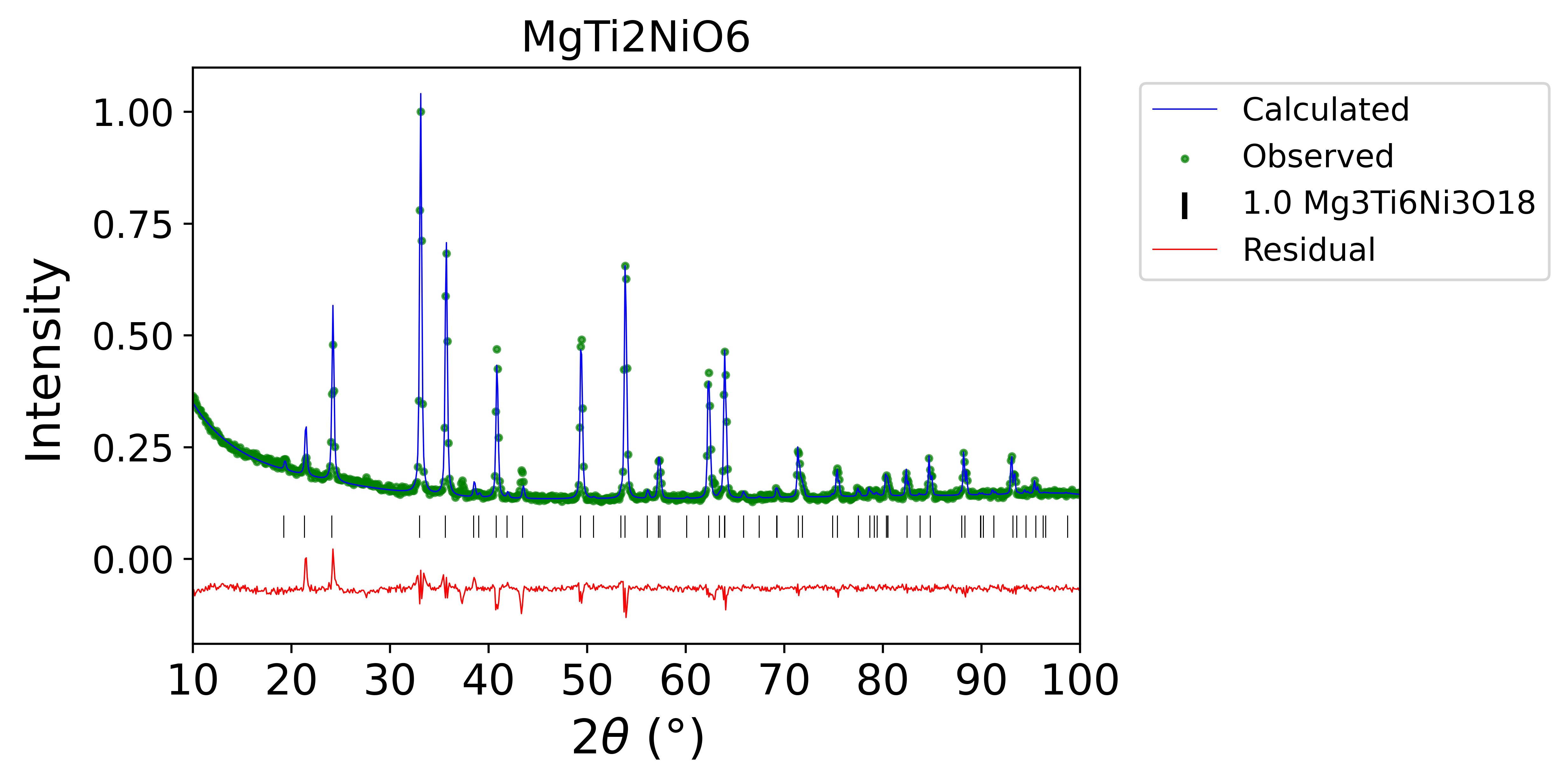

Supplement: Supplementary file 3 — This file contains the refined X-ray diffraction data from the successful syntheses performed by the A-Lab. The corresponding crystal structures used during refinement are also included in CIF format. [file 41586_2023_6734_MOESM3_ESM.zip › Automated_Refinement_Results/MgTi2NiO6/MgTi2NiO6_1000_240_MgO_NiO_TiO2_recipe38_4be64aa4-d110-4941-851f-0f6c759e5c05.jpg]

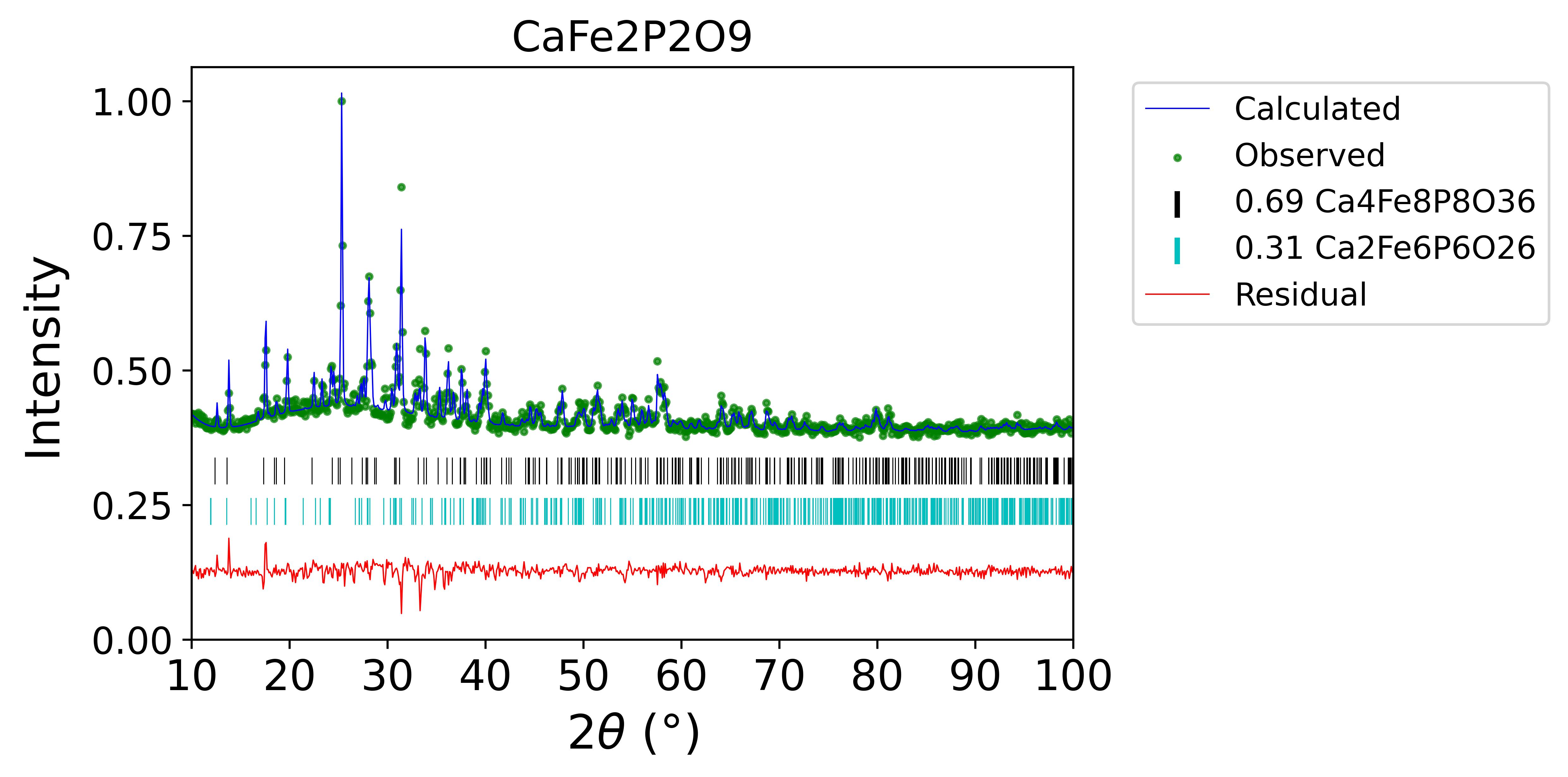

Supplement: Supplementary file 3 — This file contains the refined X-ray diffraction data from the successful syntheses performed by the A-Lab. The corresponding crystal structures used during refinement are also included in CIF format. [file 41586_2023_6734_MOESM3_ESM.zip › Automated_Refinement_Results/CaFe2P2O9/CaFe2P2O9_1000_240_CaO_Fe3O4_(NH4)2HPO4_ARRrecipe118_2b1a65b9-e800-4326-8056-0fd9f9df3a53.jpg]

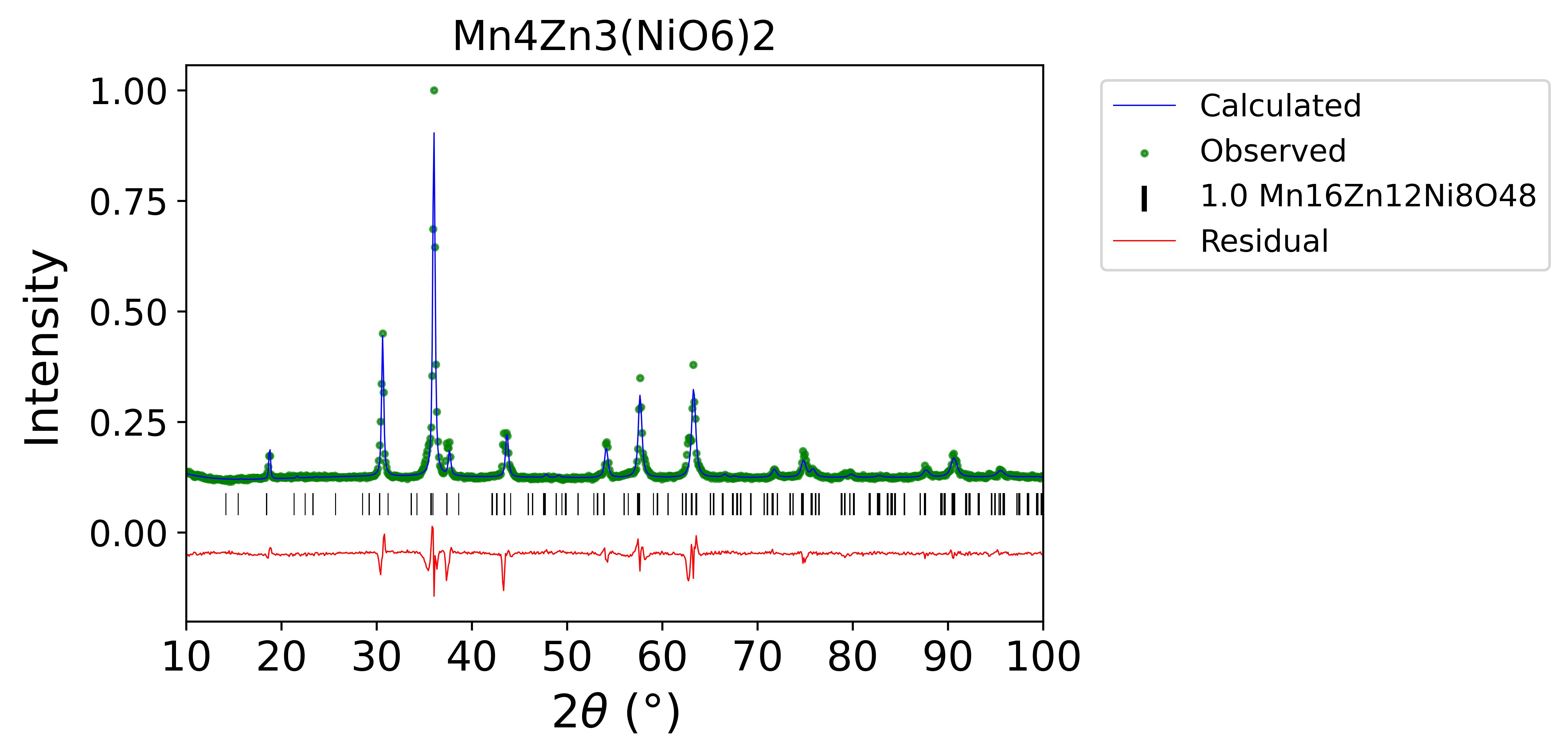

Supplement: Supplementary file 3 — This file contains the refined X-ray diffraction data from the successful syntheses performed by the A-Lab. The corresponding crystal structures used during refinement are also included in CIF format. [file 41586_2023_6734_MOESM3_ESM.zip › Automated_Refinement_Results/Mn4Zn3(NiO6)2/Mn4Zn3(NiO6)2_1000_240_Mn2O3_NiO_ZnO_recipe248_c919c8dd-5c3e-4511-bae3-eb045b46e731.jpg]

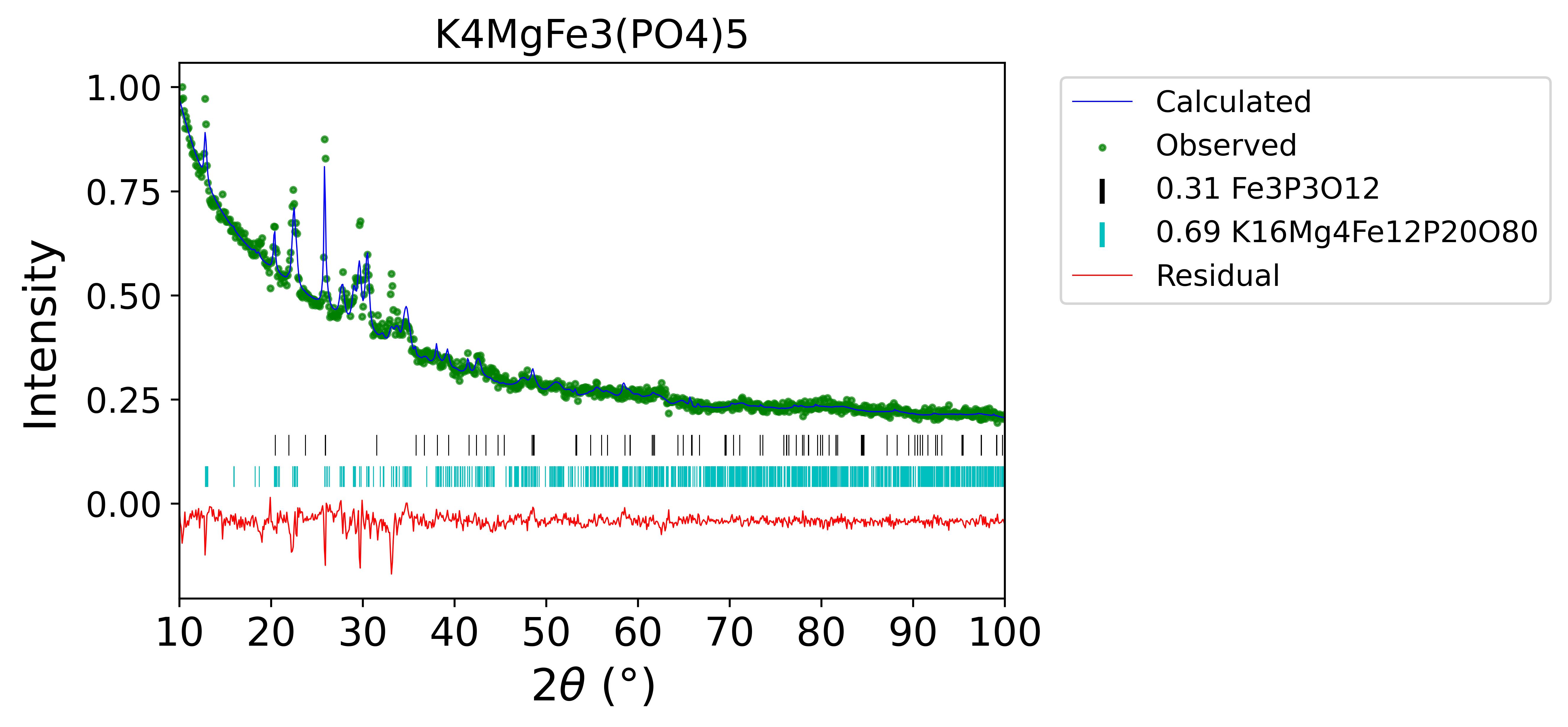

Supplement: Supplementary file 3 — This file contains the refined X-ray diffraction data from the successful syntheses performed by the A-Lab. The corresponding crystal structures used during refinement are also included in CIF format. [file 41586_2023_6734_MOESM3_ESM.zip › Automated_Refinement_Results/K4MgFe3(PO4)5/K4MgFe3(PO4)5_900_240_Fe2O3_K2CO3_(NH4)2HPO4_MgCO3_recipe48_manual.jpg]

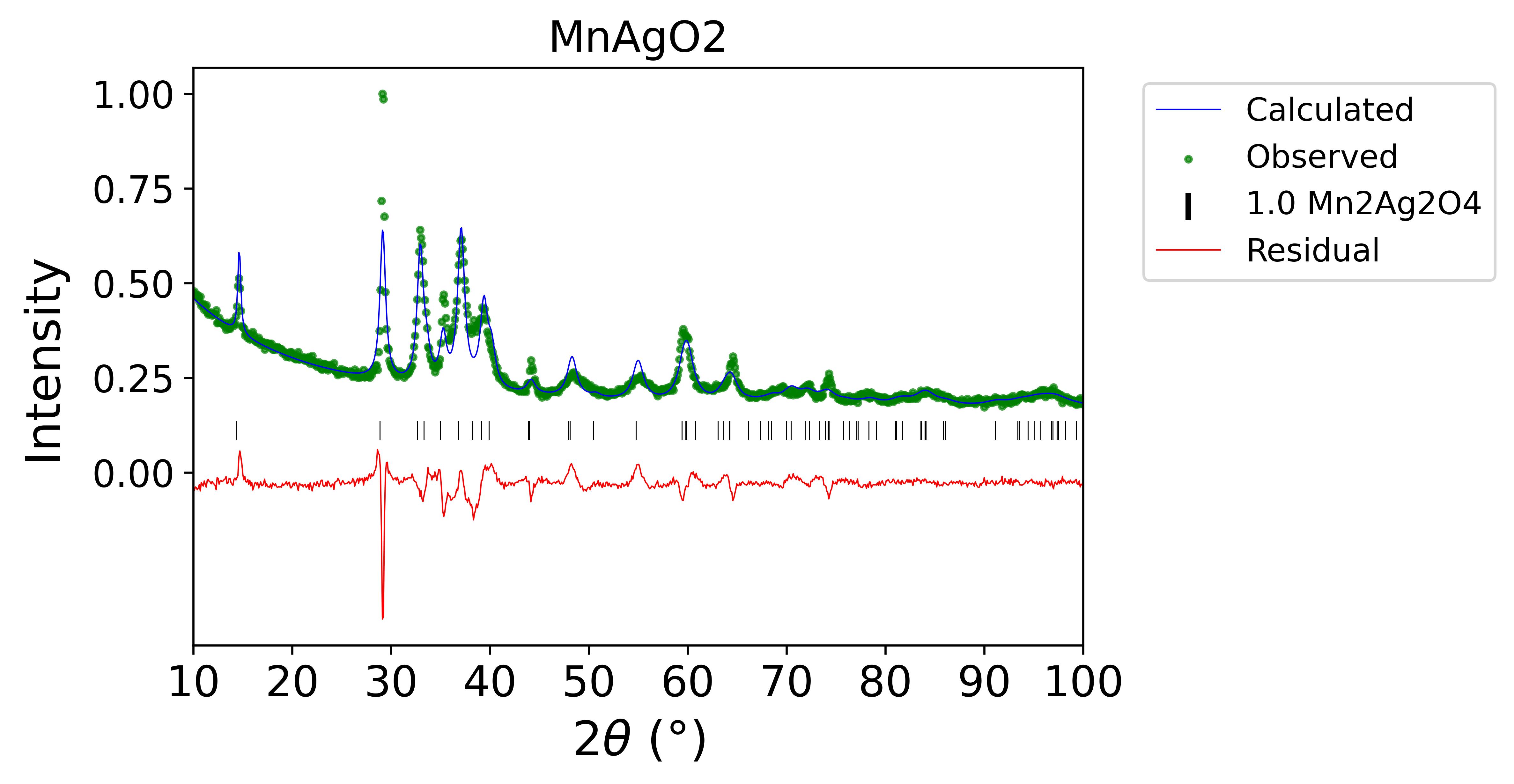

Supplement: Supplementary file 3 — This file contains the refined X-ray diffraction data from the successful syntheses performed by the A-Lab. The corresponding crystal structures used during refinement are also included in CIF format. [file 41586_2023_6734_MOESM3_ESM.zip › Automated_Refinement_Results/MnAgO2/MnAgO2_500_240_Ag2CO3_MnCO3_ARRrecipe78_ab5880bb-aa32-4024-906b-eaccde3d57e4.jpg]

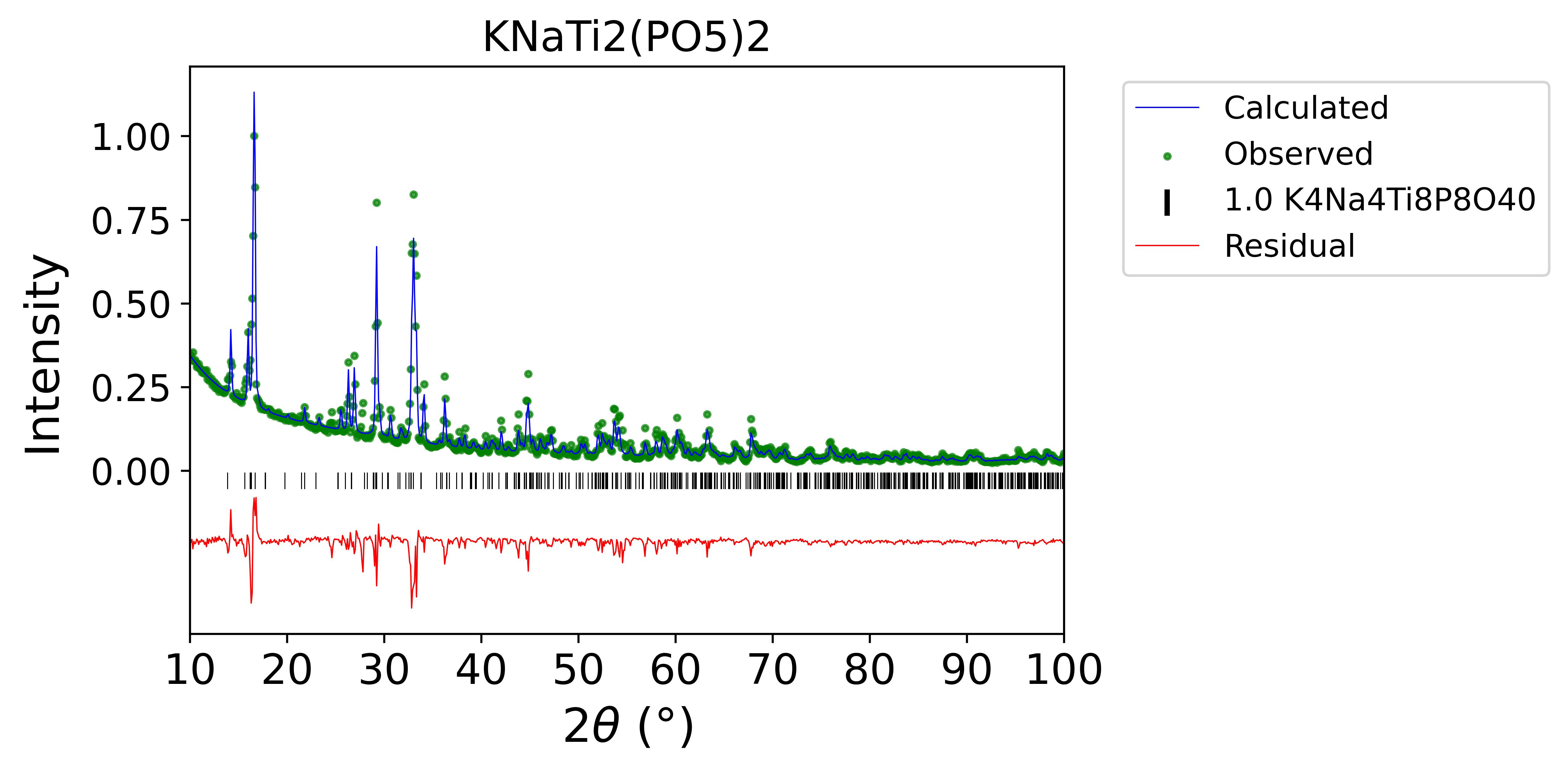

Supplement: Supplementary file 3 — This file contains the refined X-ray diffraction data from the successful syntheses performed by the A-Lab. The corresponding crystal structures used during refinement are also included in CIF format. [file 41586_2023_6734_MOESM3_ESM.zip › Automated_Refinement_Results/KNaTi2(PO5)2/KNaTi2(PO5)2_1000_240_NH4H2PO4_K2CO3_Na2CO3_TiO2_recipe73_4c1b7098-71eb-4332-8c6a-21f2ec375f83.jpg]

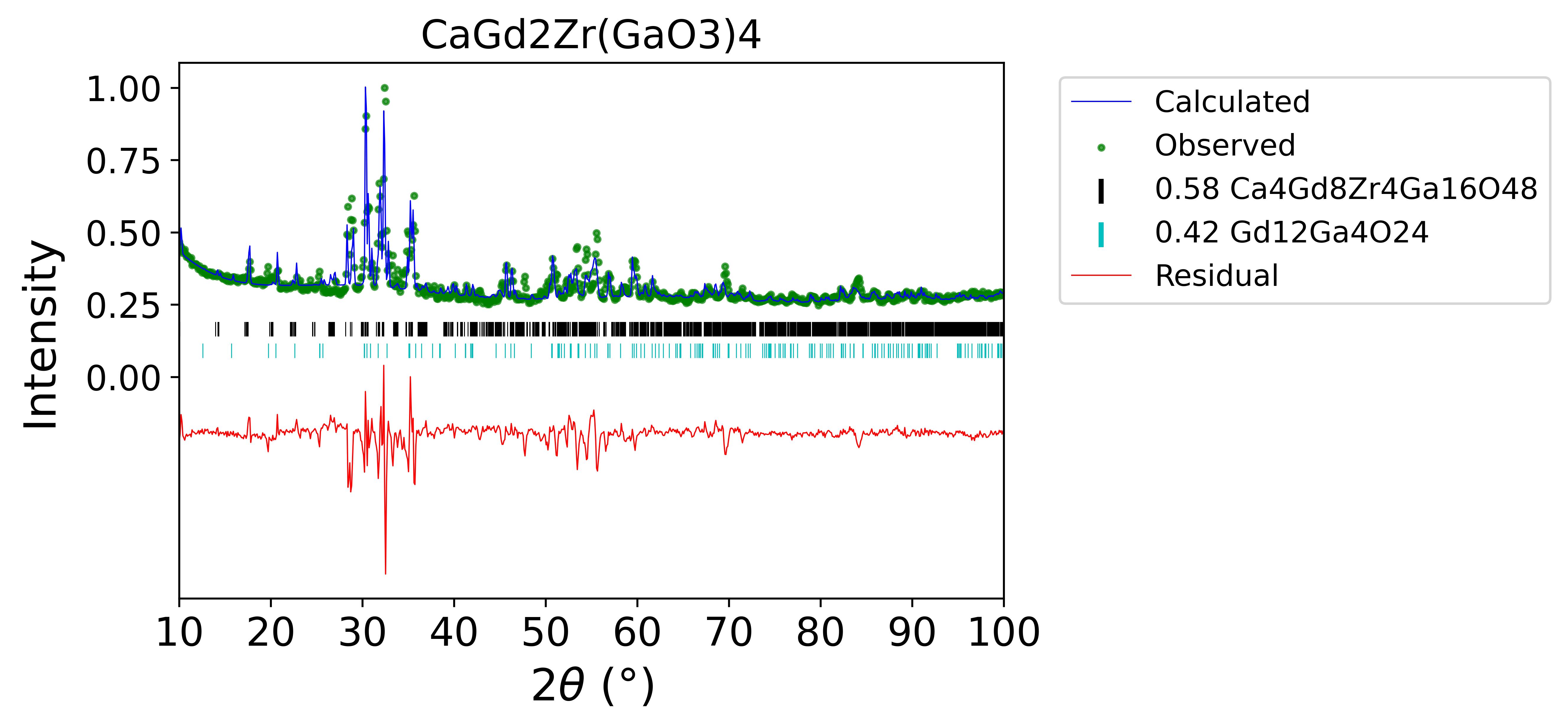

Supplement: Supplementary file 3 — This file contains the refined X-ray diffraction data from the successful syntheses performed by the A-Lab. The corresponding crystal structures used during refinement are also included in CIF format. [file 41586_2023_6734_MOESM3_ESM.zip › Automated_Refinement_Results/CaGd2Zr(GaO3)4/CaGd2Zr(GaO3)4_1100_240_Ca(OH)2_Ga2O3_Gd2O3_ZrO2_recipe350_213932c7-e70c-4eb2-bbaf-3658ff16d3ff.jpg]

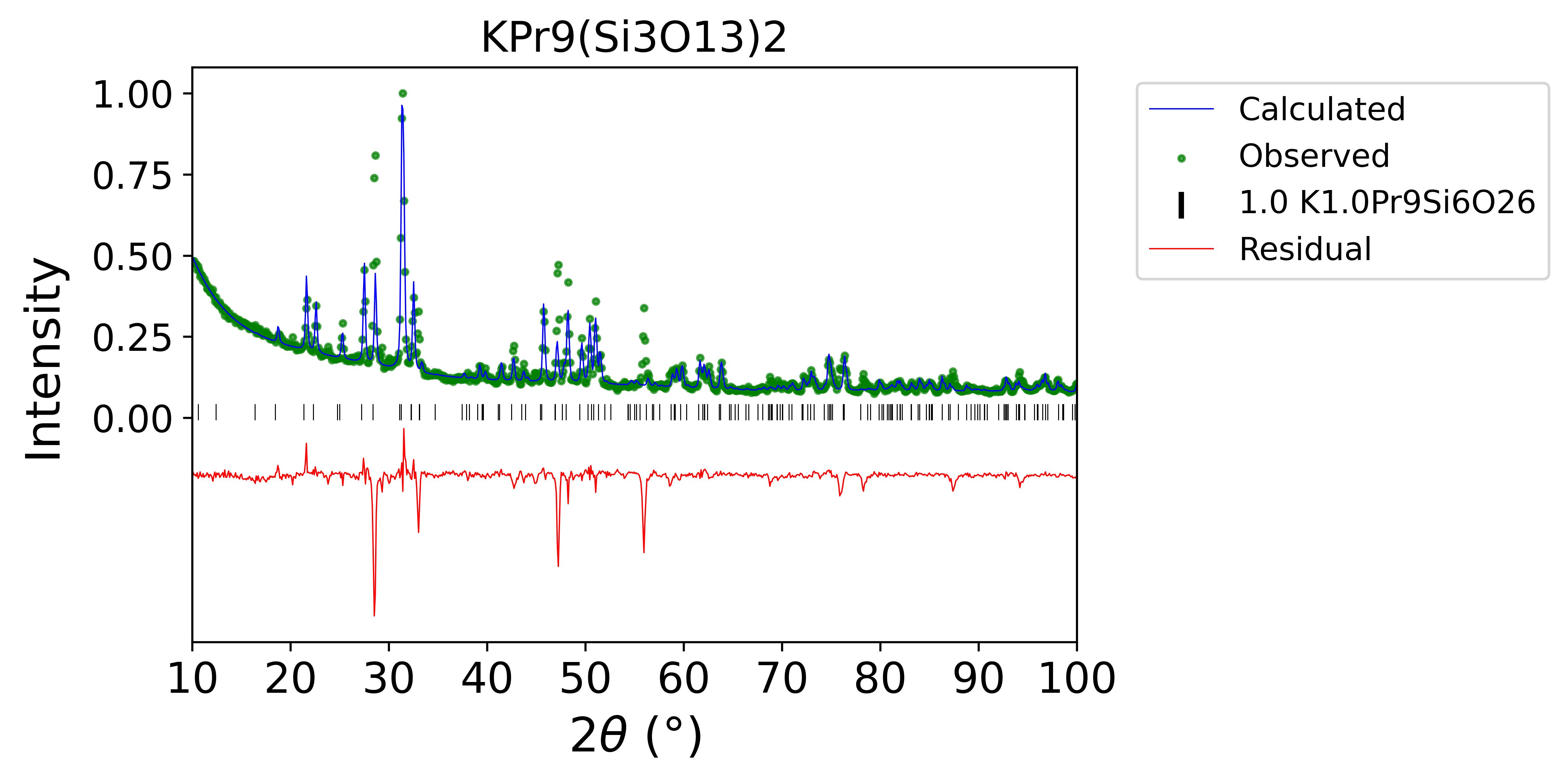

Supplement: Supplementary file 3 — This file contains the refined X-ray diffraction data from the successful syntheses performed by the A-Lab. The corresponding crystal structures used during refinement are also included in CIF format. [file 41586_2023_6734_MOESM3_ESM.zip › Automated_Refinement_Results/KPr9(Si3O13)2/KPr9(Si3O13)2_1100_240_K2CO3_Pr6O11_SiO2_recipe182_66e0b07e-a059-479d-b2d1-5a461494237a.jpg]

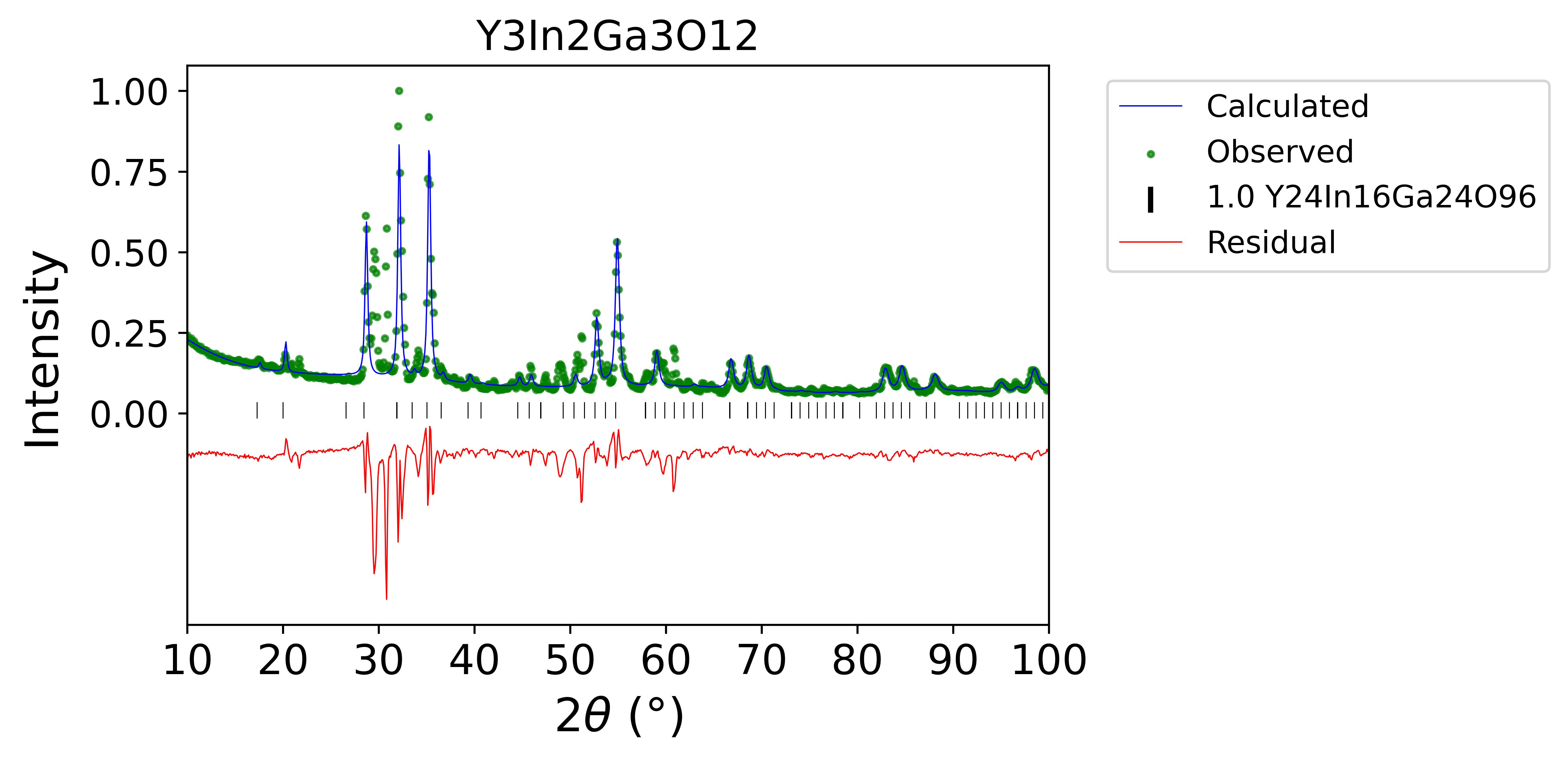

Supplement: Supplementary file 3 — This file contains the refined X-ray diffraction data from the successful syntheses performed by the A-Lab. The corresponding crystal structures used during refinement are also included in CIF format. [file 41586_2023_6734_MOESM3_ESM.zip › Automated_Refinement_Results/Y3Ga3In2O12/Y3In2Ga3O12_1100_240_Y2(CO3)3-3H2O_Ga2O3_In2O3_MPSIrecipe29.jpg]

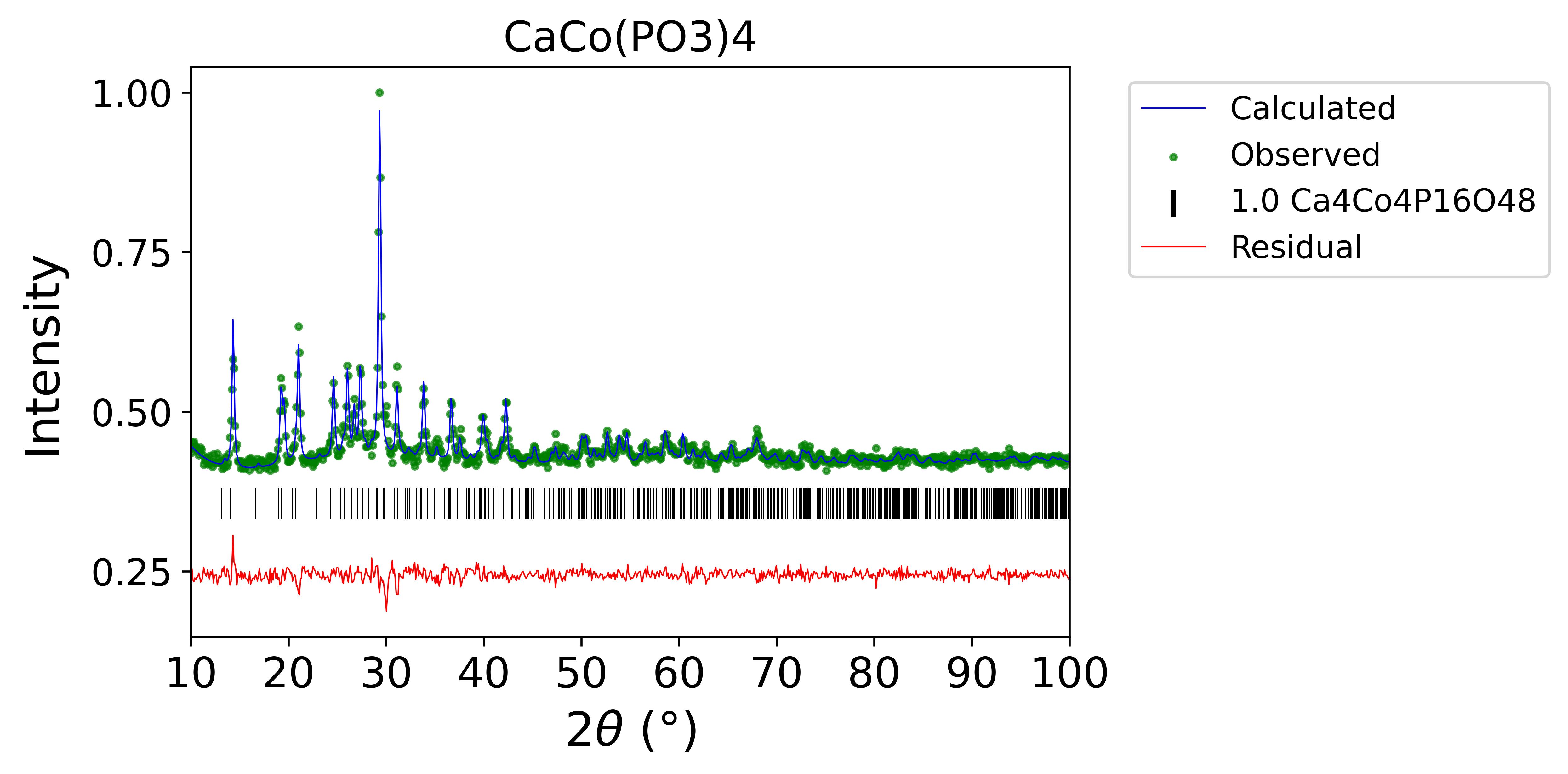

Supplement: Supplementary file 3 — This file contains the refined X-ray diffraction data from the successful syntheses performed by the A-Lab. The corresponding crystal structures used during refinement are also included in CIF format. [file 41586_2023_6734_MOESM3_ESM.zip › Automated_Refinement_Results/CaCo(PO3)4/CaCo(PO3)4_700_240_Ca(OH)2_CoO_(NH4)2HPO4_ARRrecipe187_2d79d6aa-4acd-4963-a9e6-94242517f093.jpg]

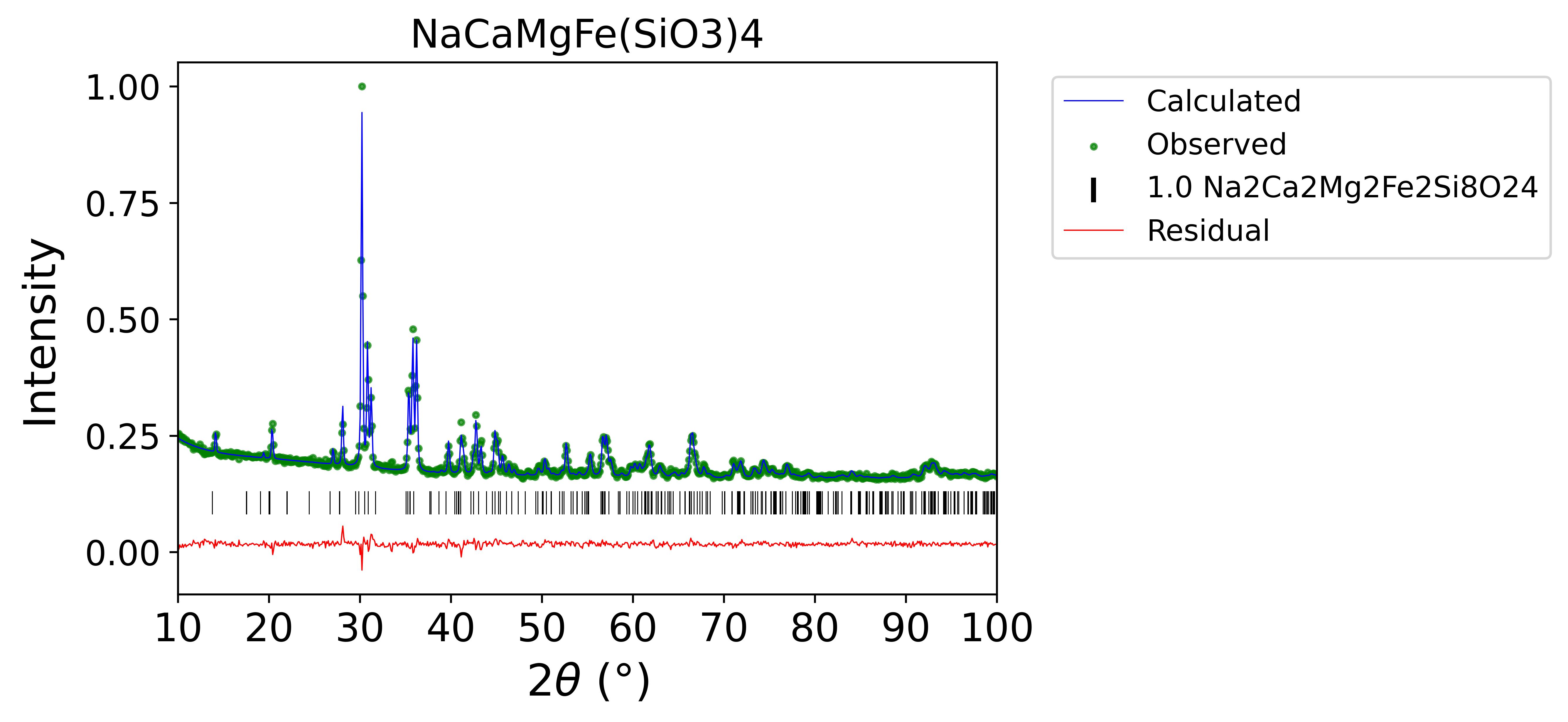

Supplement: Supplementary file 3 — This file contains the refined X-ray diffraction data from the successful syntheses performed by the A-Lab. The corresponding crystal structures used during refinement are also included in CIF format. [file 41586_2023_6734_MOESM3_ESM.zip › Automated_Refinement_Results/NaCaMgFe(SiO3)4/NaCaMgFe(SiO3)4_1000_240_CaCO3_Fe2O3_MgCO3_Na2CO3_SiO2_recipe218_b1c16479-0f4b-4710-bd38-484e415647d5.jpg]

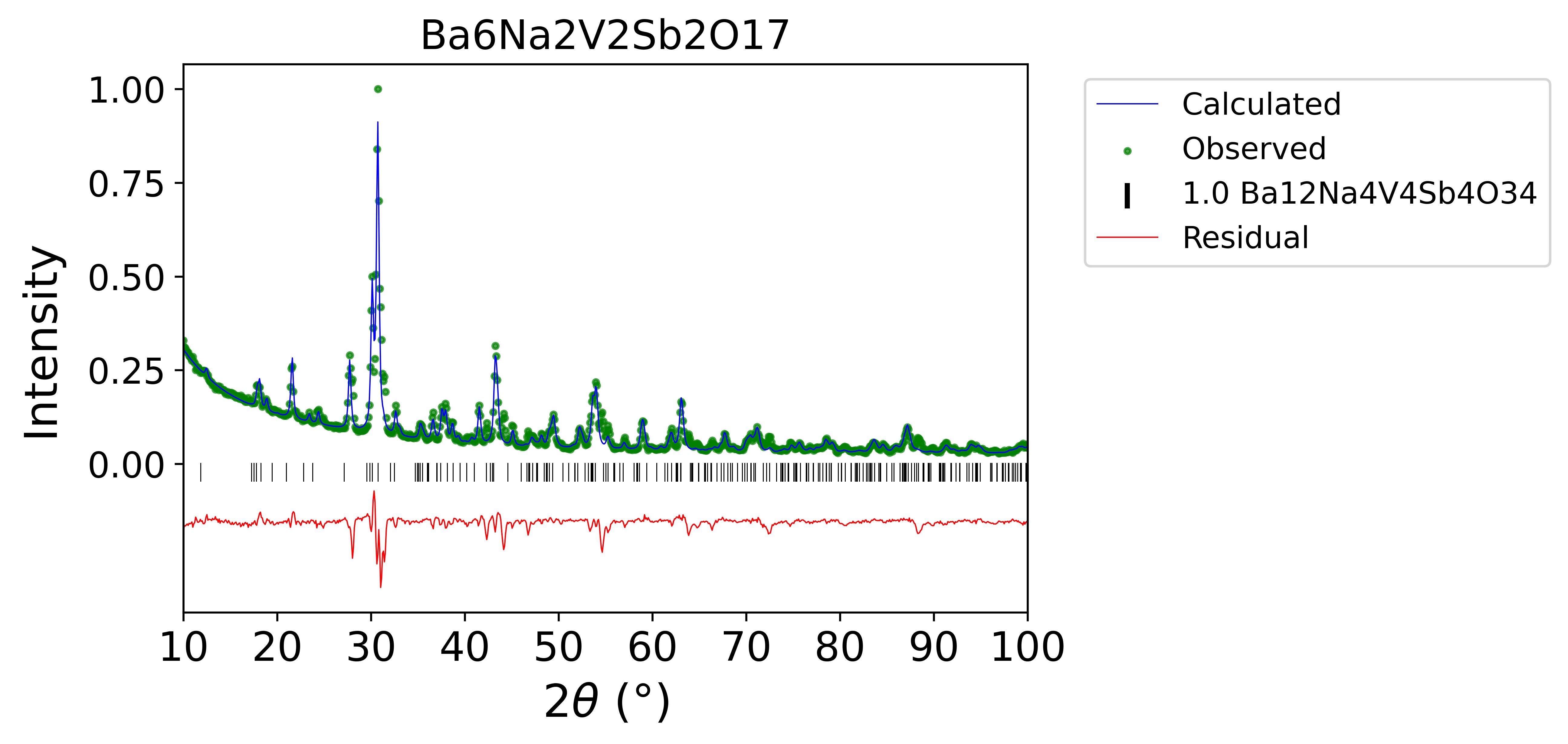

Supplement: Supplementary file 3 — This file contains the refined X-ray diffraction data from the successful syntheses performed by the A-Lab. The corresponding crystal structures used during refinement are also included in CIF format. [file 41586_2023_6734_MOESM3_ESM.zip › Automated_Refinement_Results/Ba6Na2V2Sb2O17/Ba6Na2V2Sb2O17_800_240_BaCO3_Na2CO3_Sb2O3_V2O5_recipe186_10b819ce-e49a-4bc6-9a1b-3f630994a2ef.jpg]

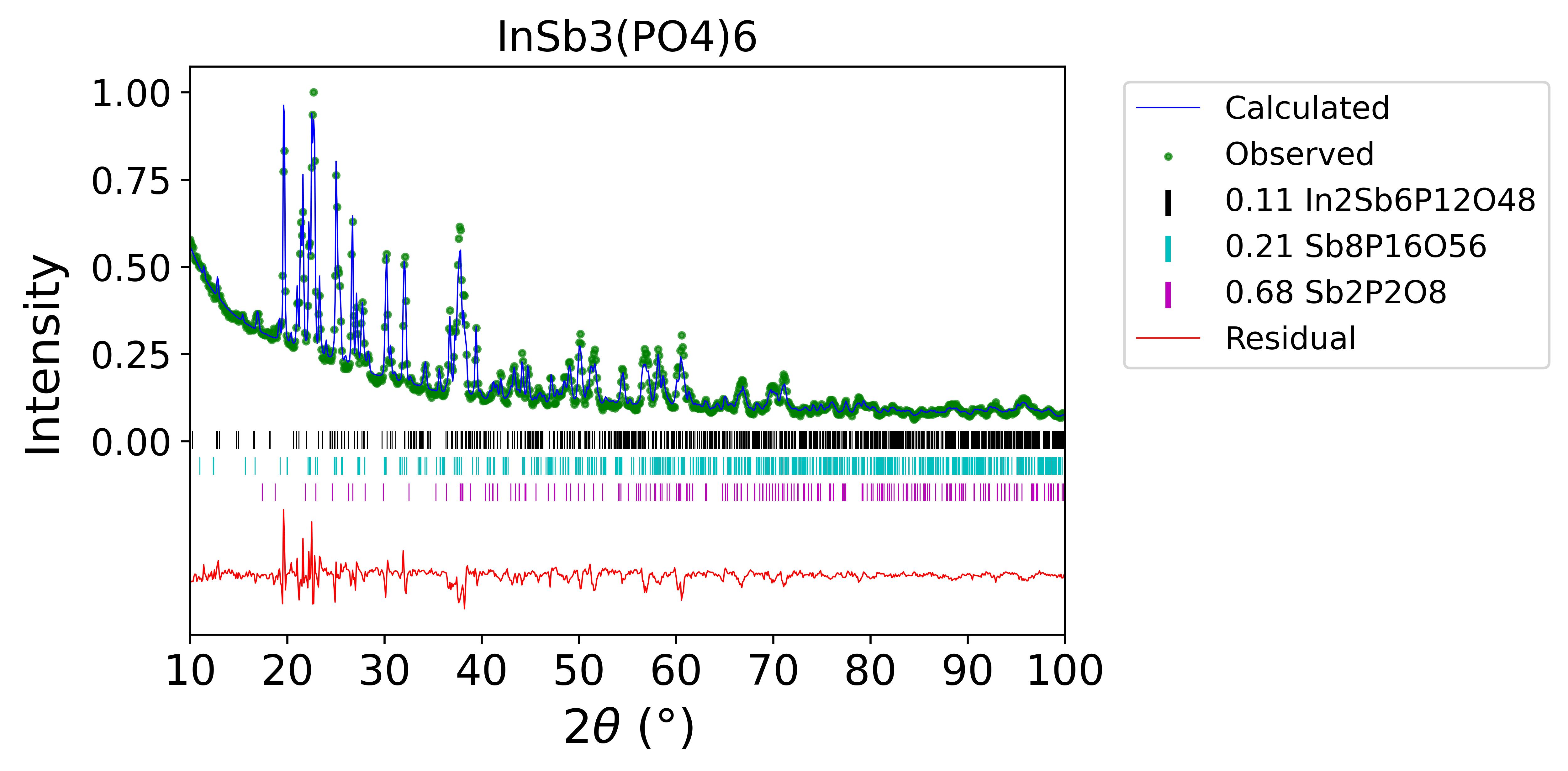

Supplement: Supplementary file 3 — This file contains the refined X-ray diffraction data from the successful syntheses performed by the A-Lab. The corresponding crystal structures used during refinement are also included in CIF format. [file 41586_2023_6734_MOESM3_ESM.zip › Automated_Refinement_Results/InSb3(PO4)6/InSb3(PO4)6_900_240_(NH4)2HPO4_In2O3_Sb2O5_recipe225_2d20a2d0-3782-4aa7-9dc9-e32ae80ee0bd.jpg]

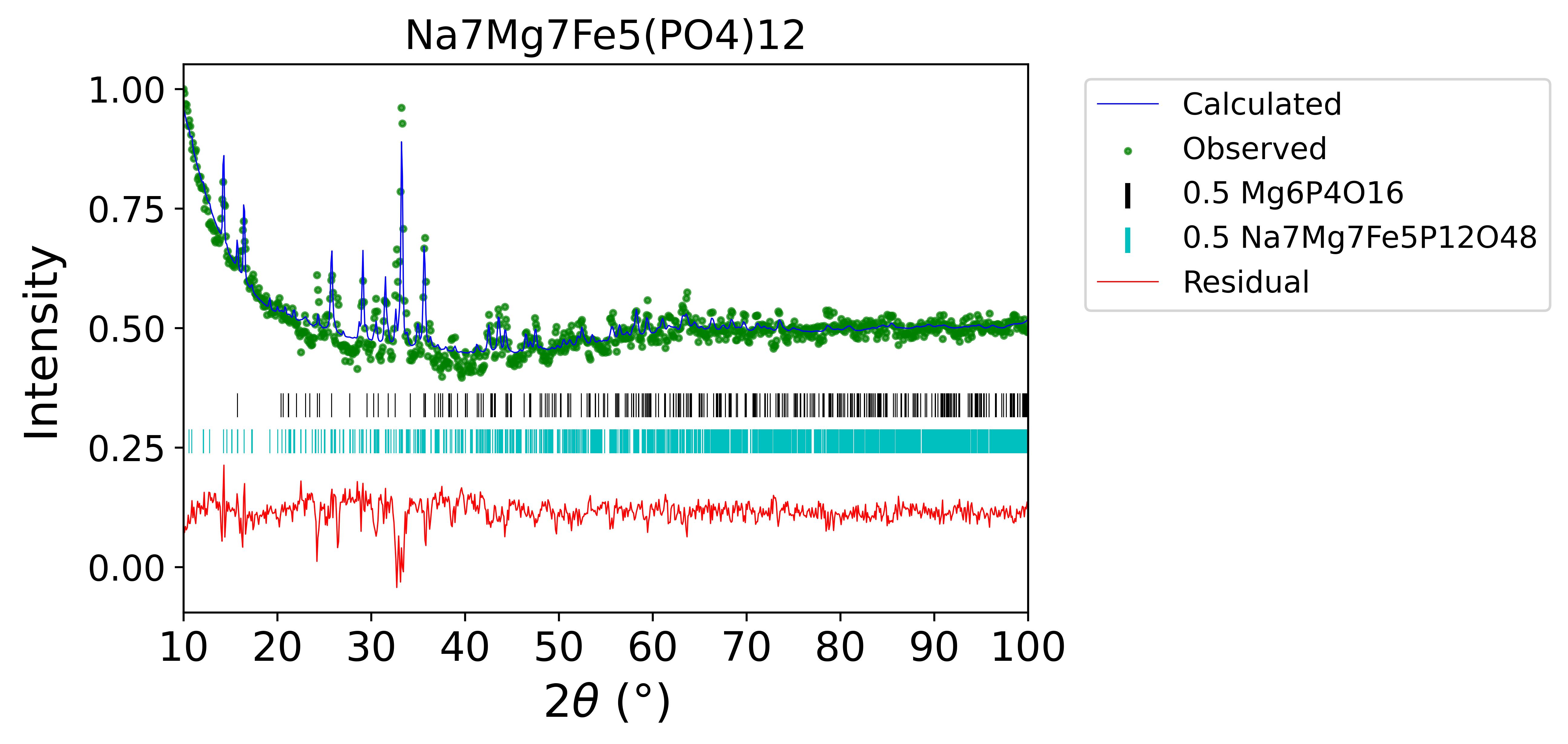

Supplement: Supplementary file 3 — This file contains the refined X-ray diffraction data from the successful syntheses performed by the A-Lab. The corresponding crystal structures used during refinement are also included in CIF format. [file 41586_2023_6734_MOESM3_ESM.zip › Automated_Refinement_Results/Na7Mg7Fe5(PO4)12/Na7Mg7Fe5(PO4)12_1000_240_Fe2O3_MgO_NH4H2PO4_Na2CO3_recipe67_manual.jpg]

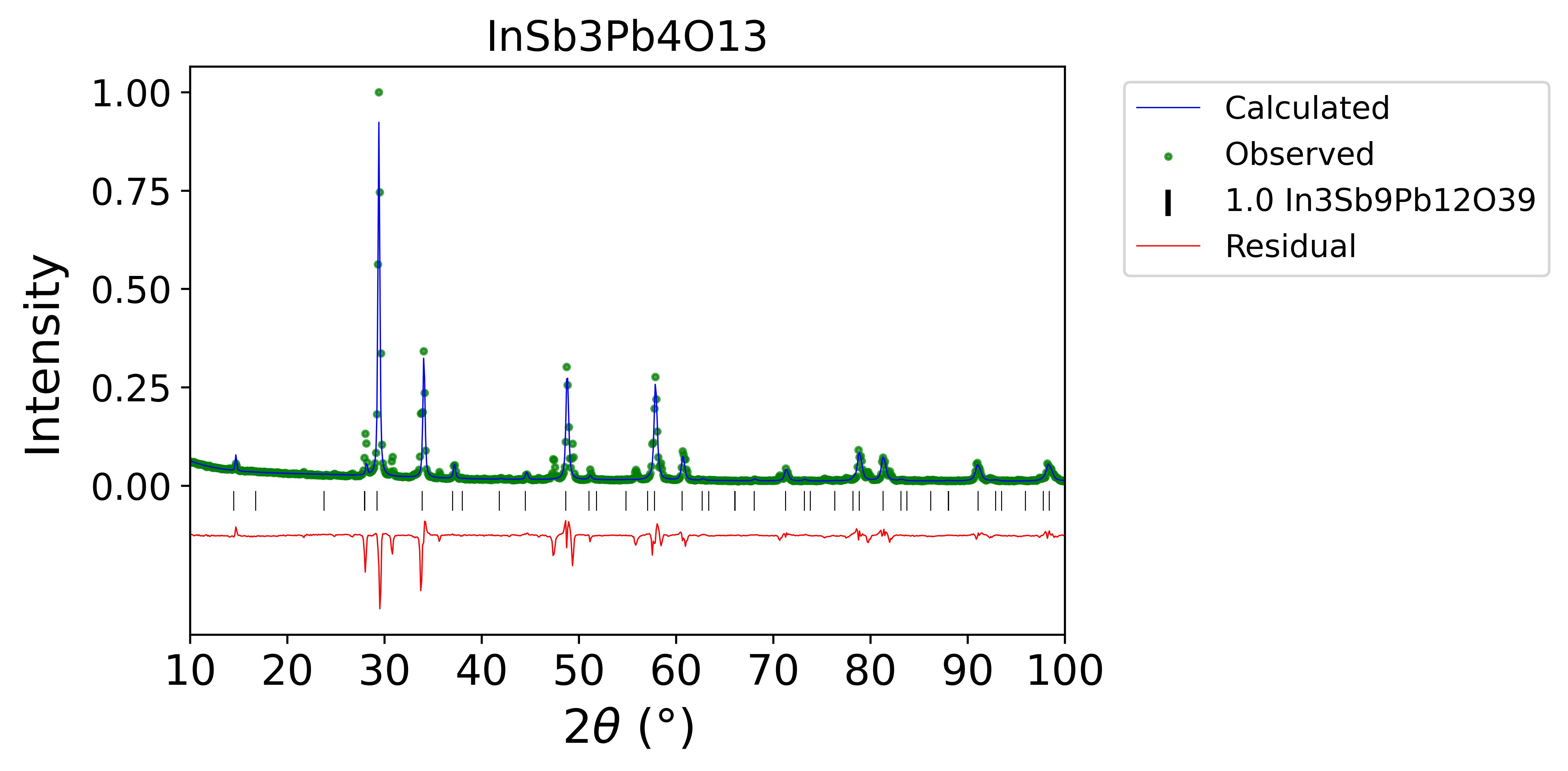

Supplement: Supplementary file 3 — This file contains the refined X-ray diffraction data from the successful syntheses performed by the A-Lab. The corresponding crystal structures used during refinement are also included in CIF format. [file 41586_2023_6734_MOESM3_ESM.zip › Automated_Refinement_Results/InSb3Pb4O13/InSb3Pb4O13_900_240_In2O3_PbO_Sb2O3_recipe219_c18d8a94-5be9-4595-a35f-54b5658c5559.jpg]

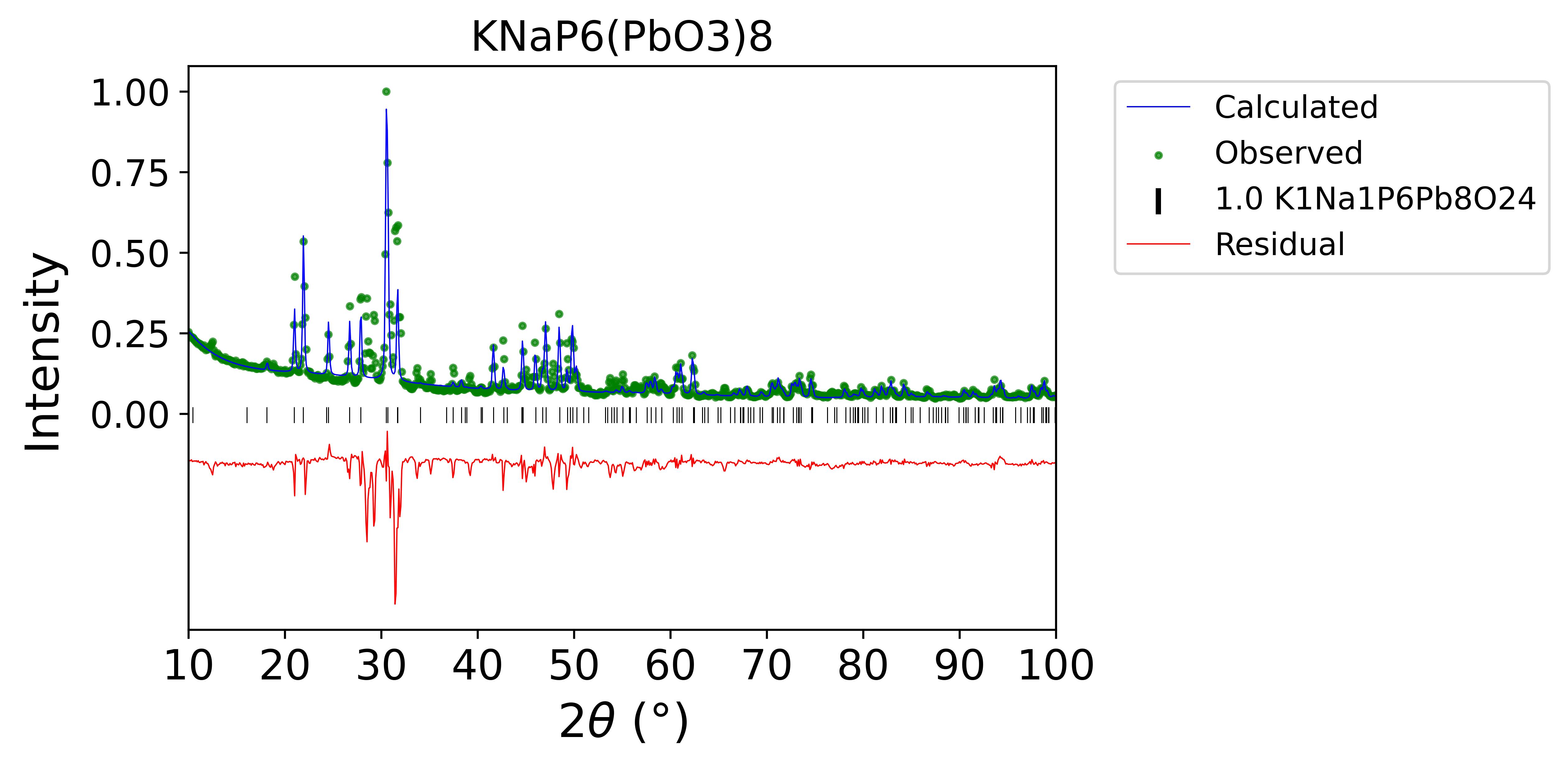

Supplement: Supplementary file 3 — This file contains the refined X-ray diffraction data from the successful syntheses performed by the A-Lab. The corresponding crystal structures used during refinement are also included in CIF format. [file 41586_2023_6734_MOESM3_ESM.zip › Automated_Refinement_Results/KNaP6(PbO3)8/KNaP6(PbO3)8_1000_240_K2CO3_(NH4)2HPO4_Na2CO3_PbO_recipe339_0d018a80-ae76-434d-8875-daffd32284ef.jpg]

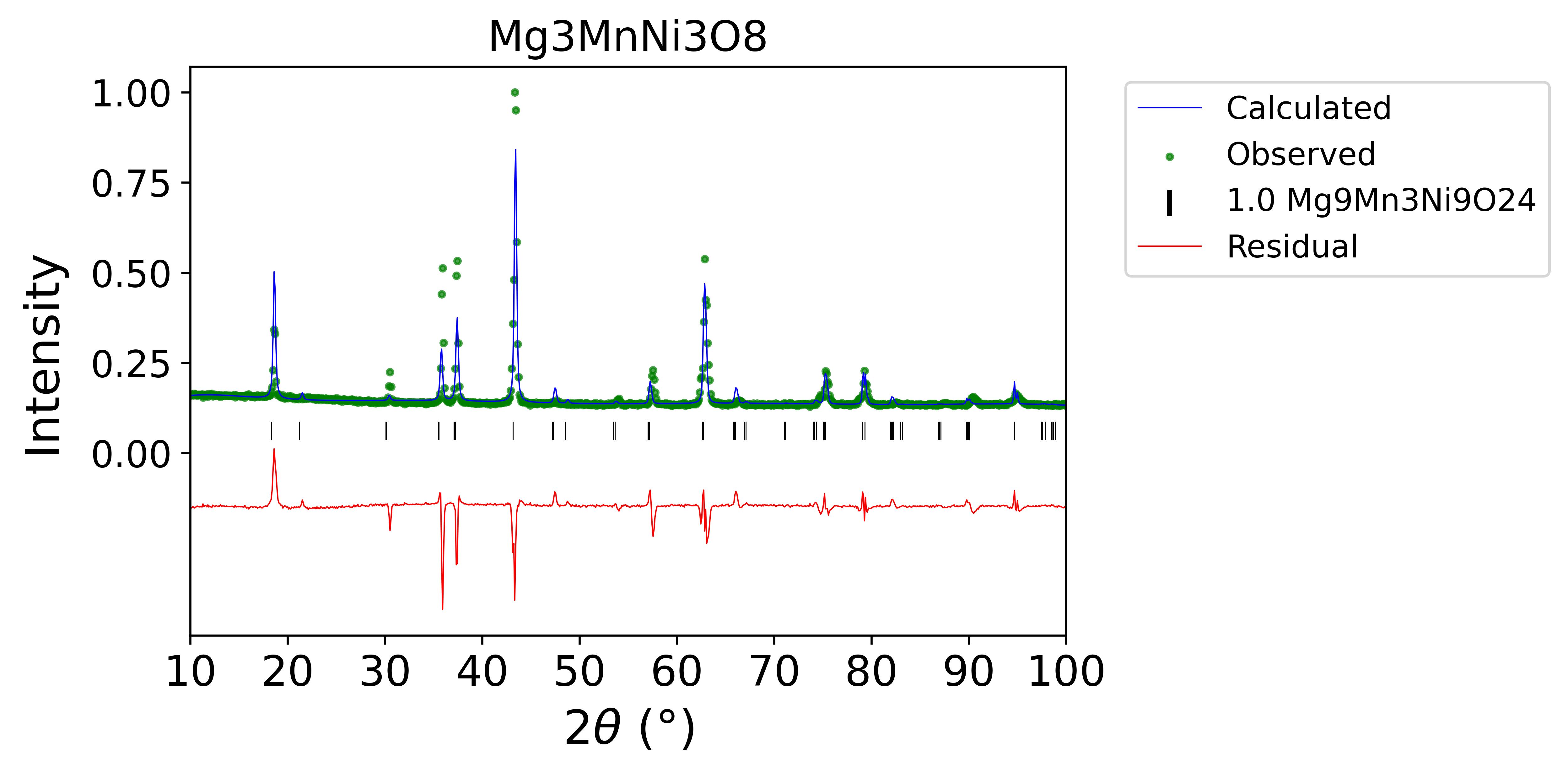

Supplement: Supplementary file 3 — This file contains the refined X-ray diffraction data from the successful syntheses performed by the A-Lab. The corresponding crystal structures used during refinement are also included in CIF format. [file 41586_2023_6734_MOESM3_ESM.zip › Automated_Refinement_Results/Mg3MnNi3O8/Mg3MnNi3O8_900_240_MgO_MnO2_NiO_recipe1_aee64223-2165-48c7-ace0-28dec55892a4.jpg]

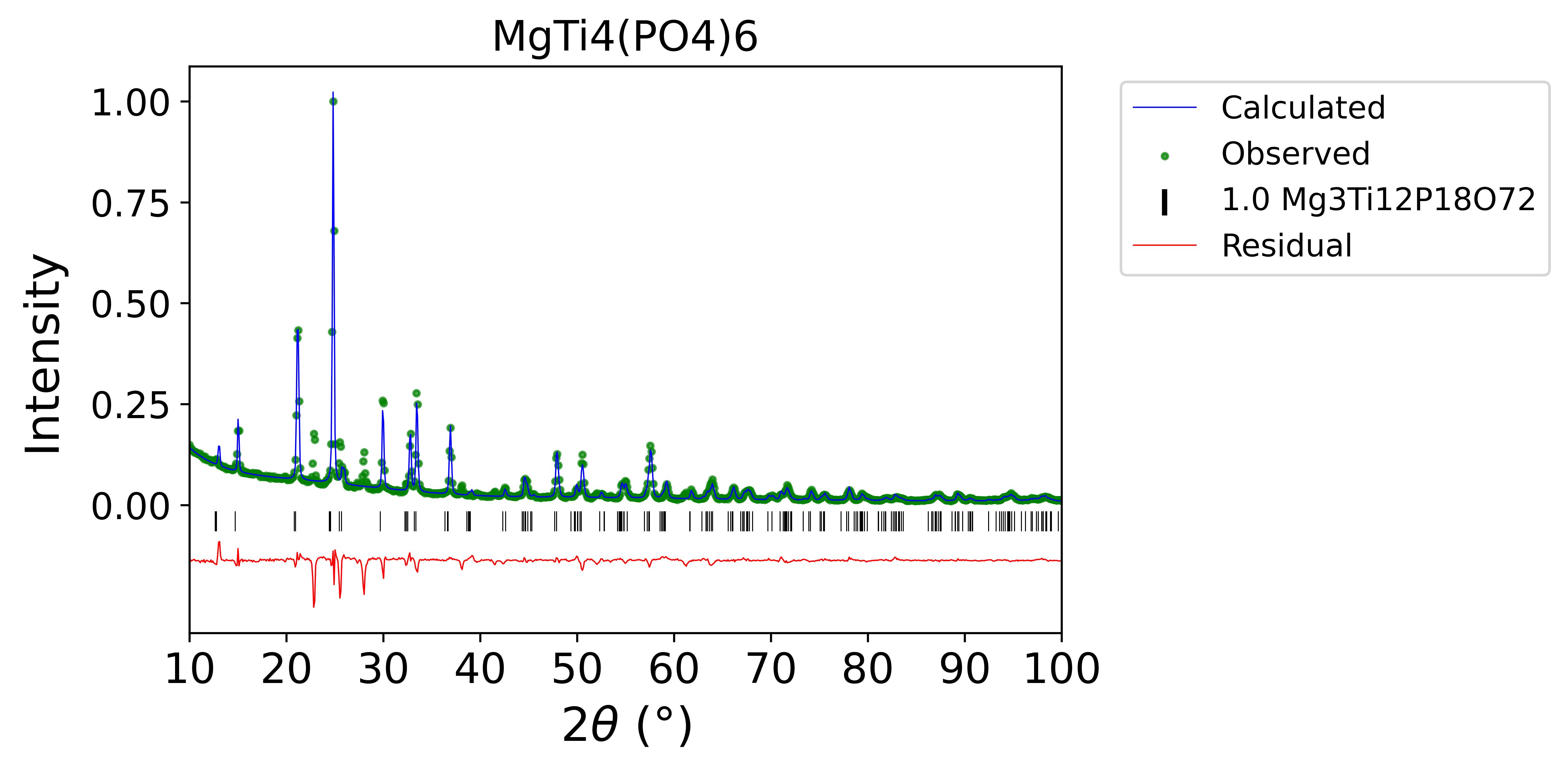

Supplement: Supplementary file 3 — This file contains the refined X-ray diffraction data from the successful syntheses performed by the A-Lab. The corresponding crystal structures used during refinement are also included in CIF format. [file 41586_2023_6734_MOESM3_ESM.zip › Automated_Refinement_Results/MgTi4(PO4)6/MgTi4(PO4)6_1000_240_MgCO3_NH4H2PO4_TiO2_recipe198_afaace16-bfa6-4d88-a8fc-7991f82bb307.jpg]

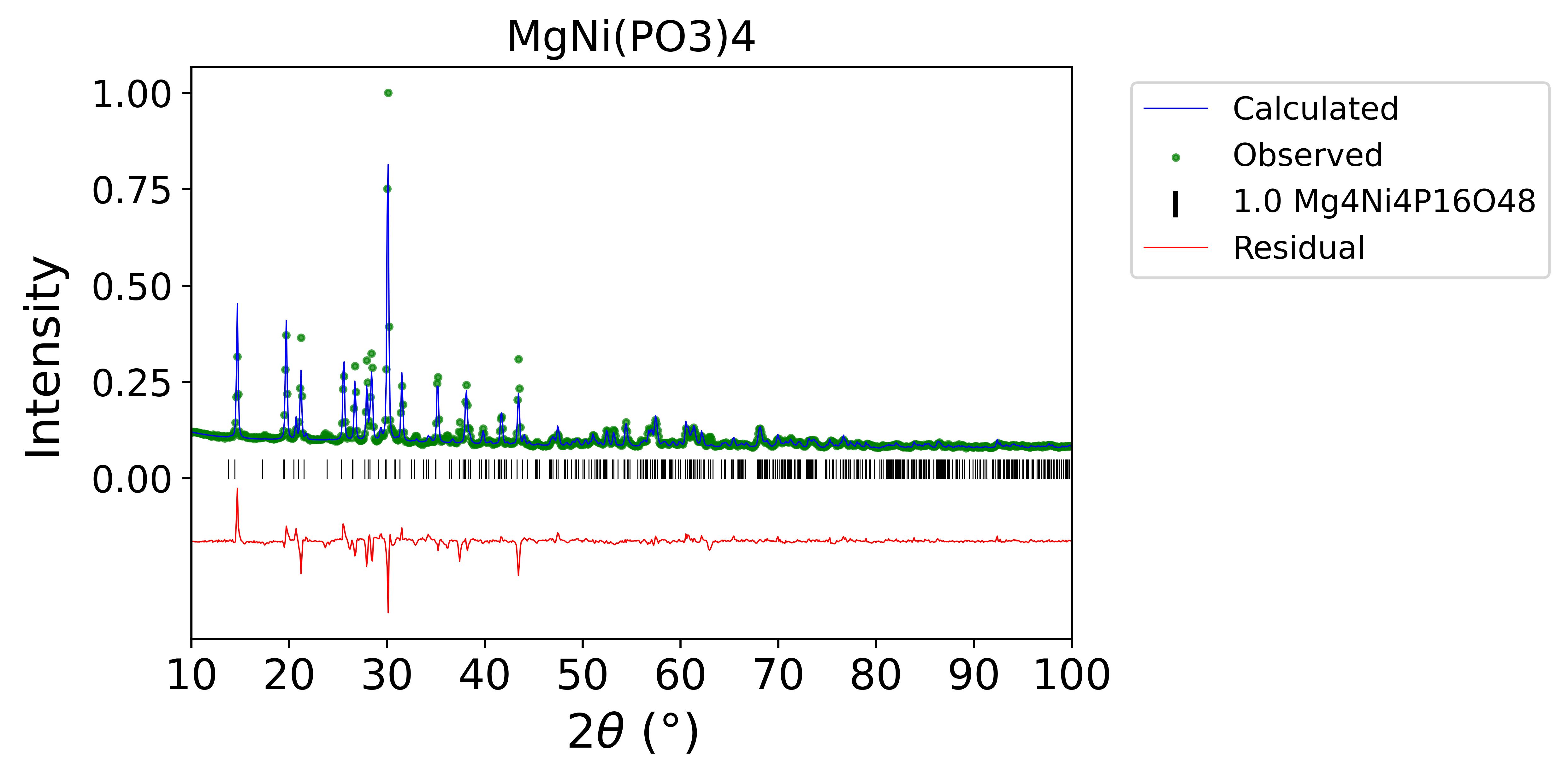

Supplement: Supplementary file 3 — This file contains the refined X-ray diffraction data from the successful syntheses performed by the A-Lab. The corresponding crystal structures used during refinement are also included in CIF format. [file 41586_2023_6734_MOESM3_ESM.zip › Automated_Refinement_Results/MgNi(PO3)4/MgNi(PO3)4_900_240_MgO_(NH4)2HPO4_NiO_recipe11_5da91ded-2287-4923-baa3-0ad27c59cea6.jpg]

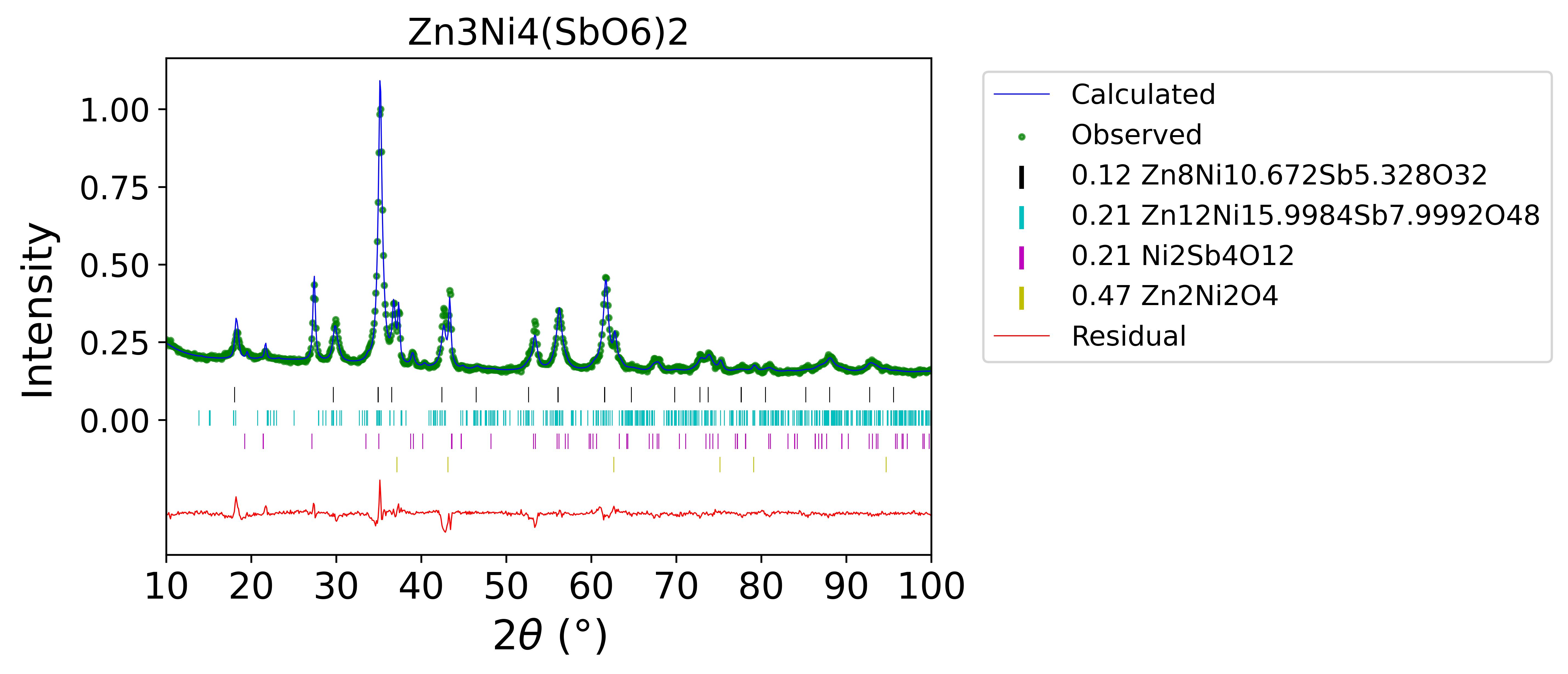

Supplement: Supplementary file 3 — This file contains the refined X-ray diffraction data from the successful syntheses performed by the A-Lab. The corresponding crystal structures used during refinement are also included in CIF format. [file 41586_2023_6734_MOESM3_ESM.zip › Automated_Refinement_Results/Zn3Ni4(SbO6)2/Zn3Ni4(SbO6)2_900_240_Ni(OH)2_Sb2O3_ZnO_recipe138_f3ca1d77-a01d-4086-a8e8-2868b1719823.jpg]

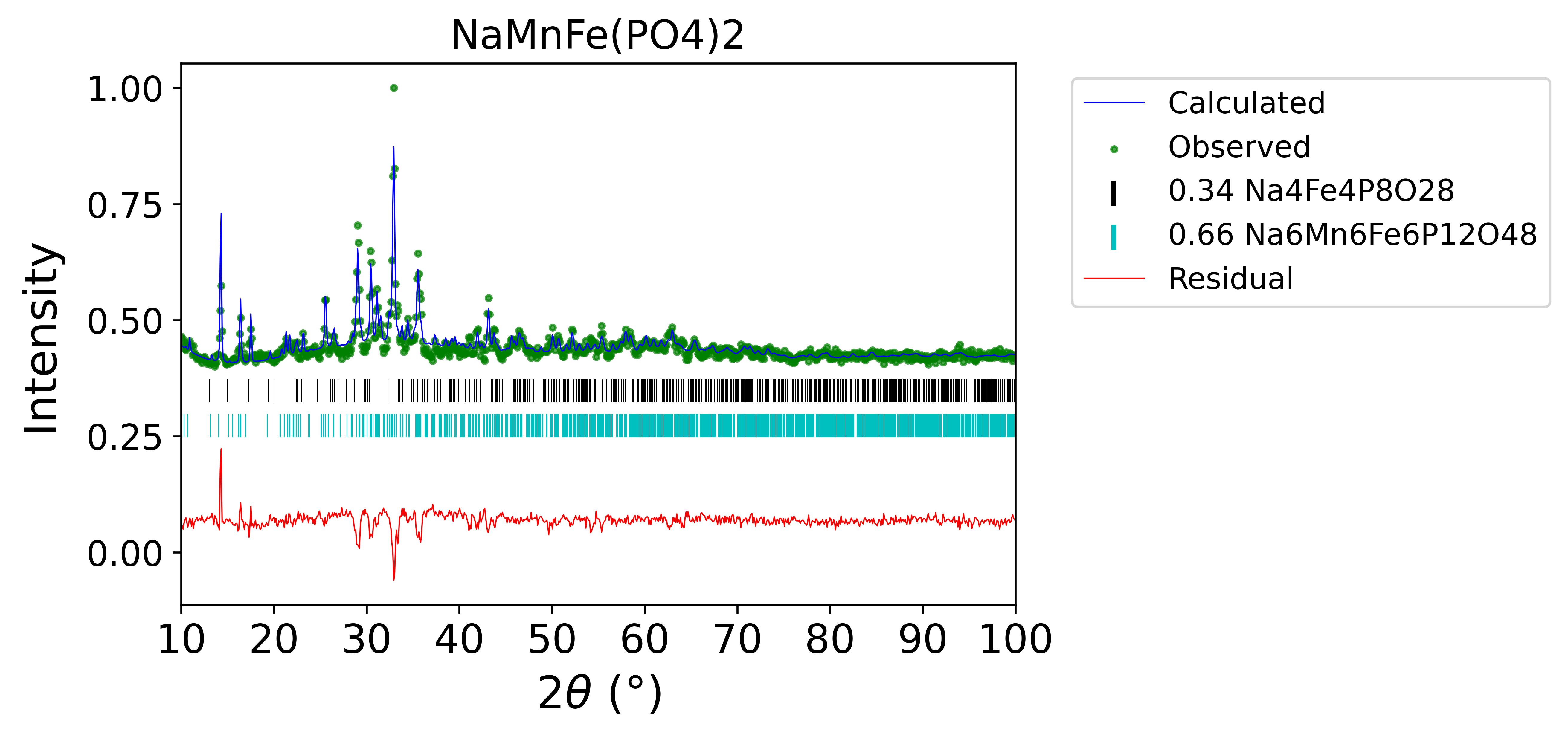

Supplement: Supplementary file 3 — This file contains the refined X-ray diffraction data from the successful syntheses performed by the A-Lab. The corresponding crystal structures used during refinement are also included in CIF format. [file 41586_2023_6734_MOESM3_ESM.zip › Automated_Refinement_Results/NaMnFe(PO4)2/NaMnFe(PO4)2_700_240_Fe2O3_MnO2_NH4H2PO4_Na2CO3_recipe62_fb416271-89b6-409f-b520-d92d61ae051e.jpg]

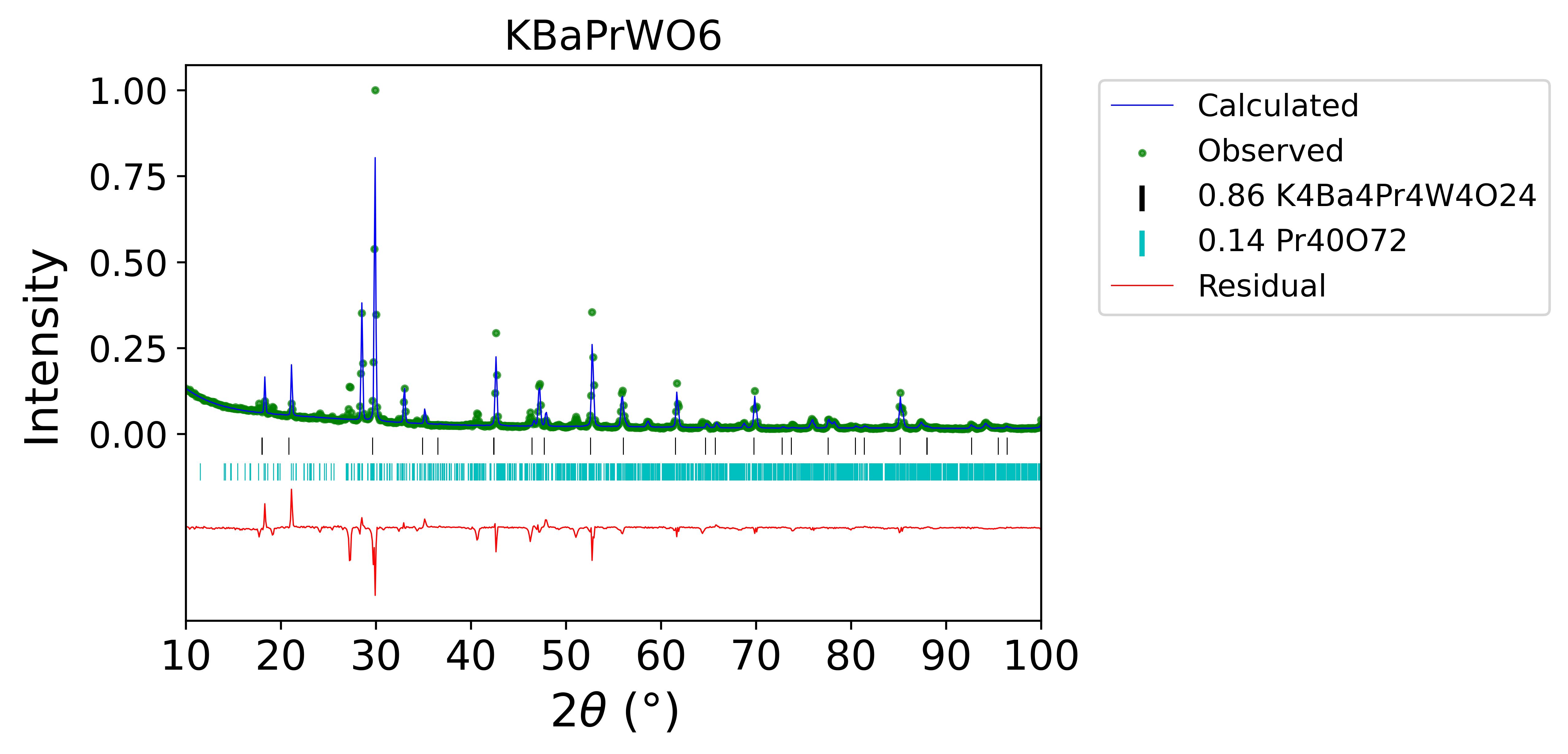

Supplement: Supplementary file 3 — This file contains the refined X-ray diffraction data from the successful syntheses performed by the A-Lab. The corresponding crystal structures used during refinement are also included in CIF format. [file 41586_2023_6734_MOESM3_ESM.zip › Automated_Refinement_Results/KBaPrWO6/KBaPrWO6_1000_240_BaO2_K2CO3_Pr6O11_WO3_recipe269_92a16fa1-7ab6-4d6c-8d49-03e4da408335.jpg]

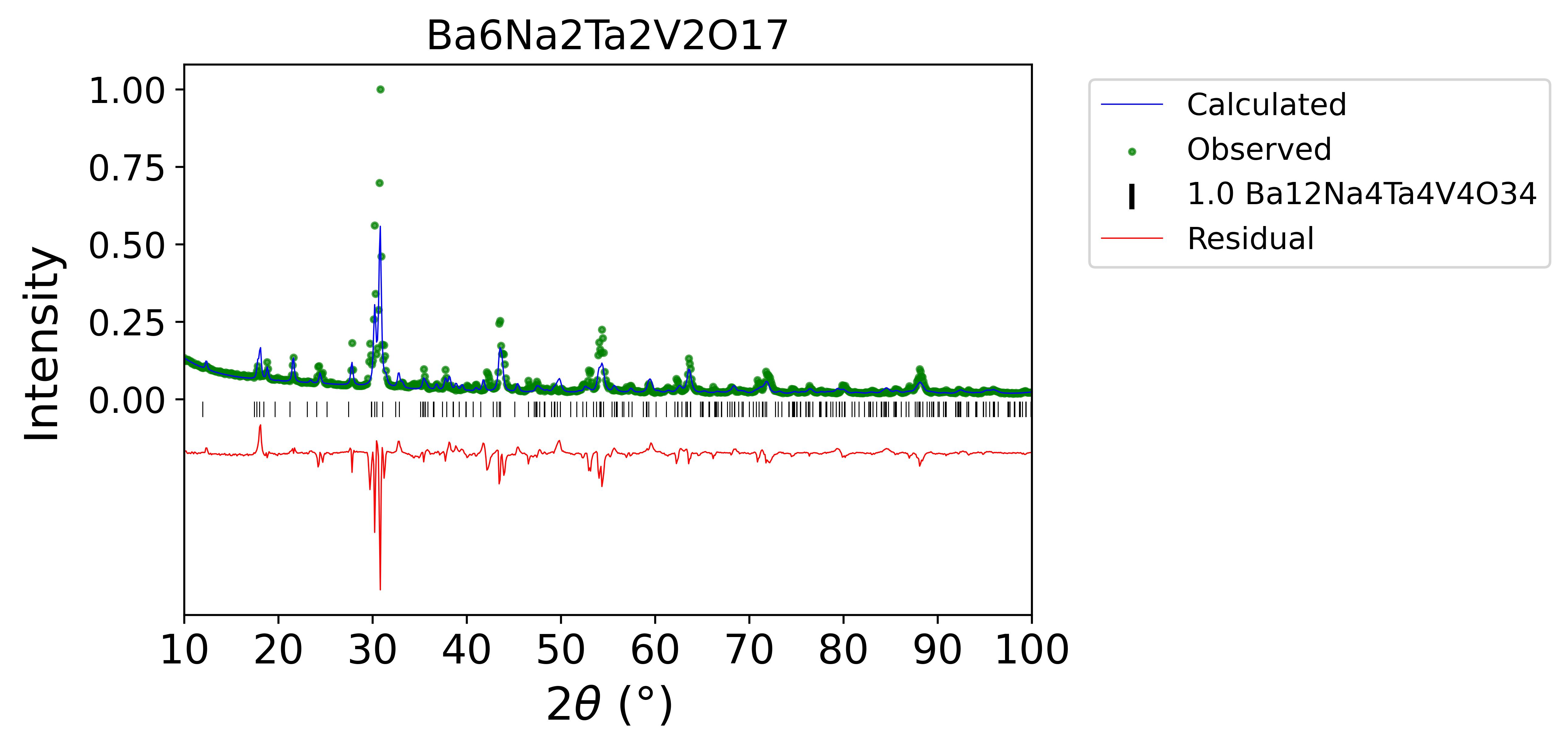

Supplement: Supplementary file 3 — This file contains the refined X-ray diffraction data from the successful syntheses performed by the A-Lab. The corresponding crystal structures used during refinement are also included in CIF format. [file 41586_2023_6734_MOESM3_ESM.zip › Automated_Refinement_Results/Ba6Na2Ta2V2O17/Ba6Na2Ta2V2O17_900_240_BaCO3_Na2CO3_Ta2O5_V2O5_recipe252_1119fc0c-8822-4c3f-a855-0b24fedb887c.jpg]

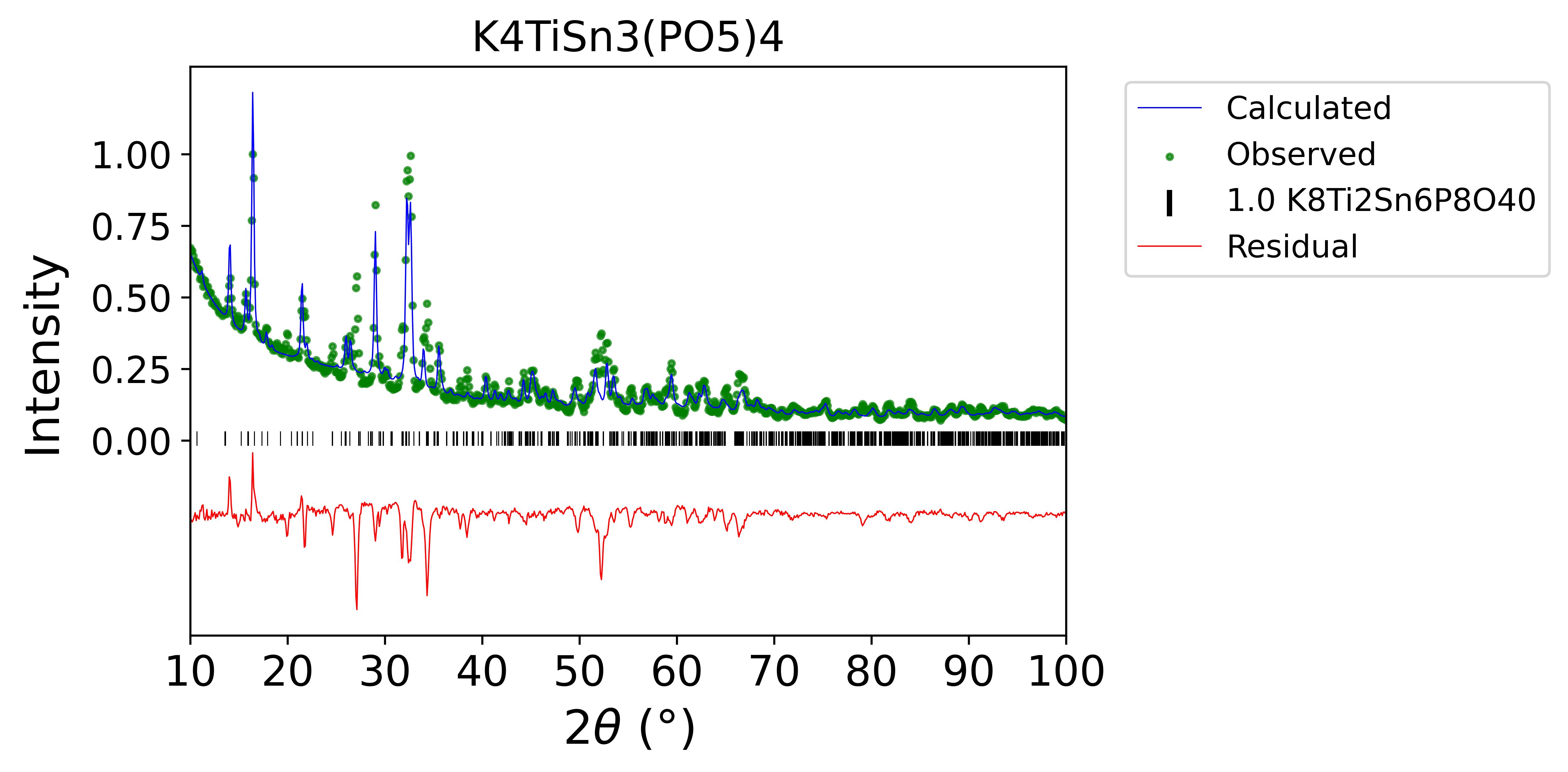

Supplement: Supplementary file 3 — This file contains the refined X-ray diffraction data from the successful syntheses performed by the A-Lab. The corresponding crystal structures used during refinement are also included in CIF format. [file 41586_2023_6734_MOESM3_ESM.zip › Automated_Refinement_Results/K4TiSn3(PO5)4/K4TiSn3(PO5)4_1100_240_NH4H2PO4_K2CO3_SnO2_TiO2_recipe199_21a683dd-3325-4cf2-be11-c1d3f6b1ab72.jpg]

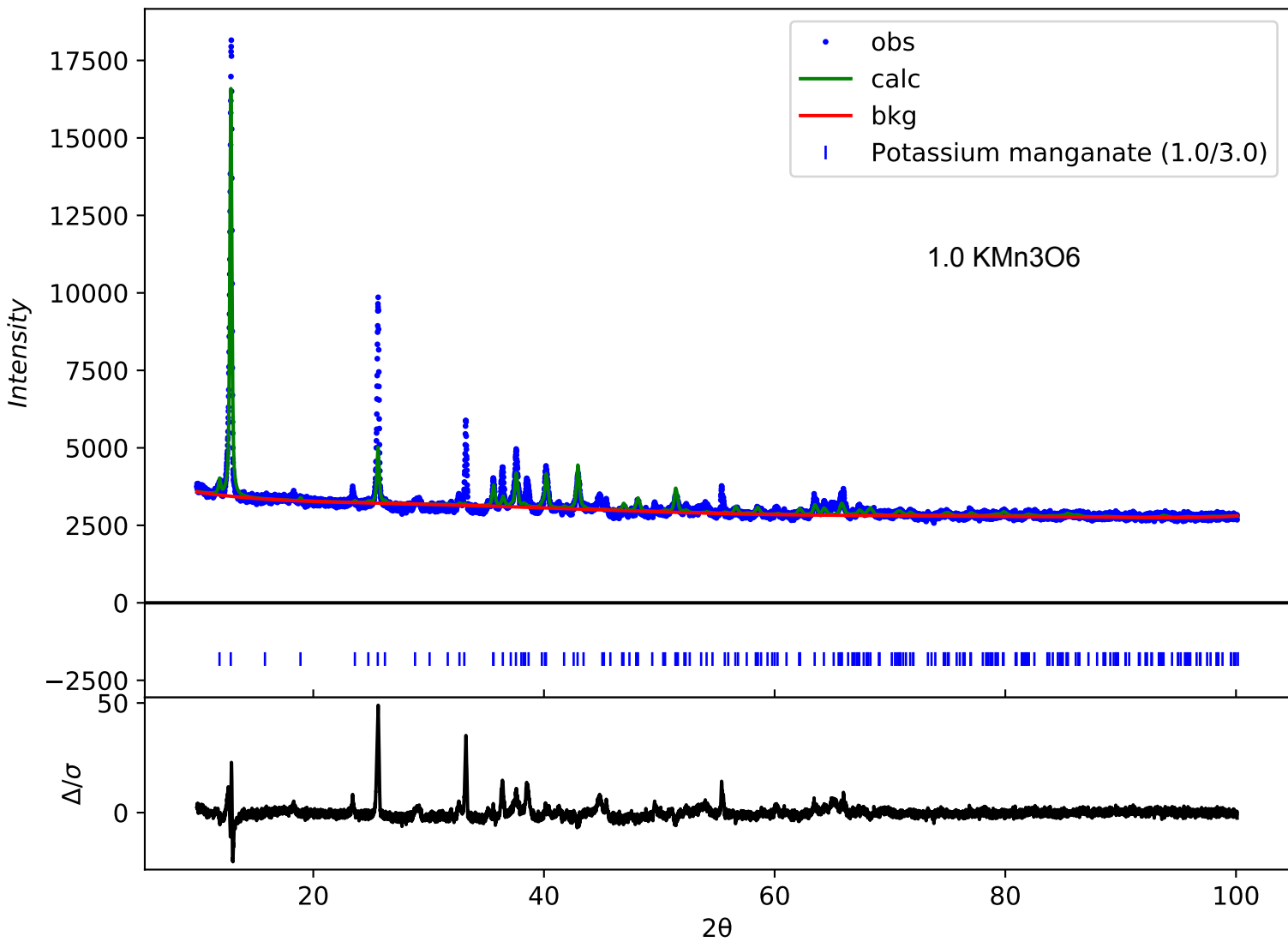

Supplement: Supplementary file 3 — This file contains the refined X-ray diffraction data from the successful syntheses performed by the A-Lab. The corresponding crystal structures used during refinement are also included in CIF format. [file 41586_2023_6734_MOESM3_ESM.zip › Automated_Refinement_Results/KMn3O6/KMn3O6_900_240_K2CO3_Mn2O3_recipe52_627f2040-ecac-45f0-9d52-7ade05f9da56.pdf]

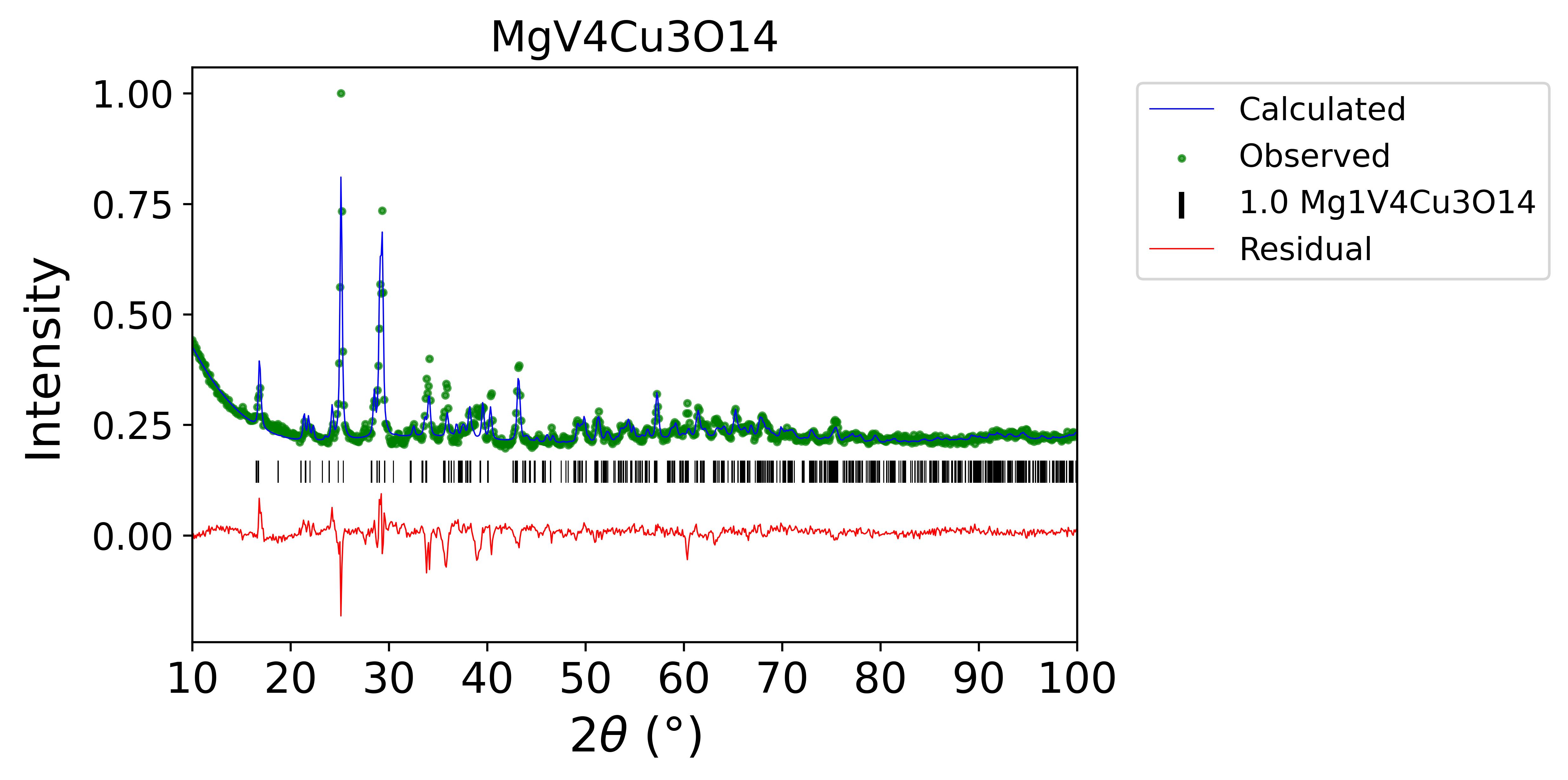

Supplement: Supplementary file 3 — This file contains the refined X-ray diffraction data from the successful syntheses performed by the A-Lab. The corresponding crystal structures used during refinement are also included in CIF format. [file 41586_2023_6734_MOESM3_ESM.zip › Automated_Refinement_Results/MgV4Cu3O14/MgV4Cu3O14_900_240_CuO_MgO_V2O5_recipe176_c5b0a061-8b5f-4ee3-a3dc-0e68d51370f1.jpg]

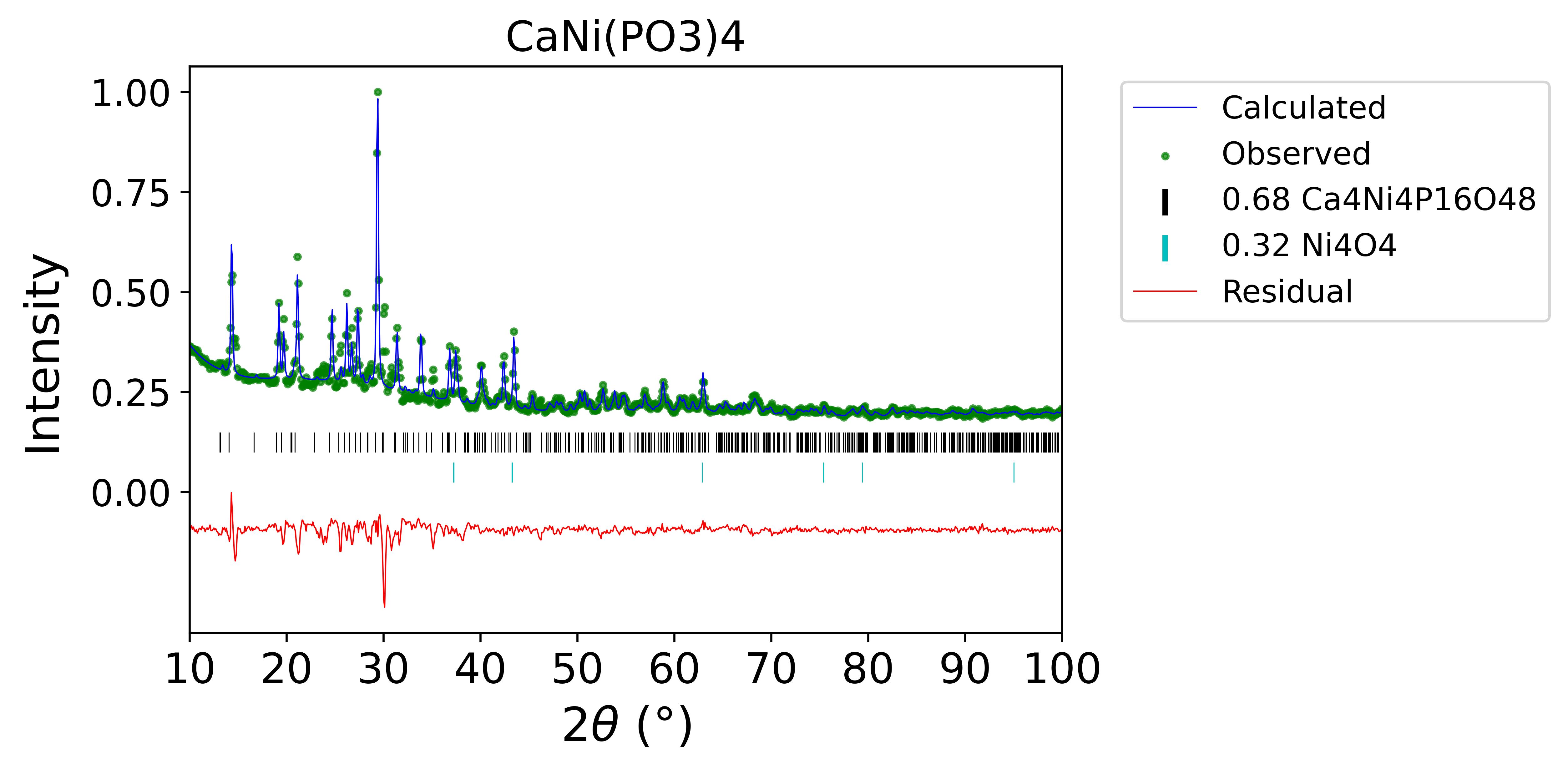

Supplement: Supplementary file 3 — This file contains the refined X-ray diffraction data from the successful syntheses performed by the A-Lab. The corresponding crystal structures used during refinement are also included in CIF format. [file 41586_2023_6734_MOESM3_ESM.zip › Automated_Refinement_Results/CaNi(PO3)4/CaNi(PO3)4_800_240_Ca(OH)2_(NH4)2HPO4_NiO_recipe23_e400579b-94a7-4638-b783-c95f4bfddaa4.jpg]

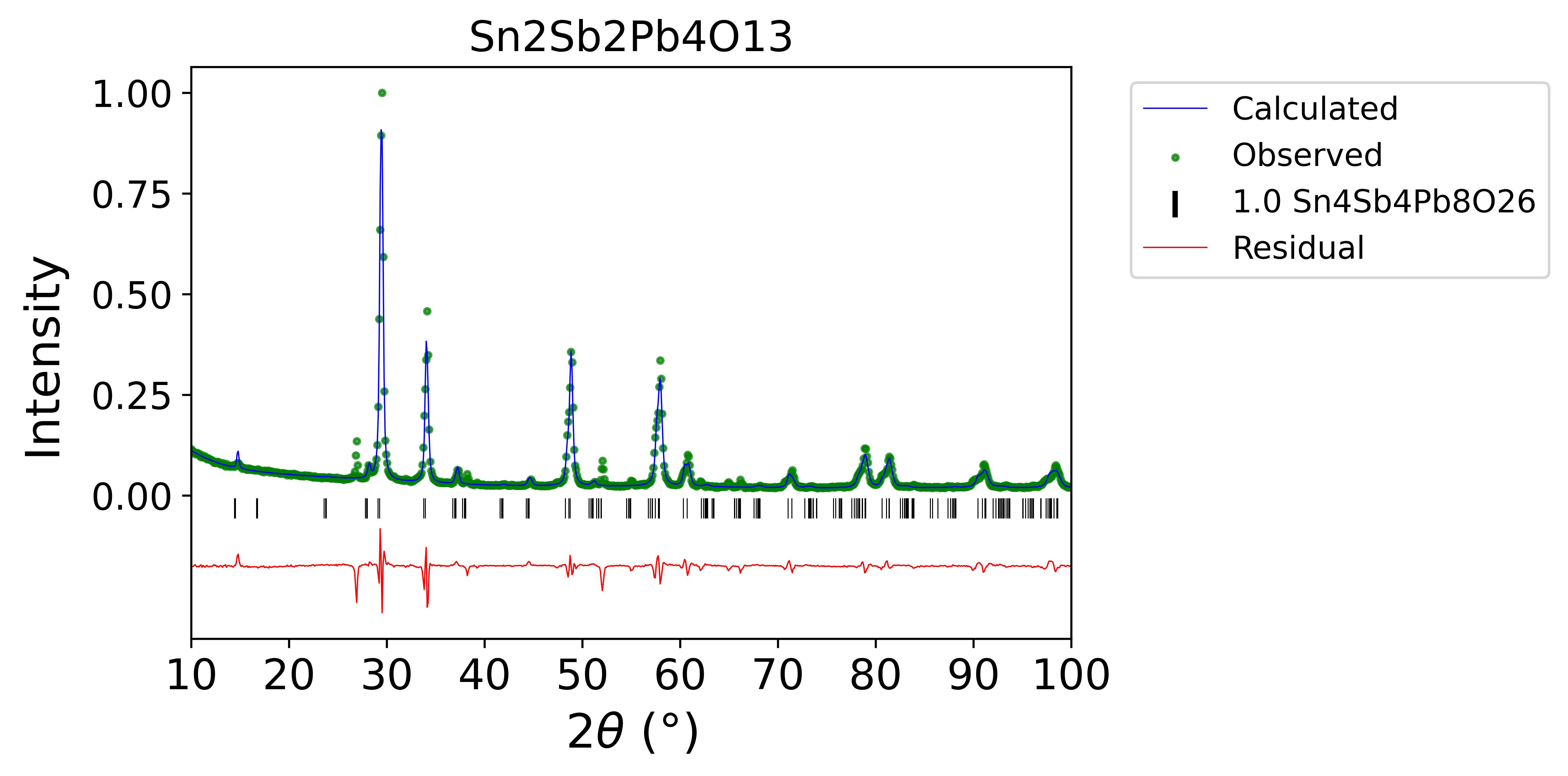

Supplement: Supplementary file 3 — This file contains the refined X-ray diffraction data from the successful syntheses performed by the A-Lab. The corresponding crystal structures used during refinement are also included in CIF format. [file 41586_2023_6734_MOESM3_ESM.zip › Automated_Refinement_Results/Sn2Sb2Pb4O13/Sn2Sb2Pb4O13_800_240_PbO_Sb2O3_SnO2_recipe253_aa9643eb-74e7-4fcd-86e5-0877ffb30c9d.jpg]

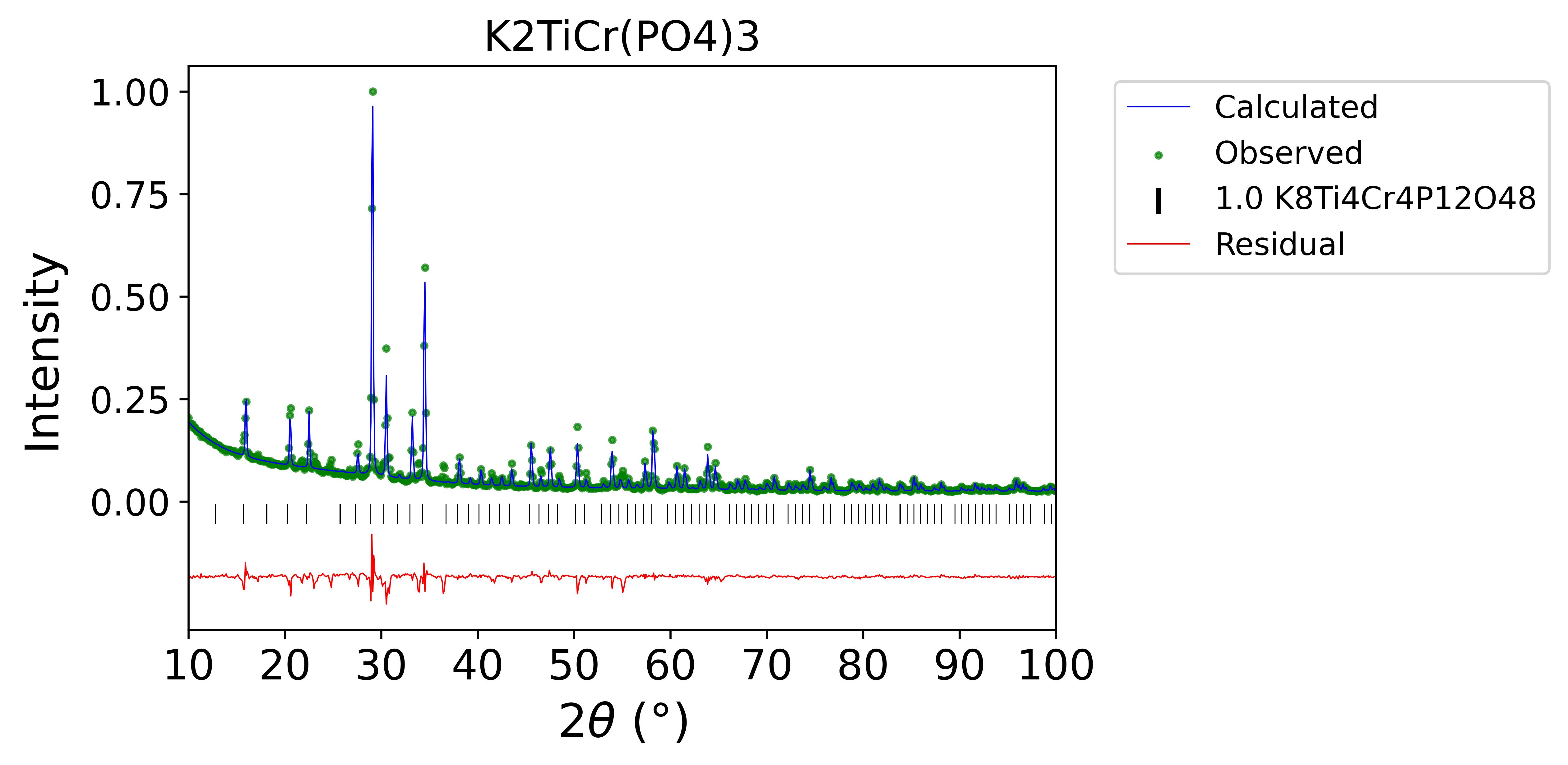

Supplement: Supplementary file 3 — This file contains the refined X-ray diffraction data from the successful syntheses performed by the A-Lab. The corresponding crystal structures used during refinement are also included in CIF format. [file 41586_2023_6734_MOESM3_ESM.zip › Automated_Refinement_Results/K2TiCr(PO4)3/K2TiCr(PO4)3_1100_240_Cr2O3_K2CO3_(NH4)2HPO4_TiO2_recipe185_474922ab-90e6-43b1-896b-9924506535bb.jpg]

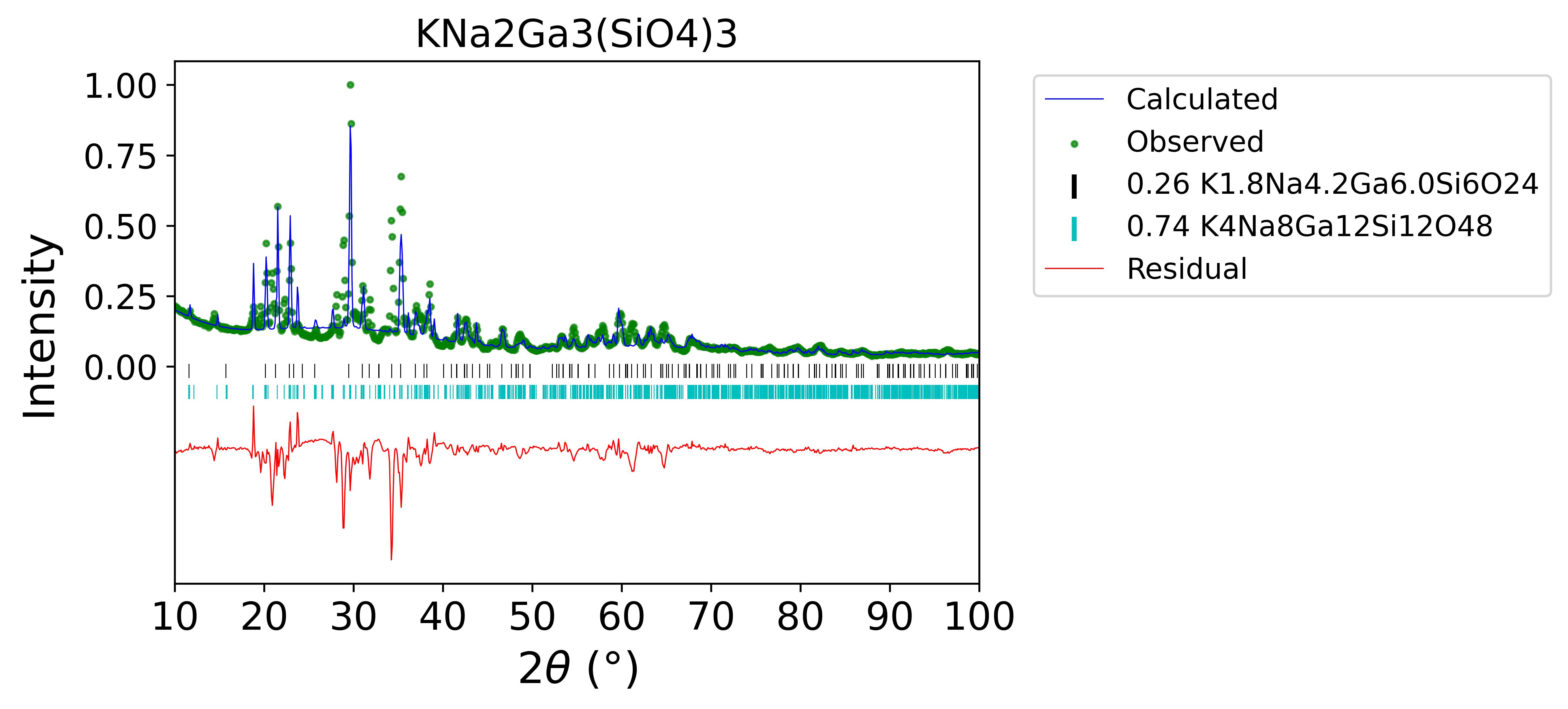

Supplement: Supplementary file 3 — This file contains the refined X-ray diffraction data from the successful syntheses performed by the A-Lab. The corresponding crystal structures used during refinement are also included in CIF format. [file 41586_2023_6734_MOESM3_ESM.zip › Automated_Refinement_Results/KNa2Ga3(SiO4)3/KNa2Ga3(SiO4)3_900_240_Ga2O3_K2CO3_Na2CO3_SiO2_ARRrecipe163_ead97622-f581-411c-a3ab-bea62d9e9004.jpg]

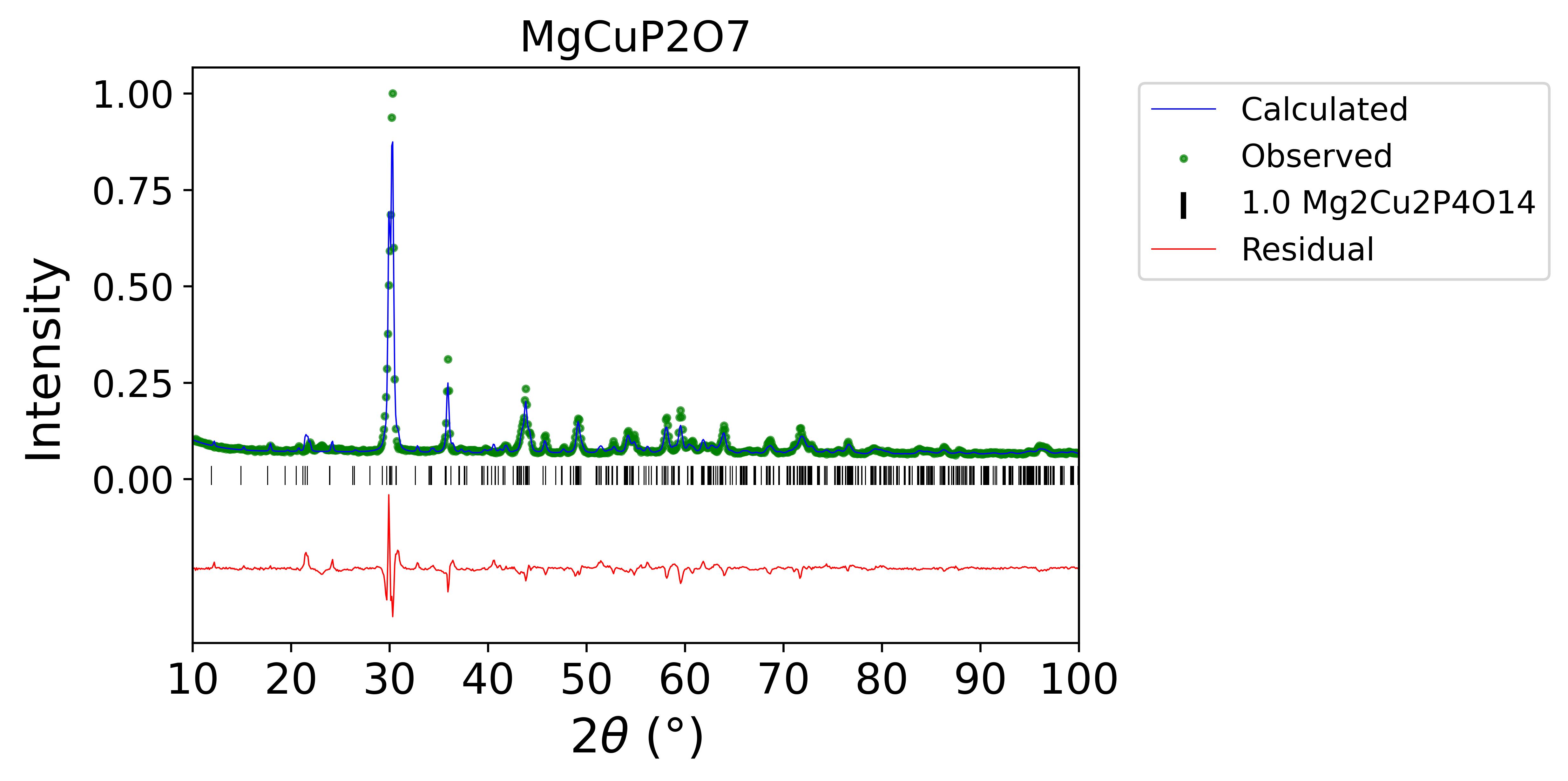

Supplement: Supplementary file 3 — This file contains the refined X-ray diffraction data from the successful syntheses performed by the A-Lab. The corresponding crystal structures used during refinement are also included in CIF format. [file 41586_2023_6734_MOESM3_ESM.zip › Automated_Refinement_Results/MgCuP2O7/MgCuP2O7_1000_240_CuO_MgO_NH4H2PO4_recipe190_37f43fef-9737-4e62-8678-100ea4e25fdd.jpg]

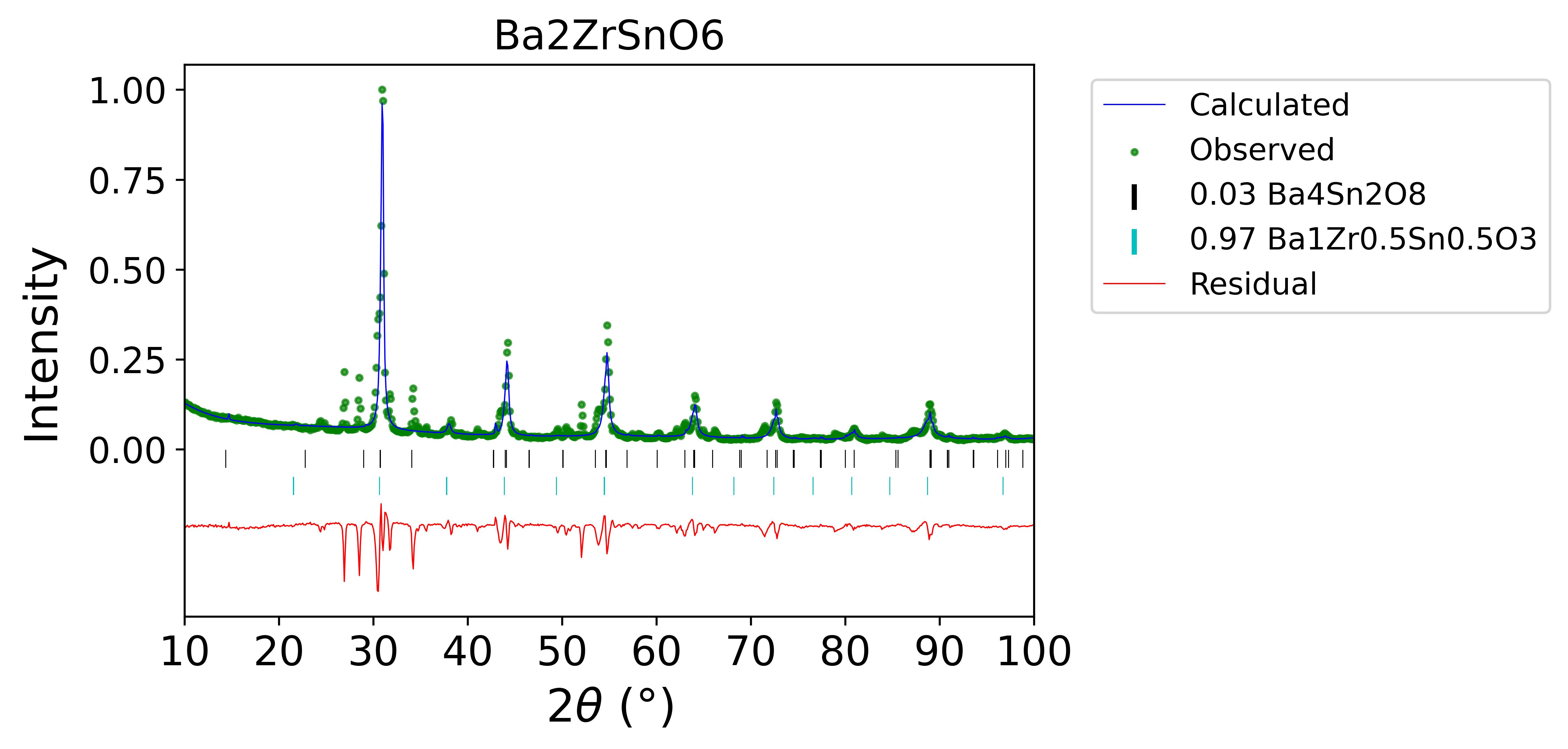

Supplement: Supplementary file 3 — This file contains the refined X-ray diffraction data from the successful syntheses performed by the A-Lab. The corresponding crystal structures used during refinement are also included in CIF format. [file 41586_2023_6734_MOESM3_ESM.zip › Automated_Refinement_Results/Ba2ZrSnO6/Ba2ZrSnO6_1000_240_Ba(OH)2_SnO2_ZrO2_recipe154_751fb7a7-d8f8-4552-a6d8-c8115dfda3e5.jpg]

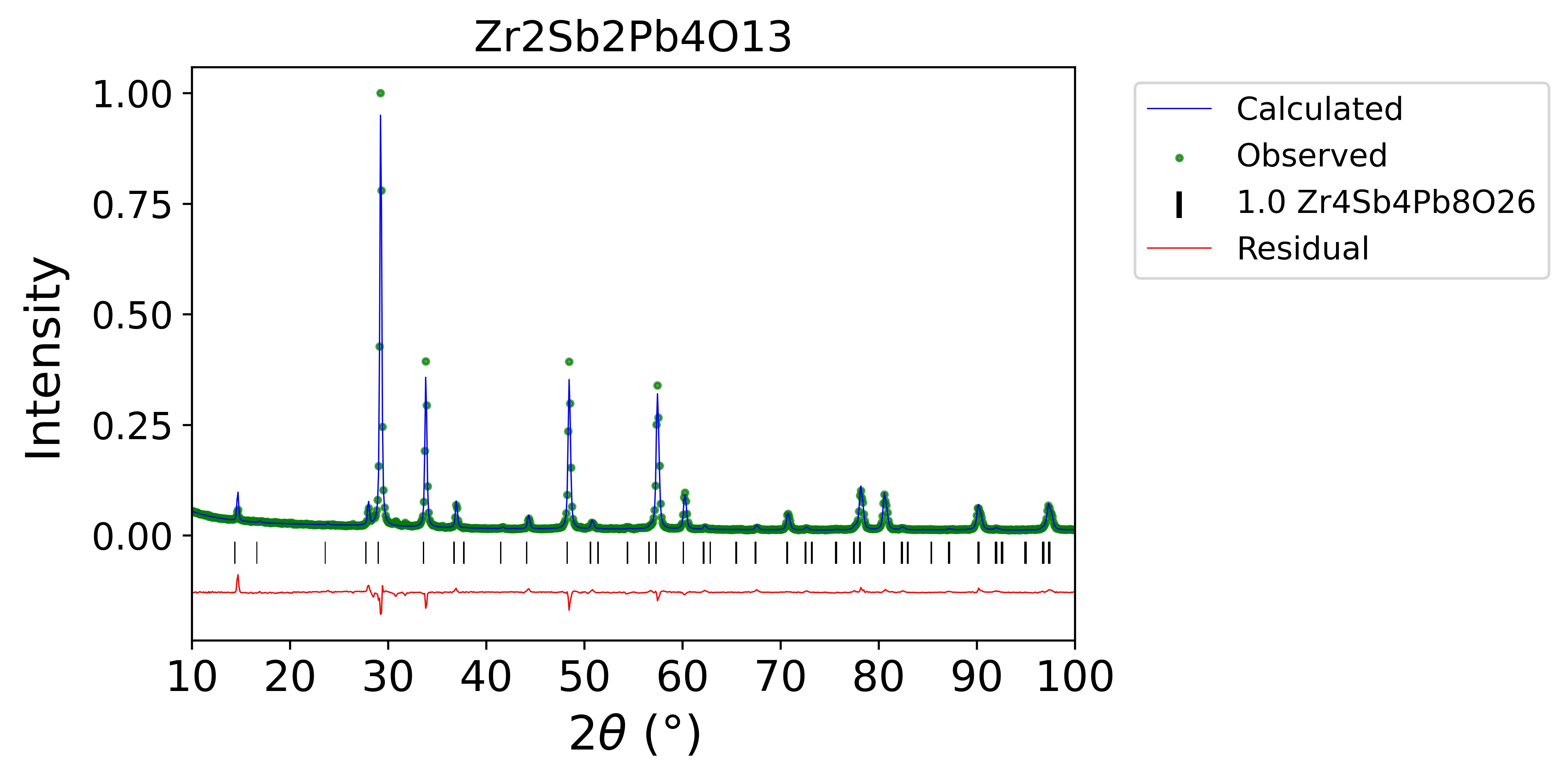

Supplement: Supplementary file 3 — This file contains the refined X-ray diffraction data from the successful syntheses performed by the A-Lab. The corresponding crystal structures used during refinement are also included in CIF format. [file 41586_2023_6734_MOESM3_ESM.zip › Automated_Refinement_Results/Zr2Sb2Pb4O13/Zr2Sb2Pb4O13_900_240_PbCO3_Sb2O3_ZrO2_recipe157_ecdf6894-d168-4ad3-9ba5-ffe23945927e.jpg]

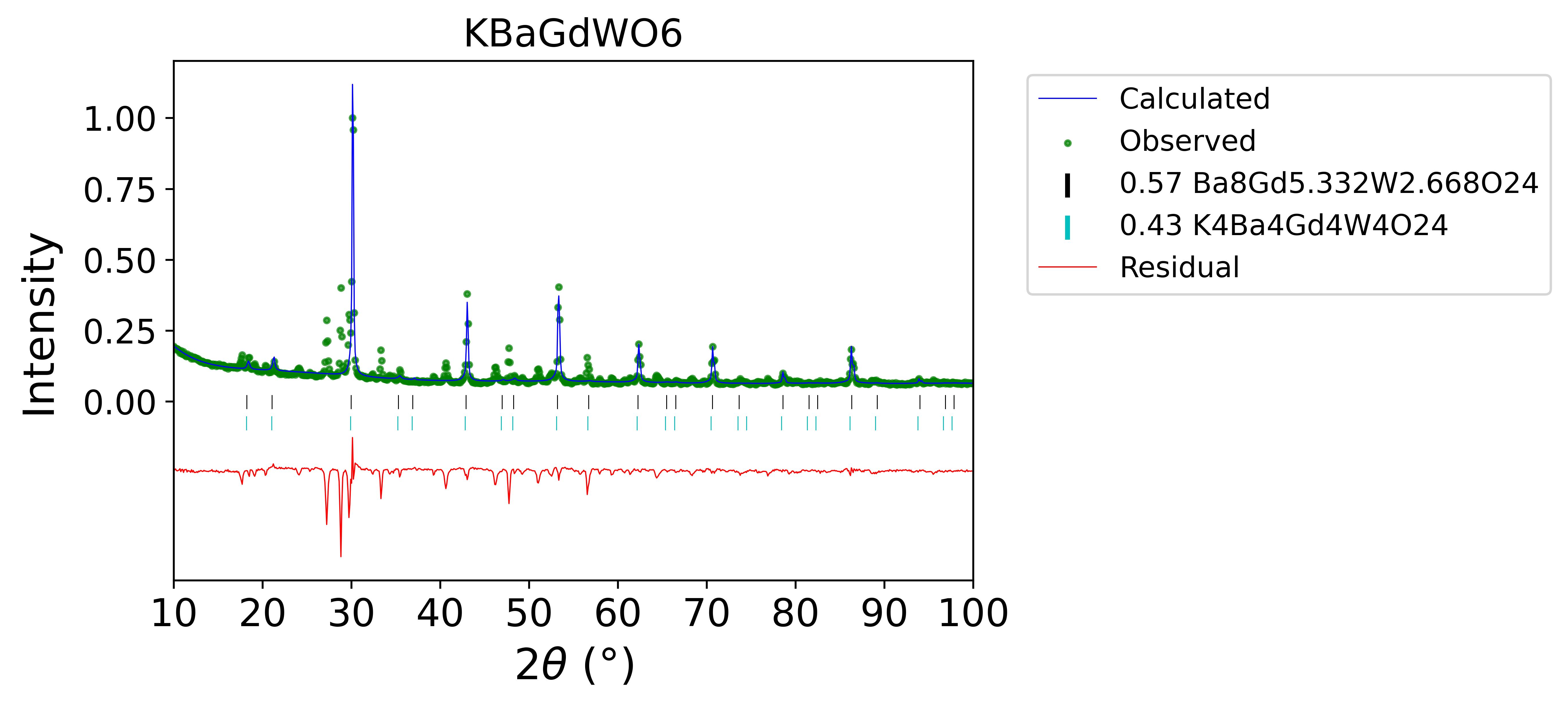

Supplement: Supplementary file 3 — This file contains the refined X-ray diffraction data from the successful syntheses performed by the A-Lab. The corresponding crystal structures used during refinement are also included in CIF format. [file 41586_2023_6734_MOESM3_ESM.zip › Automated_Refinement_Results/KBaGdWO6/KBaGdWO6_1000_240_BaO2_Gd2O3_K2CO3_WO3_recipe263_ad61ea87-d21e-494a-8f6e-9b371fd1dd9d.jpg]

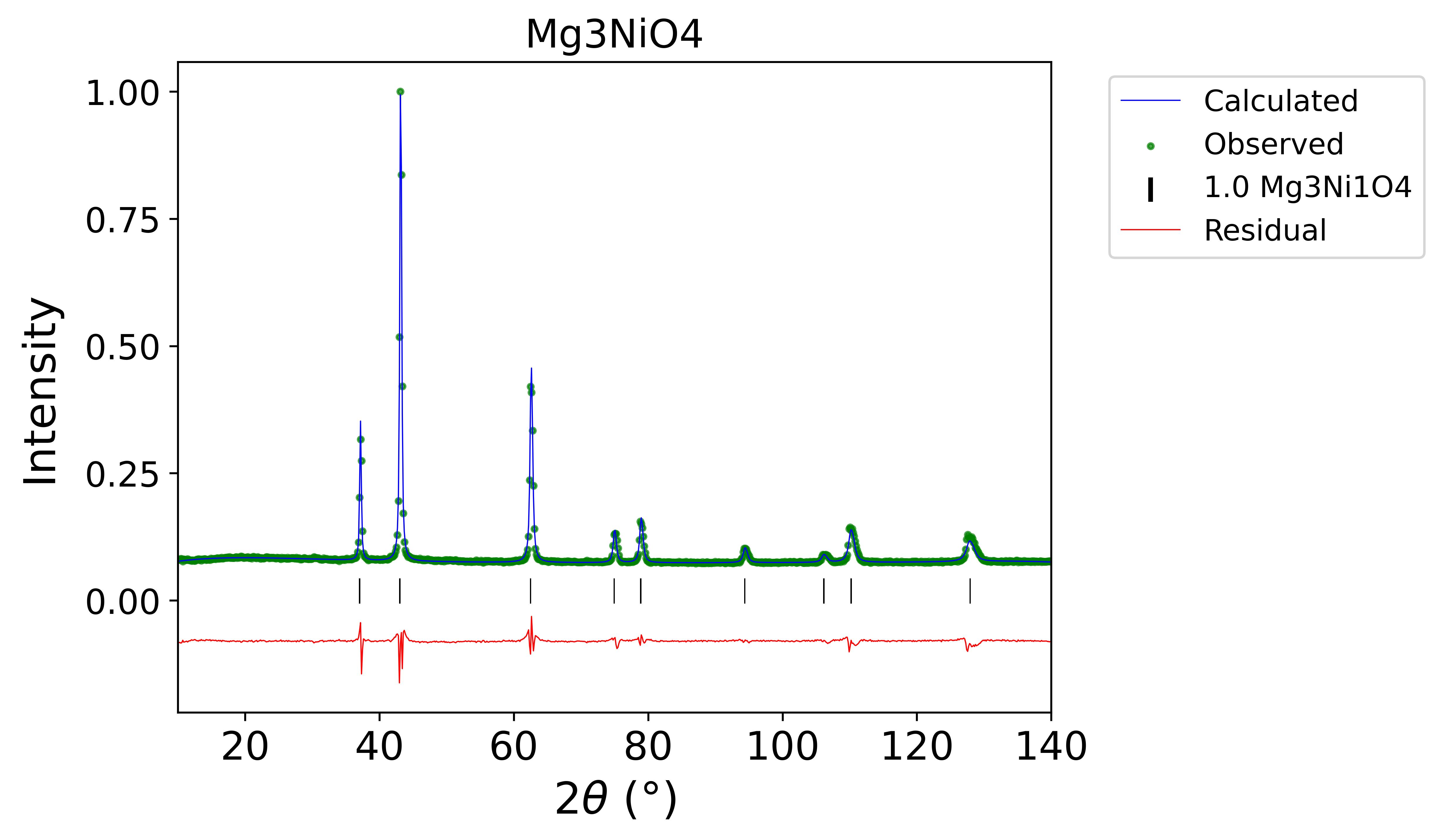

Supplement: Supplementary file 3 — This file contains the refined X-ray diffraction data from the successful syntheses performed by the A-Lab. The corresponding crystal structures used during refinement are also included in CIF format. [file 41586_2023_6734_MOESM3_ESM.zip › Automated_Refinement_Results/Mg3NiO4/Mg3NiO4_1000_240_MgO_NiO_MPReheatrecipe6_reheat-manual-pellet-mixing_structure_1_updated.jpg]

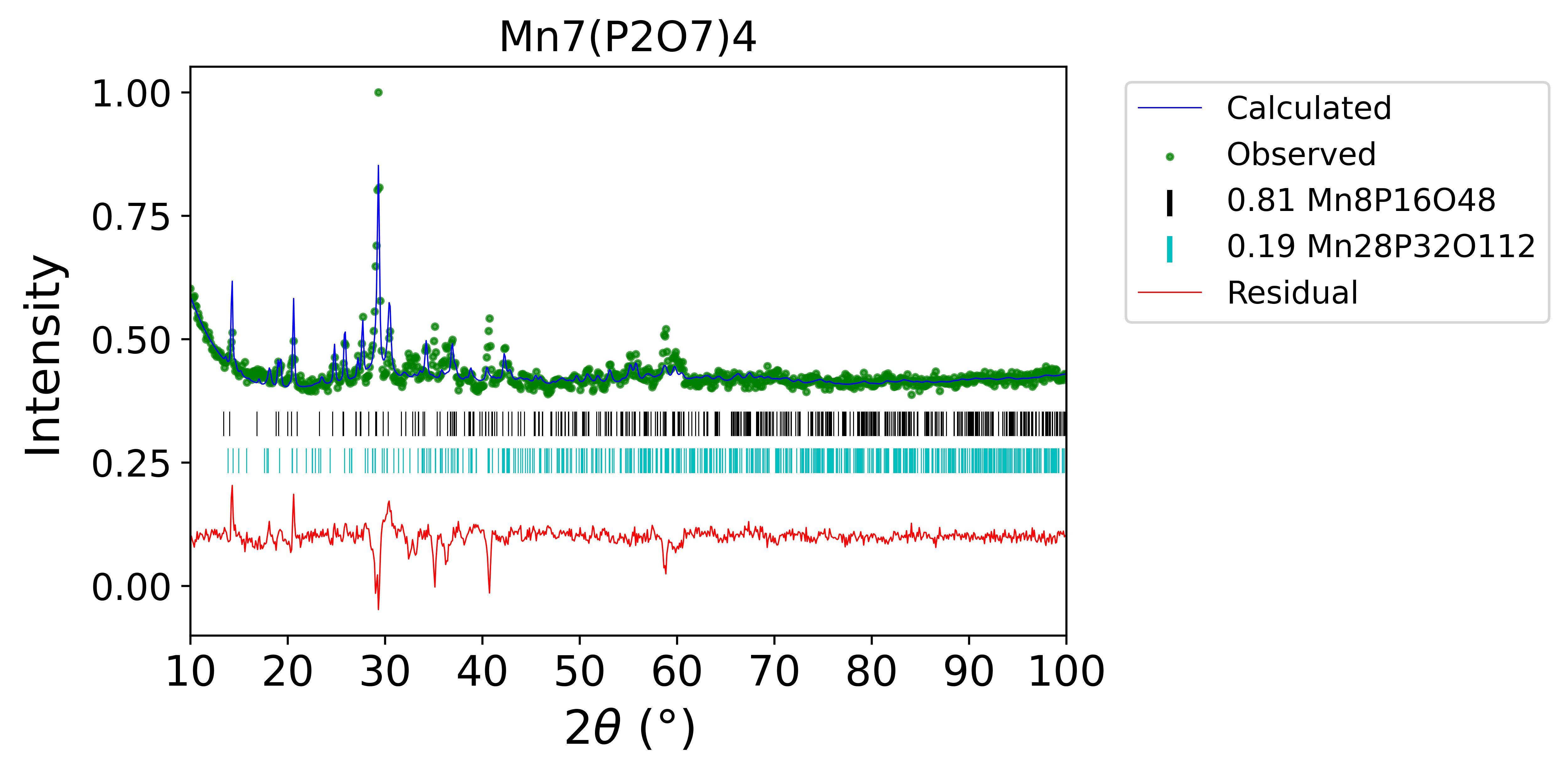

Supplement: Supplementary file 3 — This file contains the refined X-ray diffraction data from the successful syntheses performed by the A-Lab. The corresponding crystal structures used during refinement are also included in CIF format. [file 41586_2023_6734_MOESM3_ESM.zip › Automated_Refinement_Results/Mn7(P2O7)4/Mn7(P2O7)4_500_240_NH4H2PO4_MnO_recipe19_2875c26b-7134-415a-93d6-e664b871c17b.jpg]

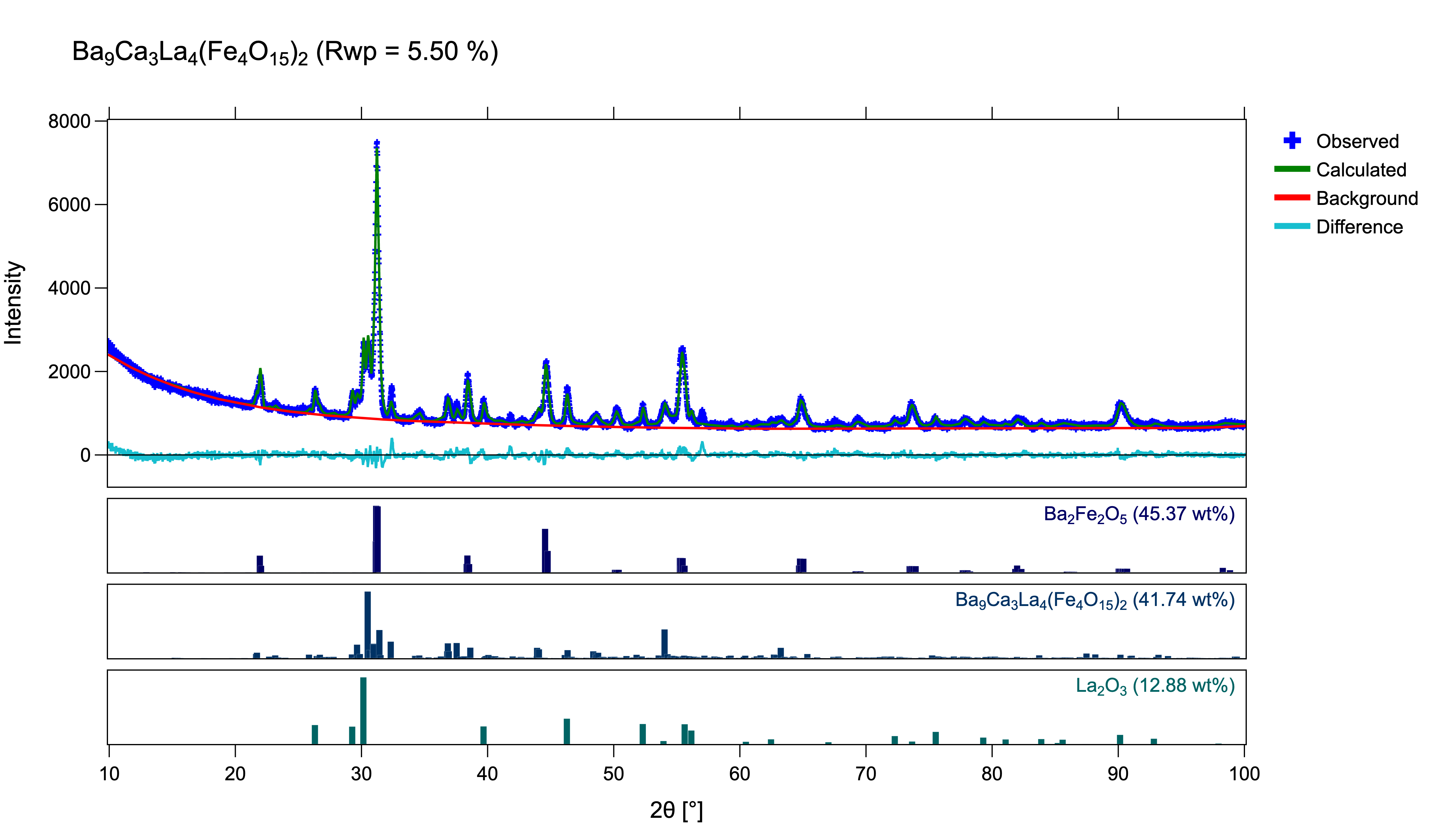

Supplement: Supplementary file 3 — This file contains the refined X-ray diffraction data from the successful syntheses performed by the A-Lab. The corresponding crystal structures used during refinement are also included in CIF format. [file 41586_2023_6734_MOESM3_ESM.zip › Manual_Refinement_Results/Ba9Ca3La4(Fe4O15)2/Ba9Ca3La4(Fe4O15)2.png]

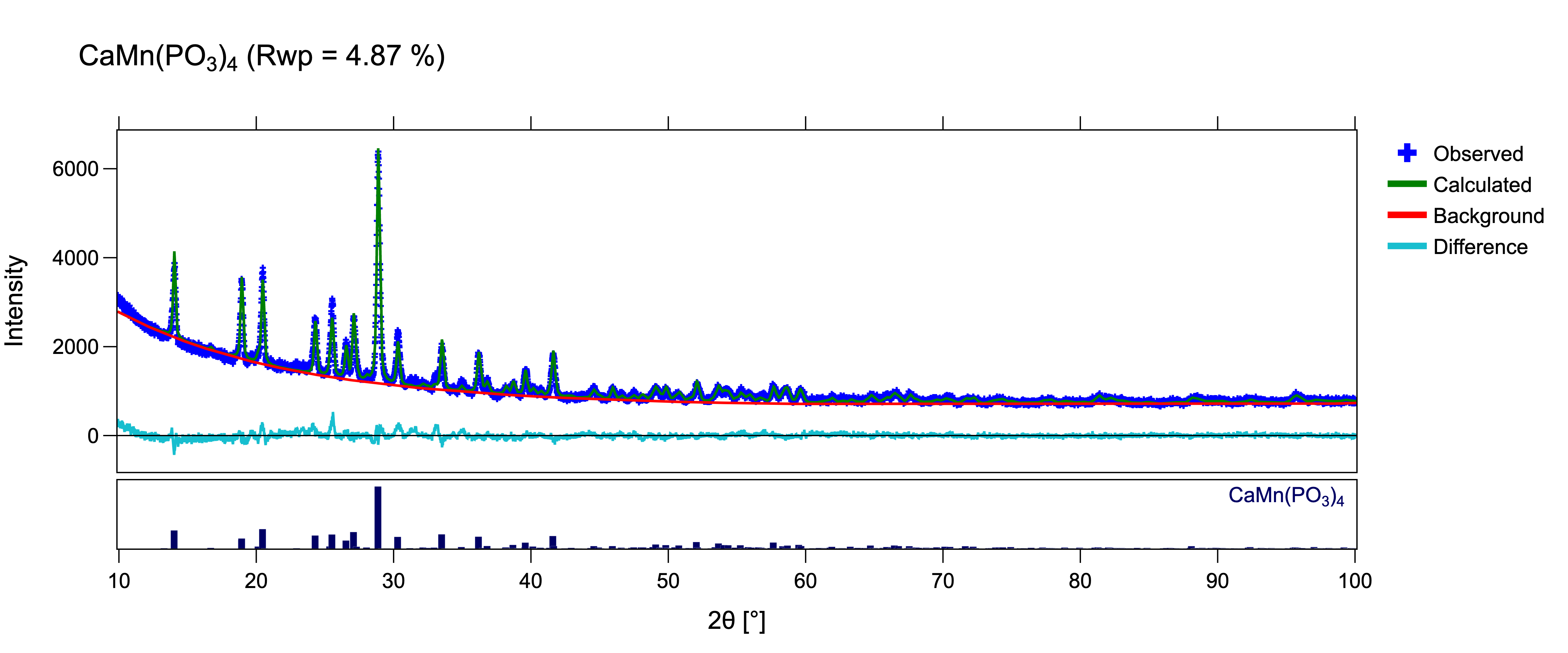

Supplement: Supplementary file 3 — This file contains the refined X-ray diffraction data from the successful syntheses performed by the A-Lab. The corresponding crystal structures used during refinement are also included in CIF format. [file 41586_2023_6734_MOESM3_ESM.zip › Manual_Refinement_Results/CaMn(PO3)4/CaMn(PO3)4.png]

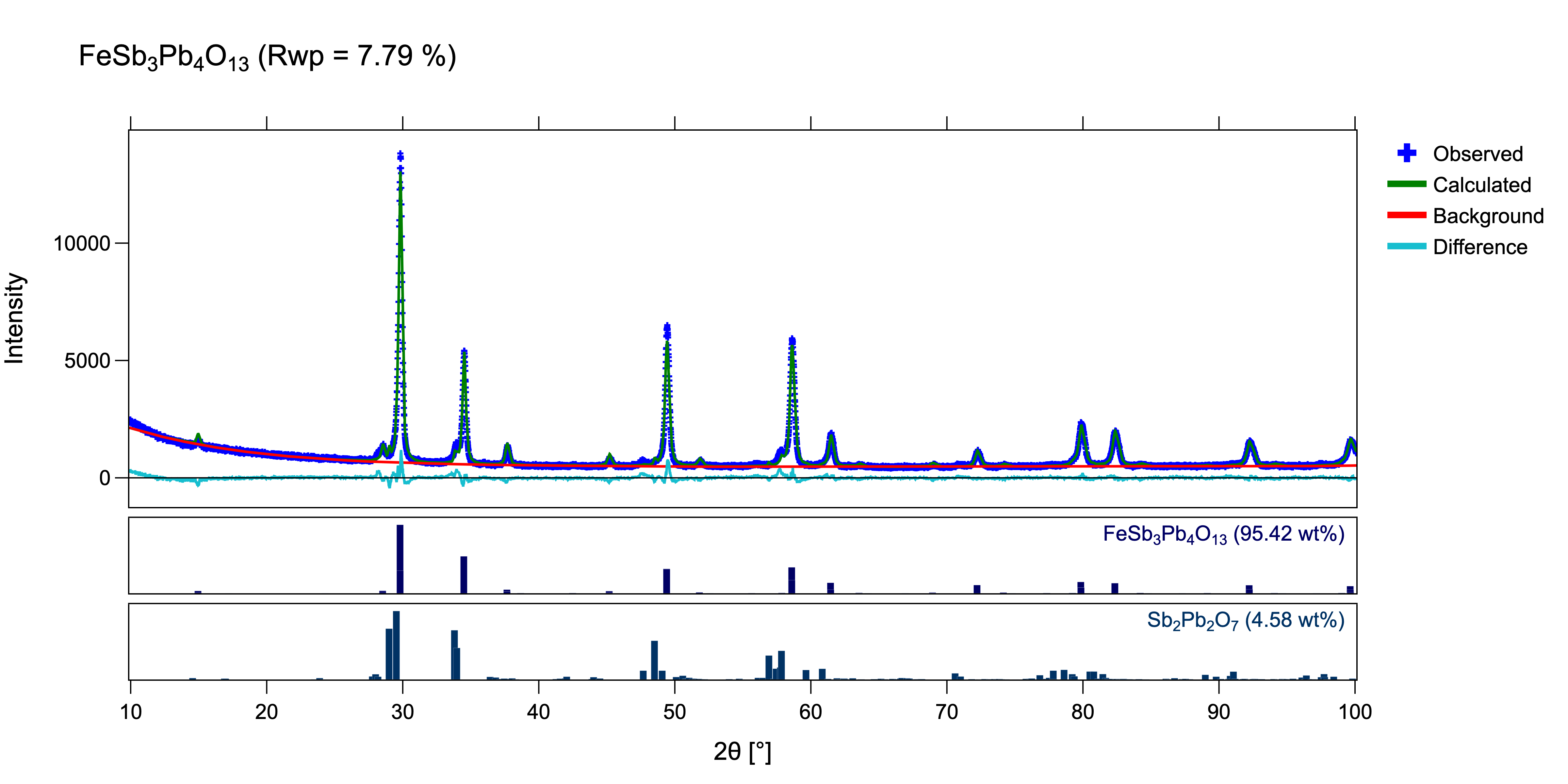

Supplement: Supplementary file 3 — This file contains the refined X-ray diffraction data from the successful syntheses performed by the A-Lab. The corresponding crystal structures used during refinement are also included in CIF format. [file 41586_2023_6734_MOESM3_ESM.zip › Manual_Refinement_Results/FeSb3Pb4O13/FeSb3Pb4O13.png]

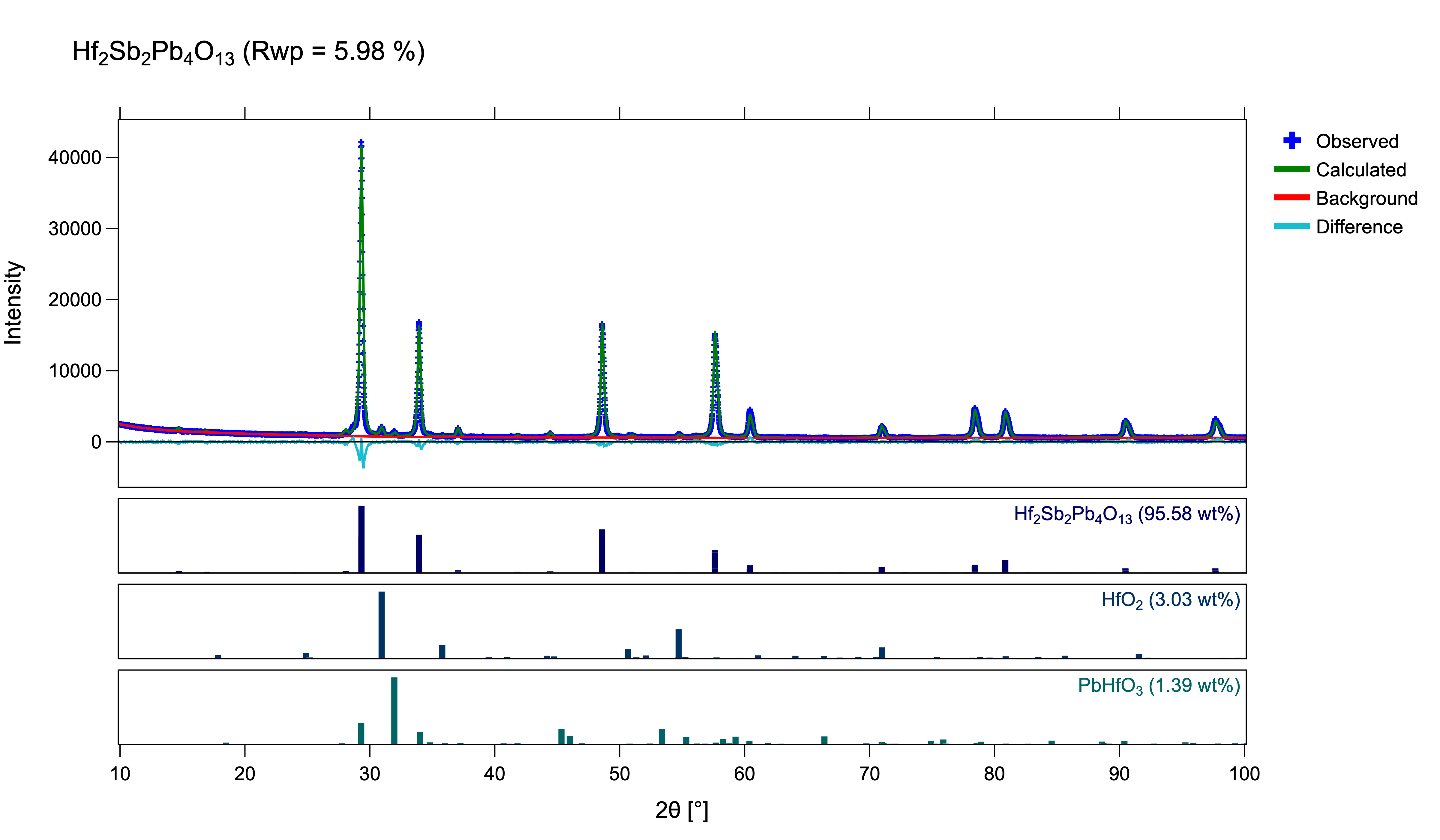

Supplement: Supplementary file 3 — This file contains the refined X-ray diffraction data from the successful syntheses performed by the A-Lab. The corresponding crystal structures used during refinement are also included in CIF format. [file 41586_2023_6734_MOESM3_ESM.zip › Manual_Refinement_Results/Hf2Sb2Pb4O13/Hf2Sb2Pb4O13.png]

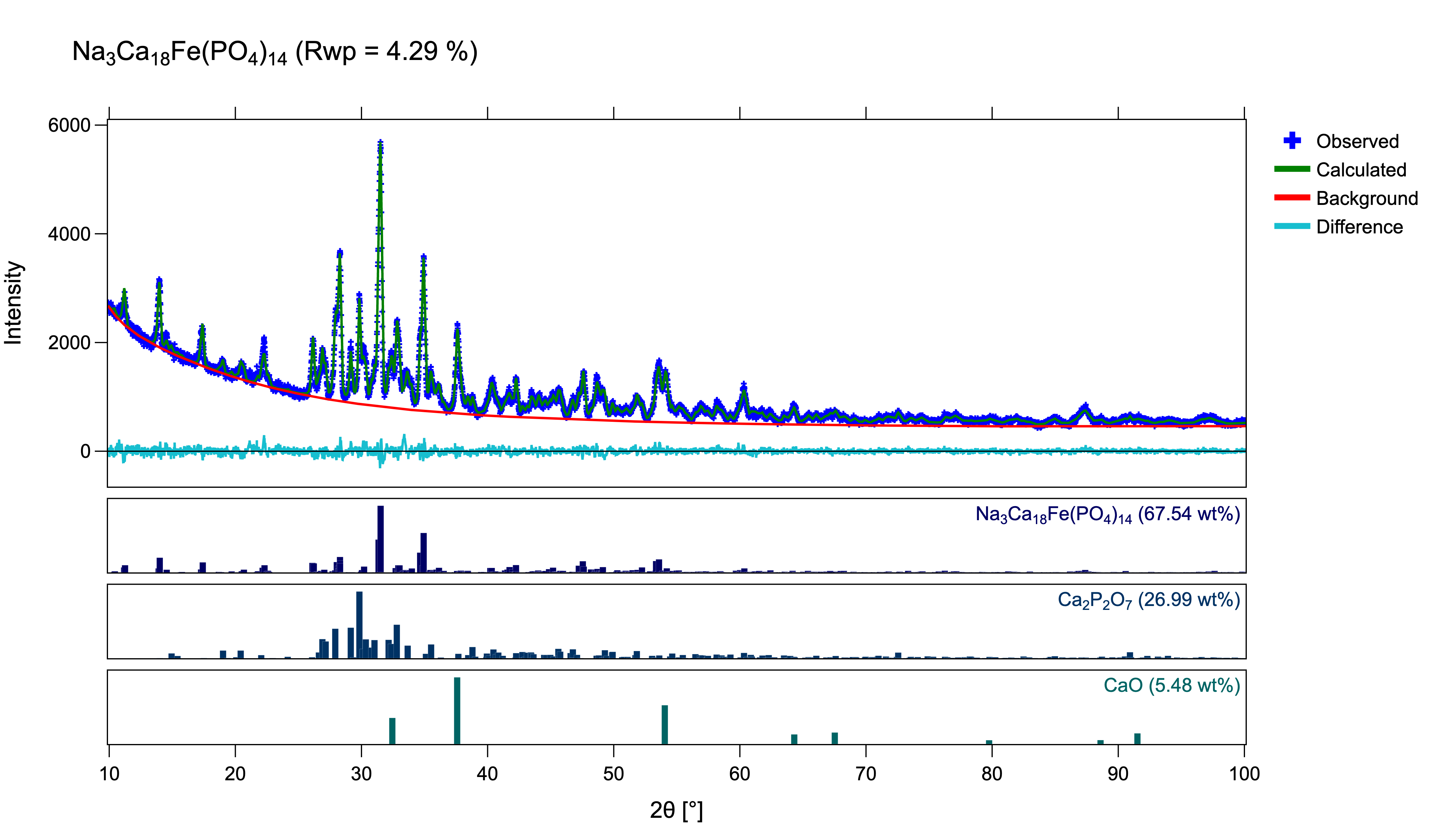

Supplement: Supplementary file 3 — This file contains the refined X-ray diffraction data from the successful syntheses performed by the A-Lab. The corresponding crystal structures used during refinement are also included in CIF format. [file 41586_2023_6734_MOESM3_ESM.zip › Manual_Refinement_Results/Na3Ca18Fe(PO4)14/Na3Ca18Fe(PO4)14.png]

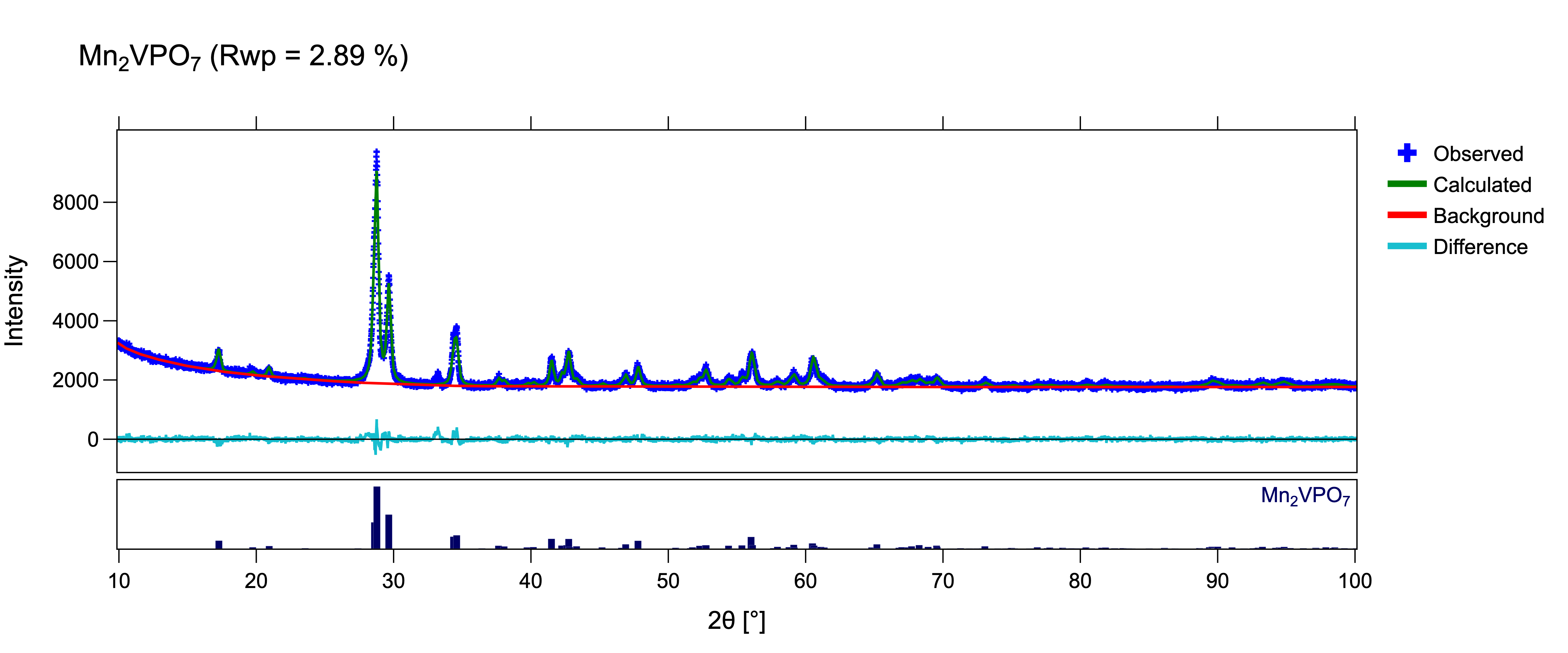

Supplement: Supplementary file 3 — This file contains the refined X-ray diffraction data from the successful syntheses performed by the A-Lab. The corresponding crystal structures used during refinement are also included in CIF format. [file 41586_2023_6734_MOESM3_ESM.zip › Manual_Refinement_Results/Mn2VPO7/Mn2VPO7.png]

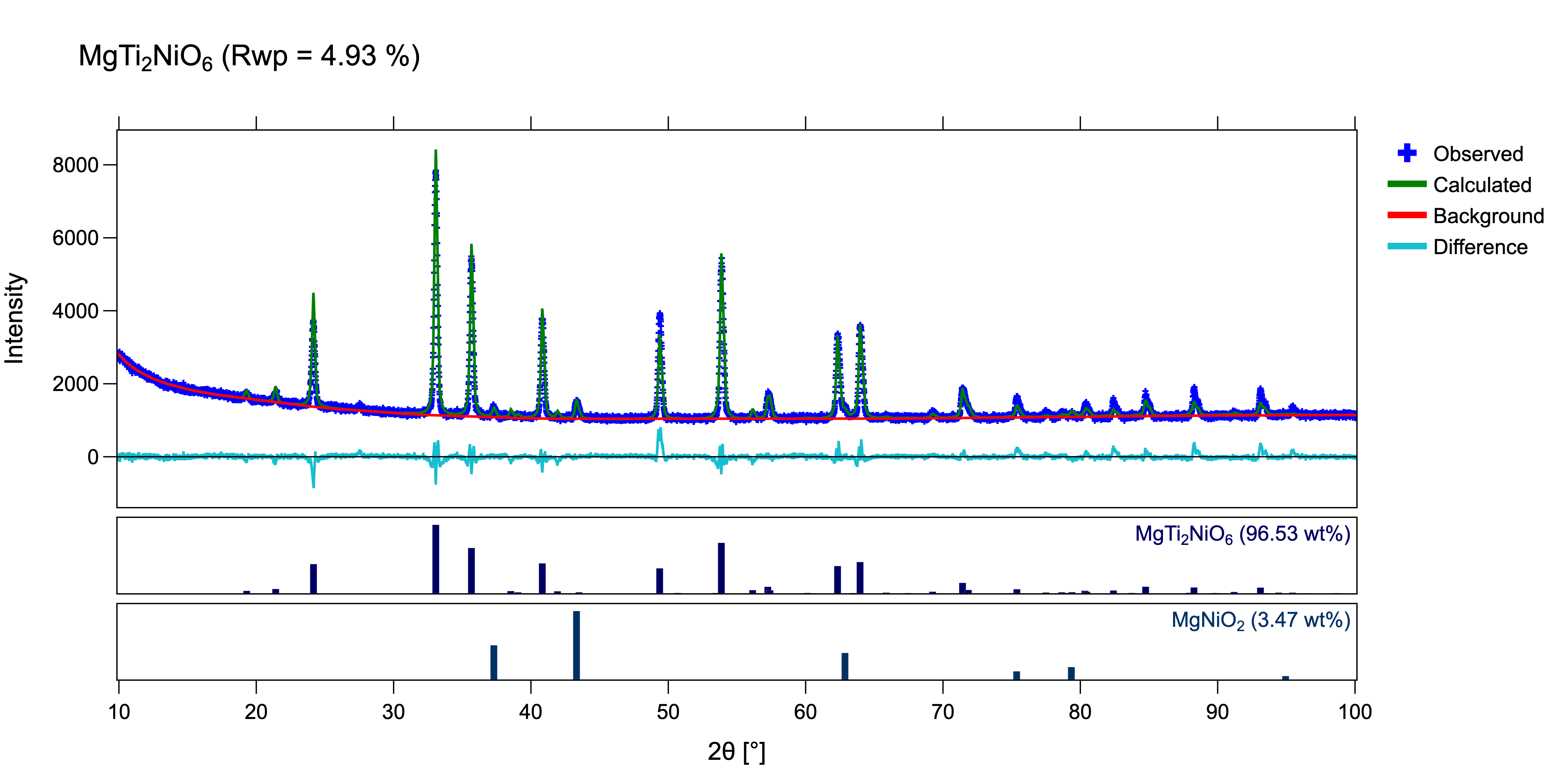

Supplement: Supplementary file 3 — This file contains the refined X-ray diffraction data from the successful syntheses performed by the A-Lab. The corresponding crystal structures used during refinement are also included in CIF format. [file 41586_2023_6734_MOESM3_ESM.zip › Manual_Refinement_Results/MgTi2NiO6/MgTi2NiO6.png]

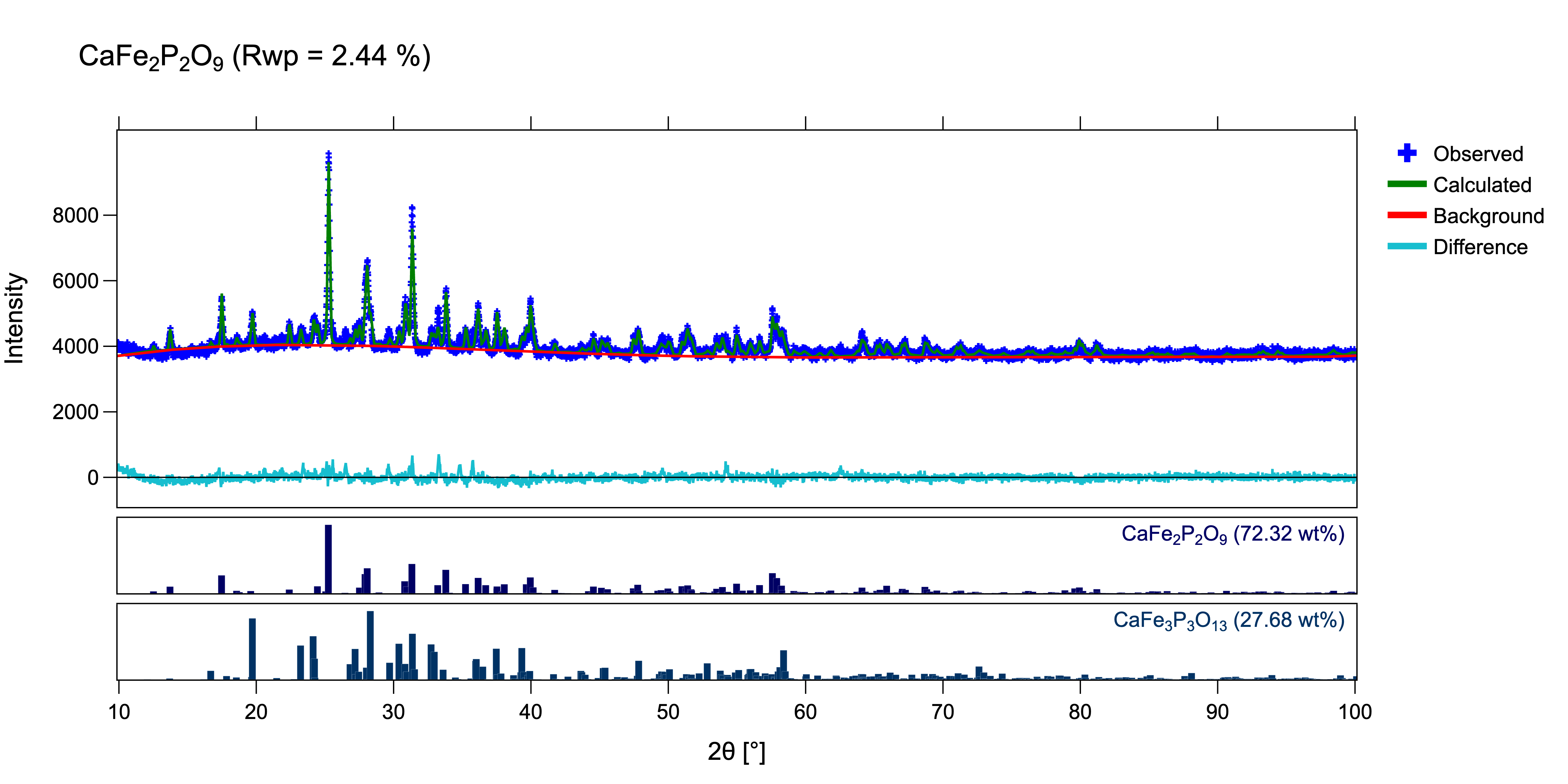

Supplement: Supplementary file 3 — This file contains the refined X-ray diffraction data from the successful syntheses performed by the A-Lab. The corresponding crystal structures used during refinement are also included in CIF format. [file 41586_2023_6734_MOESM3_ESM.zip › Manual_Refinement_Results/CaFe2P2O9/CaFe2P2O9.png]

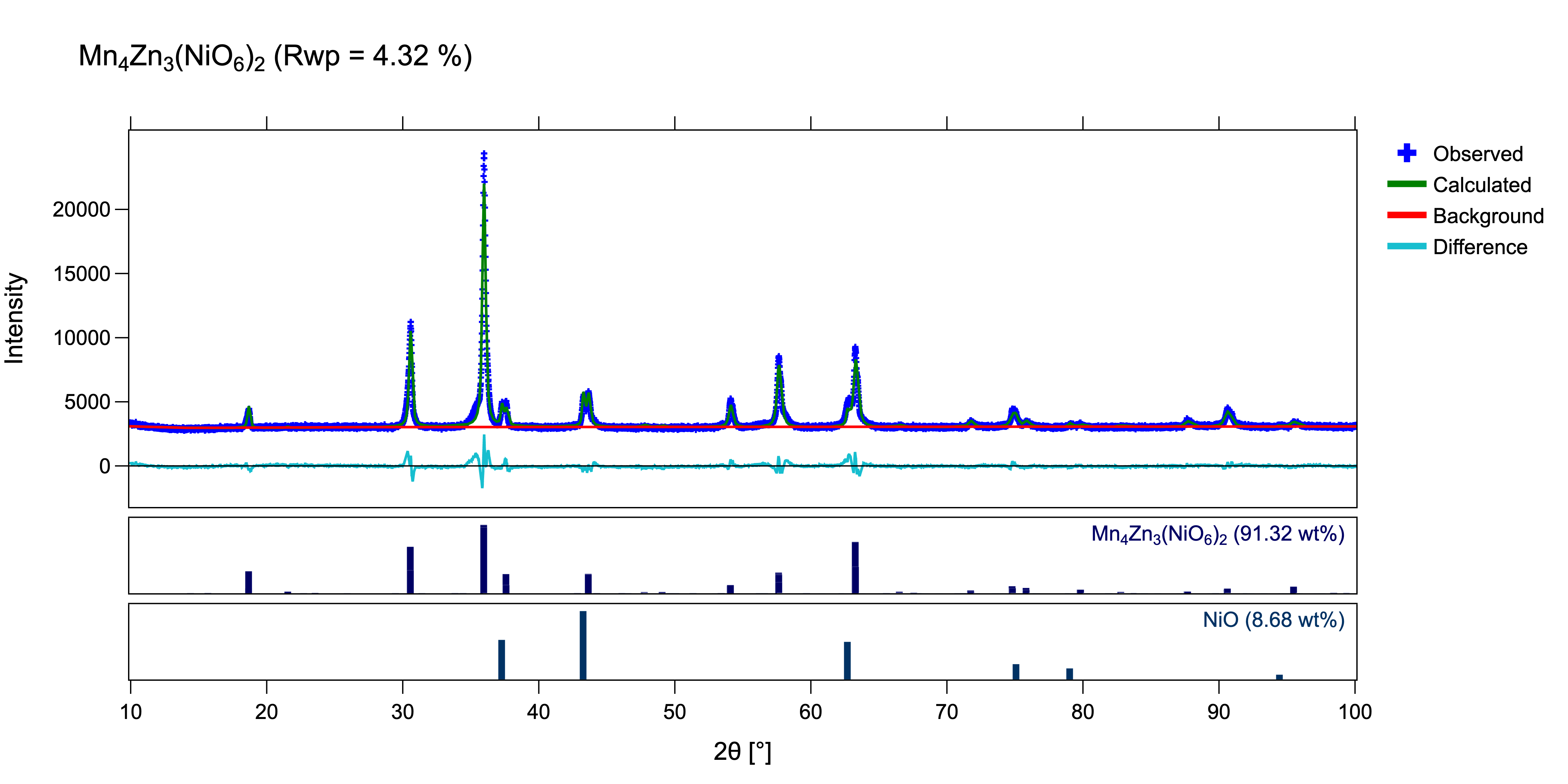

Supplement: Supplementary file 3 — This file contains the refined X-ray diffraction data from the successful syntheses performed by the A-Lab. The corresponding crystal structures used during refinement are also included in CIF format. [file 41586_2023_6734_MOESM3_ESM.zip › Manual_Refinement_Results/Mn4Zn3(NiO6)2/Mn4Zn3(NiO6)2.png]

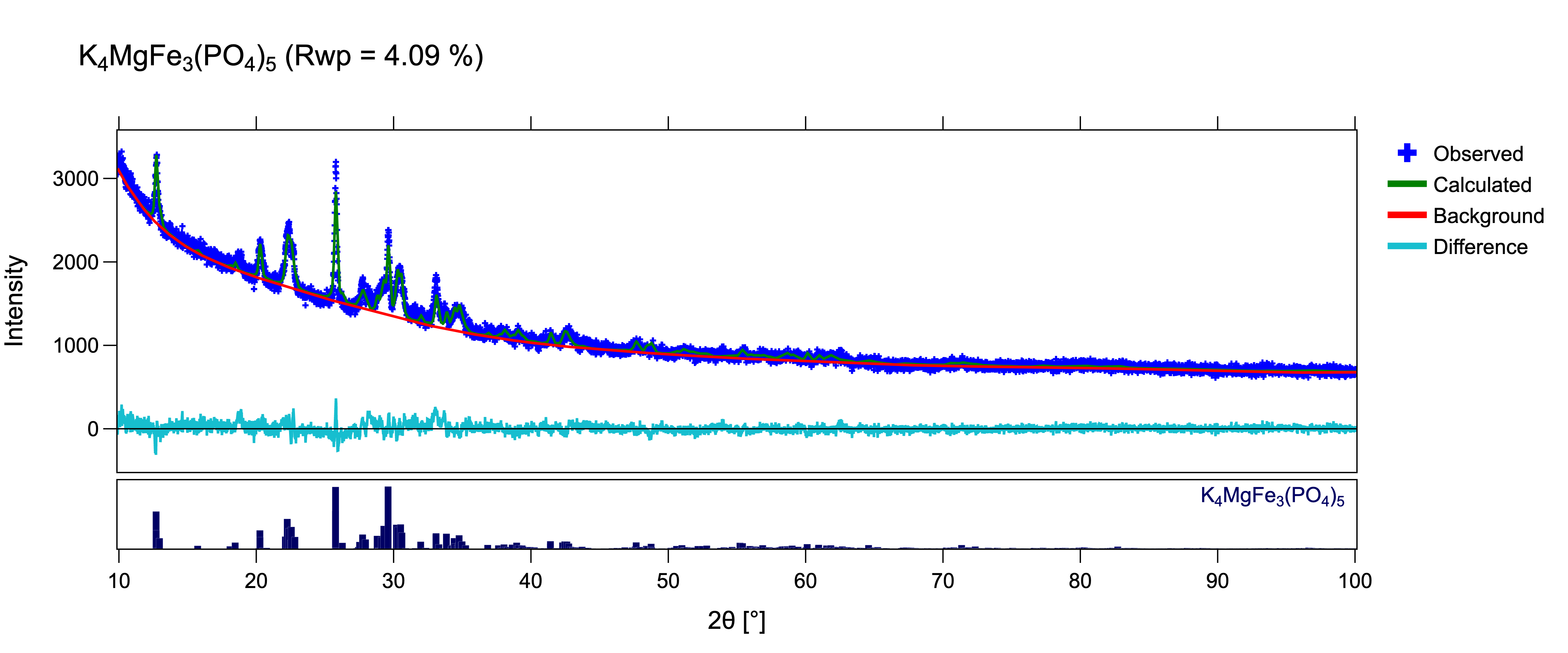

Supplement: Supplementary file 3 — This file contains the refined X-ray diffraction data from the successful syntheses performed by the A-Lab. The corresponding crystal structures used during refinement are also included in CIF format. [file 41586_2023_6734_MOESM3_ESM.zip › Manual_Refinement_Results/K4MgFe3(PO4)5/K4MgFe3(PO4)5.png]

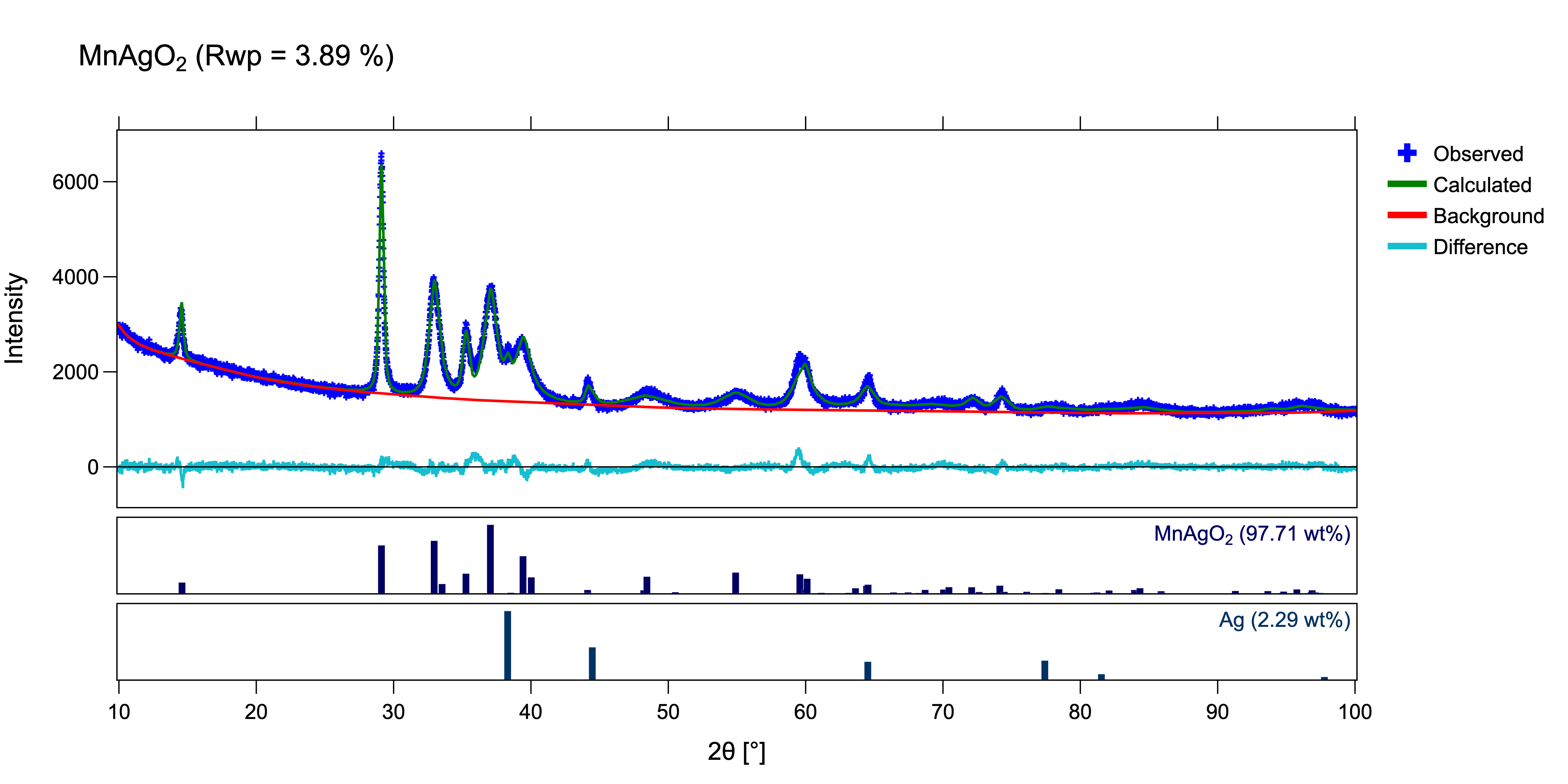

Supplement: Supplementary file 3 — This file contains the refined X-ray diffraction data from the successful syntheses performed by the A-Lab. The corresponding crystal structures used during refinement are also included in CIF format. [file 41586_2023_6734_MOESM3_ESM.zip › Manual_Refinement_Results/MnAgO2/MnAgO2.png]

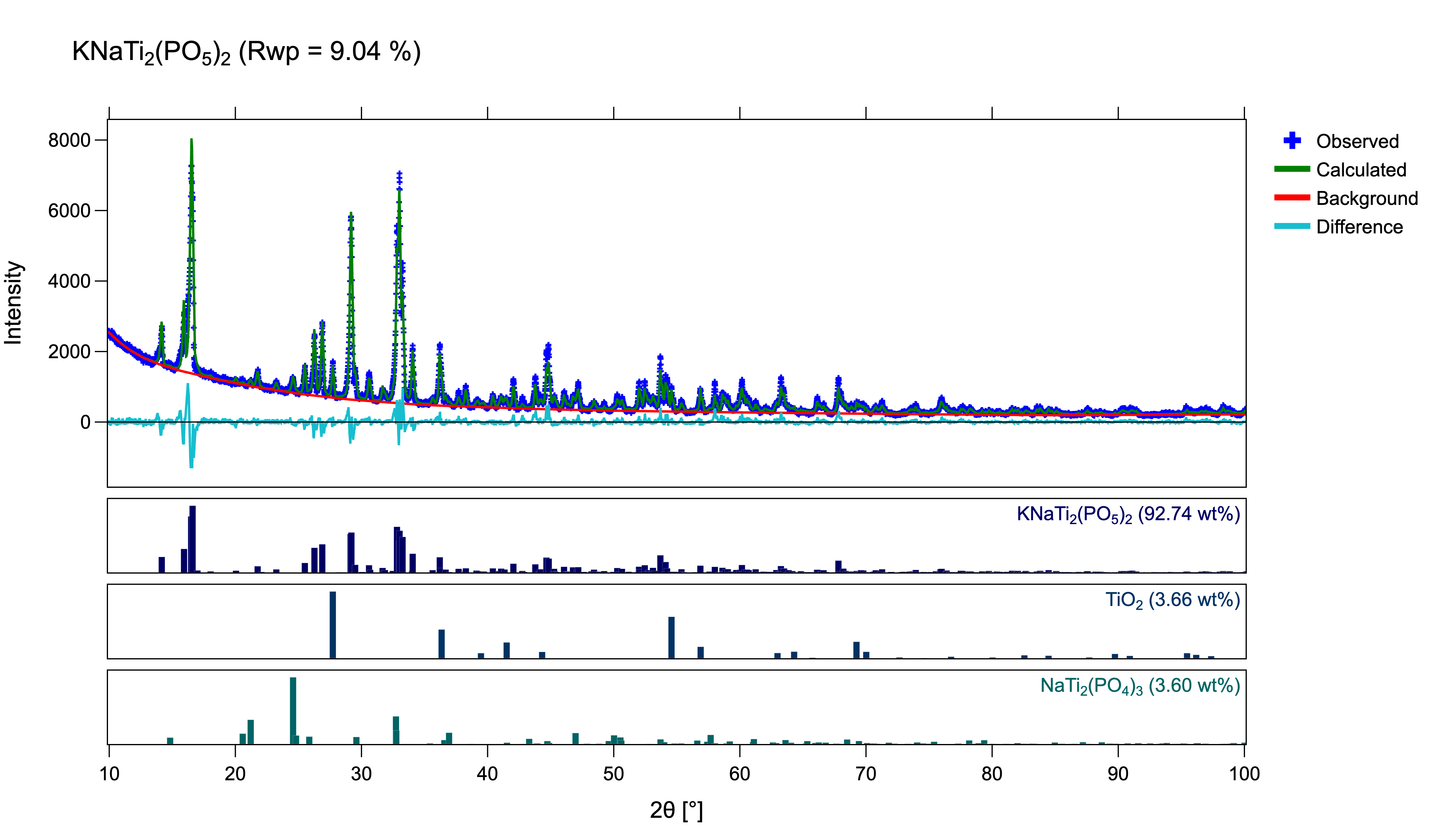

Supplement: Supplementary file 3 — This file contains the refined X-ray diffraction data from the successful syntheses performed by the A-Lab. The corresponding crystal structures used during refinement are also included in CIF format. [file 41586_2023_6734_MOESM3_ESM.zip › Manual_Refinement_Results/KNaTi2(PO5)2/KNaTi2(PO5)2.png]

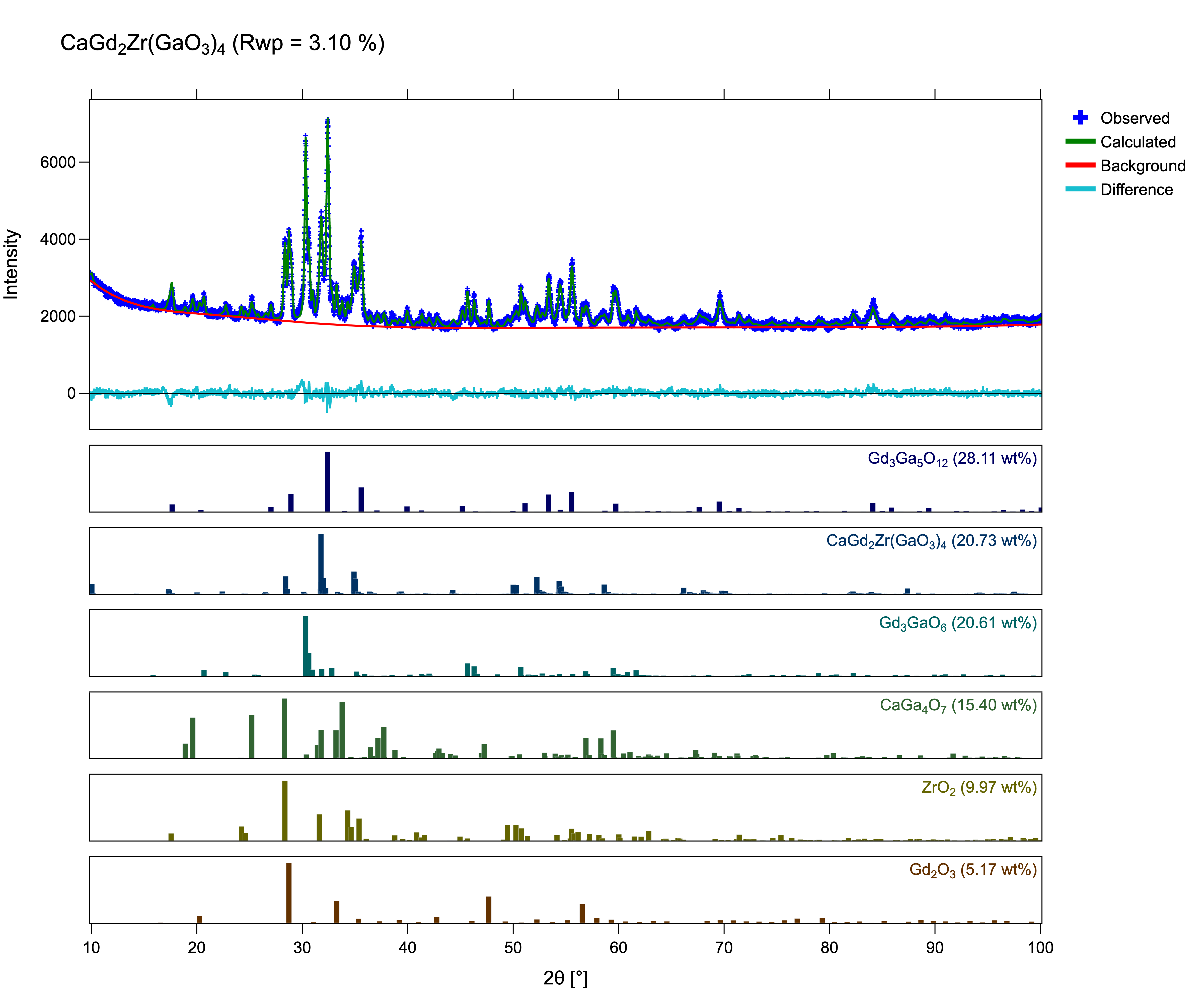

Supplement: Supplementary file 3 — This file contains the refined X-ray diffraction data from the successful syntheses performed by the A-Lab. The corresponding crystal structures used during refinement are also included in CIF format. [file 41586_2023_6734_MOESM3_ESM.zip › Manual_Refinement_Results/CaGd2Zr(GaO3)4/CaGd2Zr(GaO3)4.png]

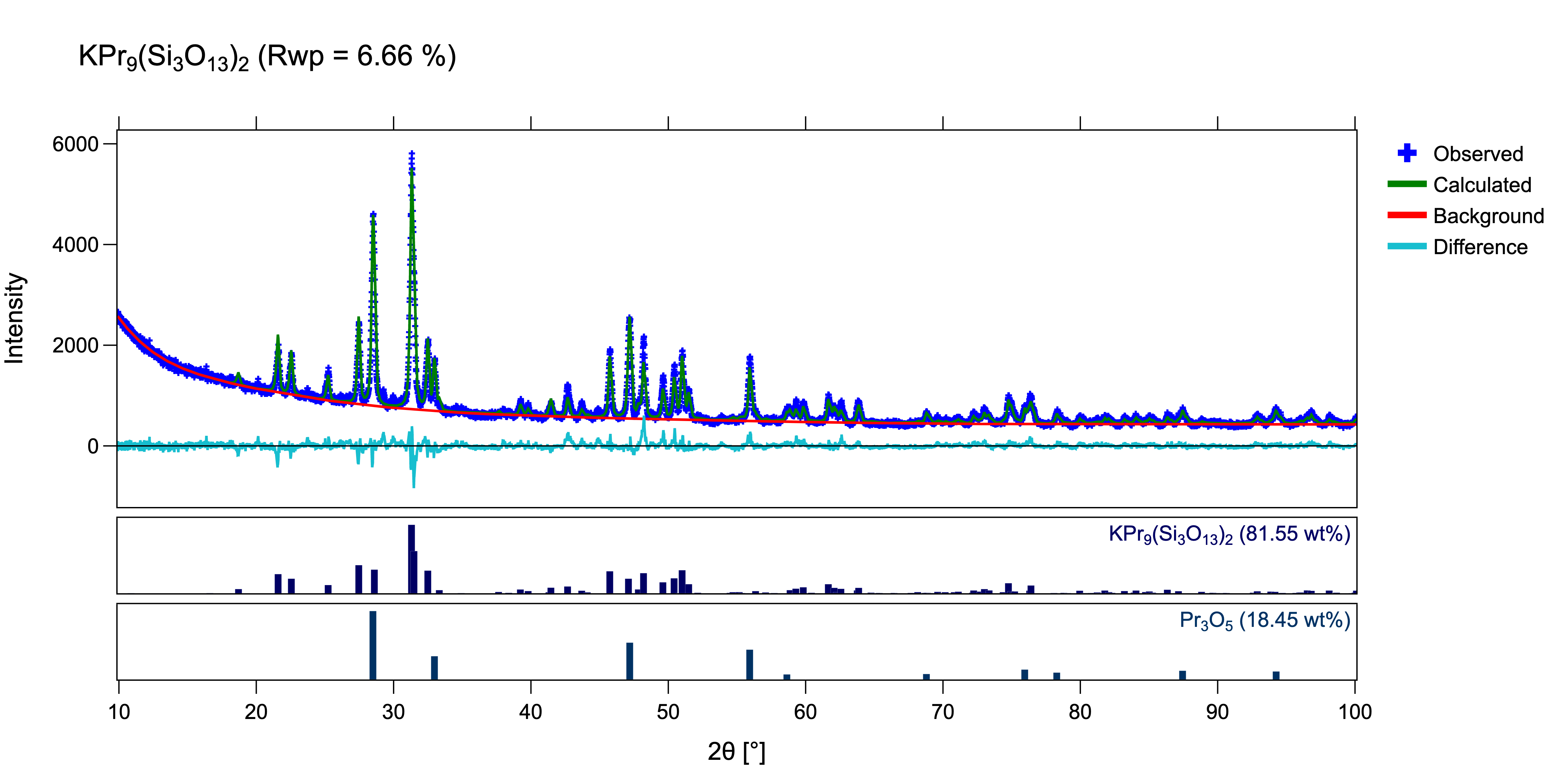

Supplement: Supplementary file 3 — This file contains the refined X-ray diffraction data from the successful syntheses performed by the A-Lab. The corresponding crystal structures used during refinement are also included in CIF format. [file 41586_2023_6734_MOESM3_ESM.zip › Manual_Refinement_Results/KPr9(Si3O13)2/KPr9(Si3O13)2.png]

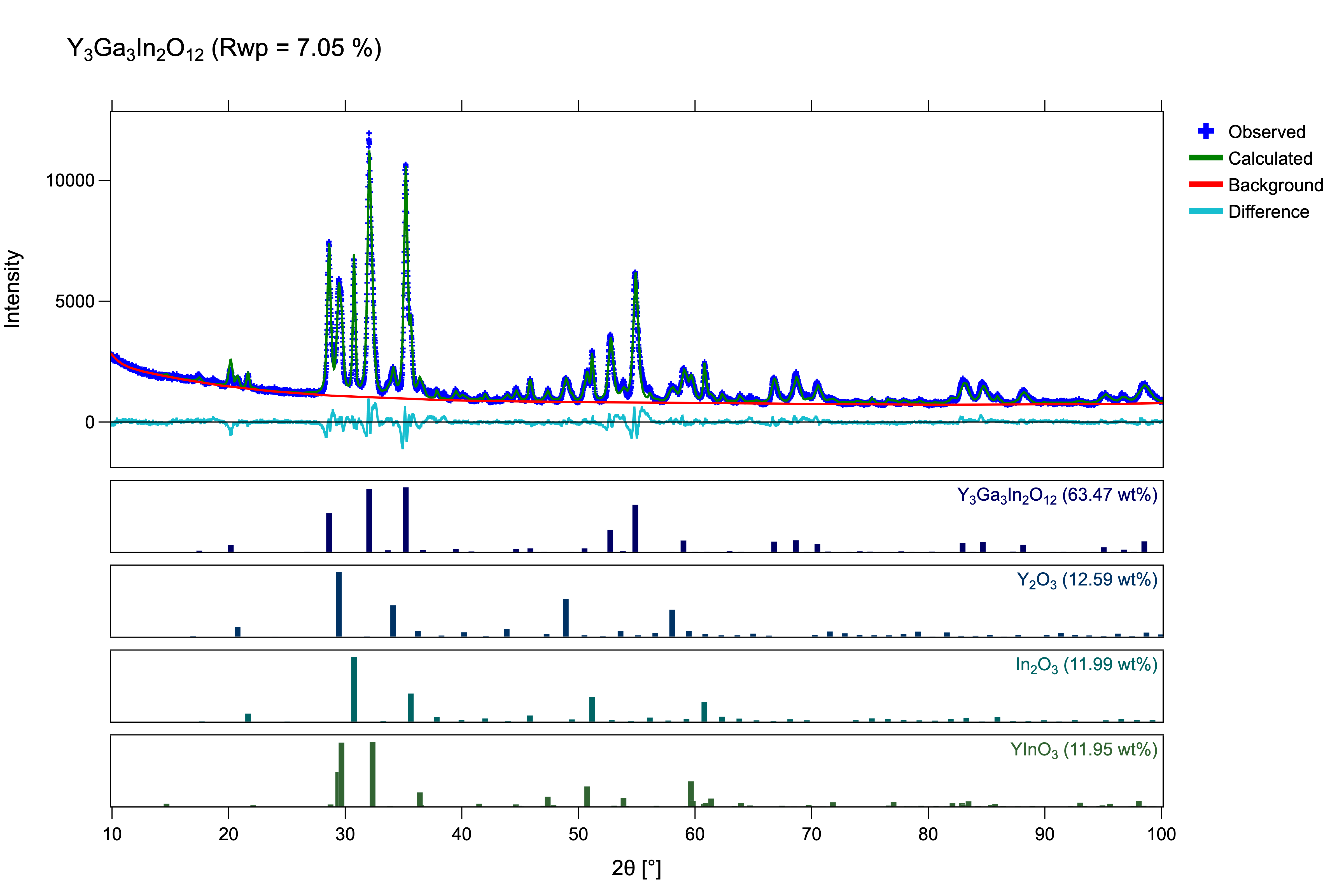

Supplement: Supplementary file 3 — This file contains the refined X-ray diffraction data from the successful syntheses performed by the A-Lab. The corresponding crystal structures used during refinement are also included in CIF format. [file 41586_2023_6734_MOESM3_ESM.zip › Manual_Refinement_Results/Y3Ga3In2O12/Y3Ga3In2O12.png]

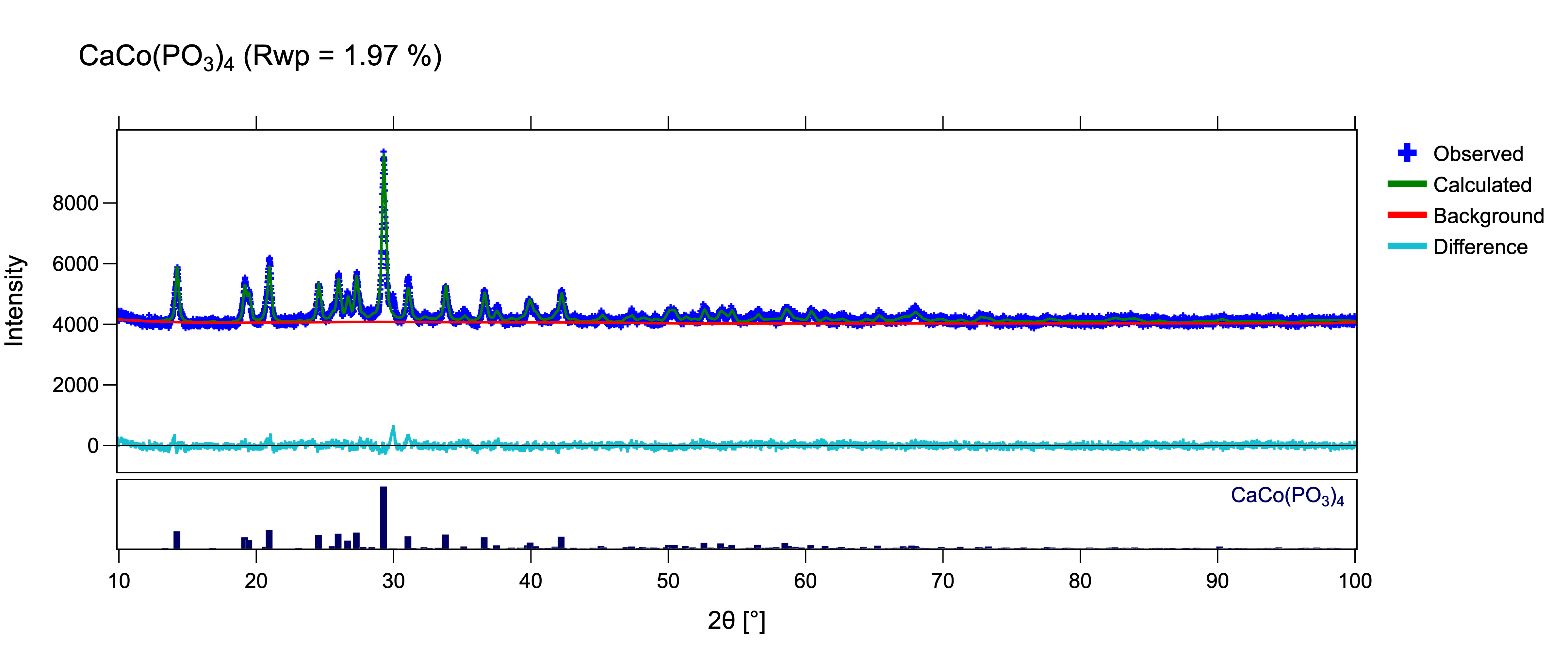

Supplement: Supplementary file 3 — This file contains the refined X-ray diffraction data from the successful syntheses performed by the A-Lab. The corresponding crystal structures used during refinement are also included in CIF format. [file 41586_2023_6734_MOESM3_ESM.zip › Manual_Refinement_Results/CaCo(PO3)4/CaCo(PO3)4.png]

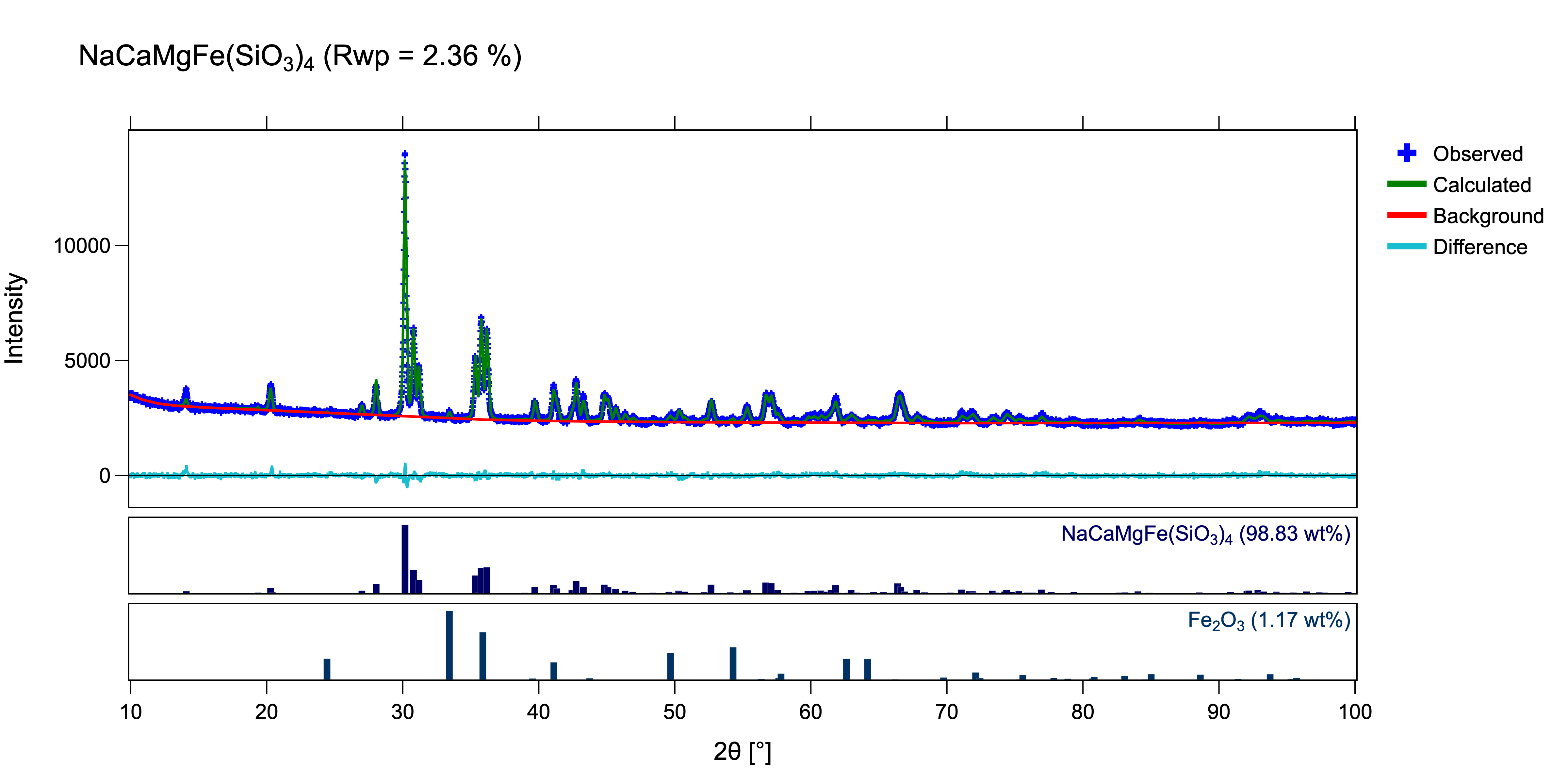

Supplement: Supplementary file 3 — This file contains the refined X-ray diffraction data from the successful syntheses performed by the A-Lab. The corresponding crystal structures used during refinement are also included in CIF format. [file 41586_2023_6734_MOESM3_ESM.zip › Manual_Refinement_Results/NaCaMgFe(SiO3)4/NaCaMgFe(SiO3)4.png]

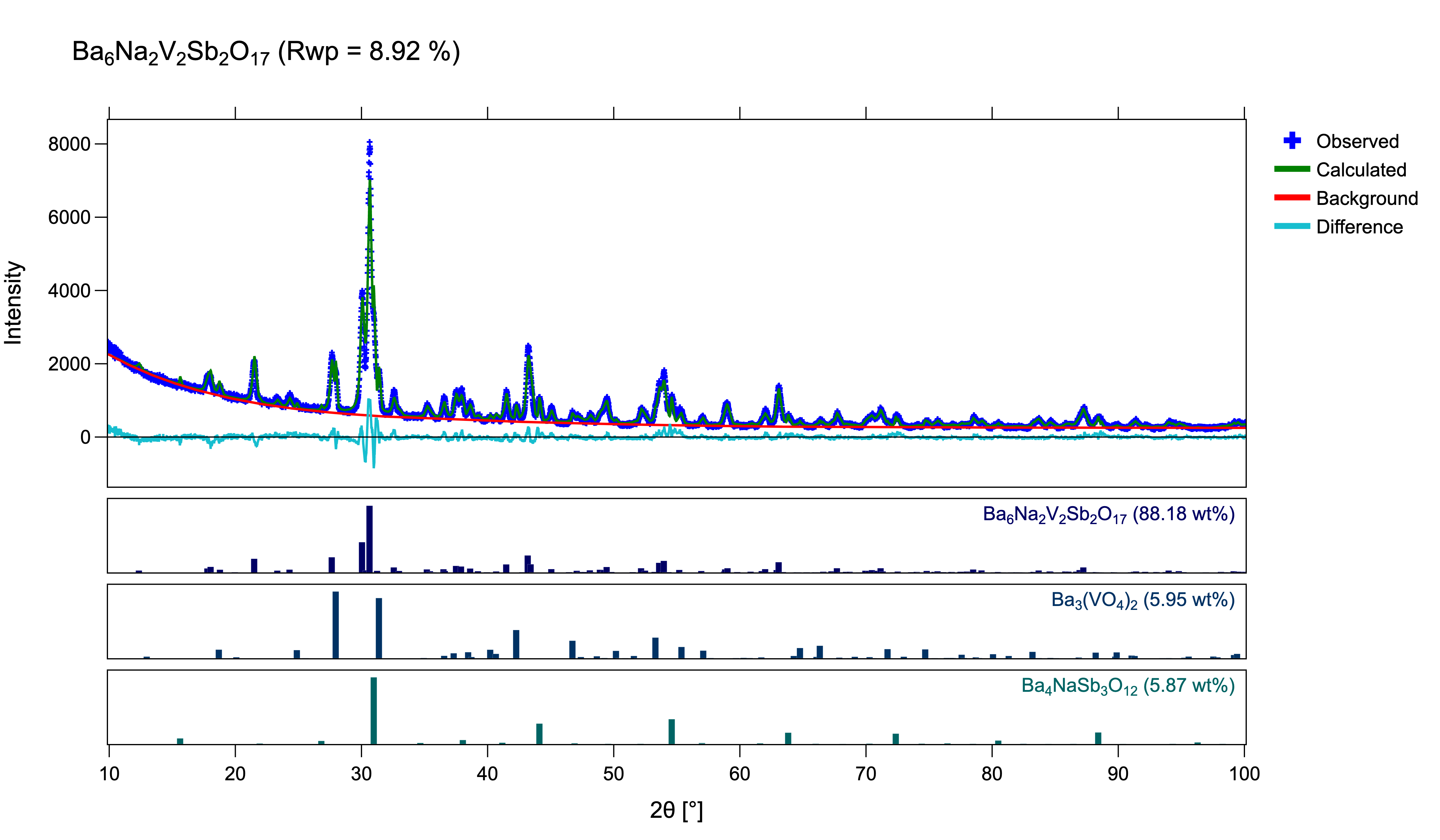

Supplement: Supplementary file 3 — This file contains the refined X-ray diffraction data from the successful syntheses performed by the A-Lab. The corresponding crystal structures used during refinement are also included in CIF format. [file 41586_2023_6734_MOESM3_ESM.zip › Manual_Refinement_Results/Ba6Na2V2Sb2O17/Ba6Na2V2Sb2O17.png]

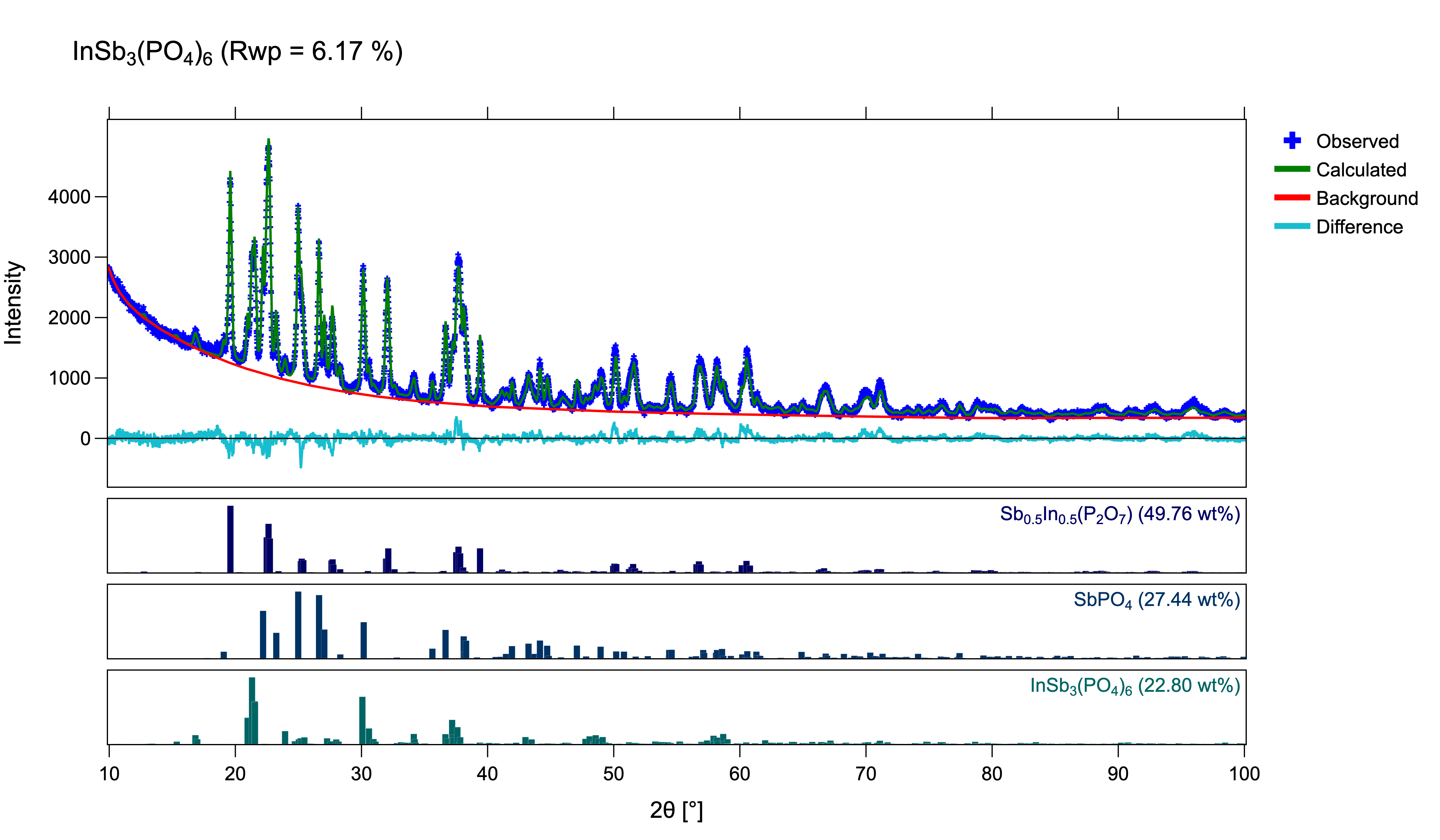

Supplement: Supplementary file 3 — This file contains the refined X-ray diffraction data from the successful syntheses performed by the A-Lab. The corresponding crystal structures used during refinement are also included in CIF format. [file 41586_2023_6734_MOESM3_ESM.zip › Manual_Refinement_Results/InSb3(PO4)6/InSb3(PO4)6.png]

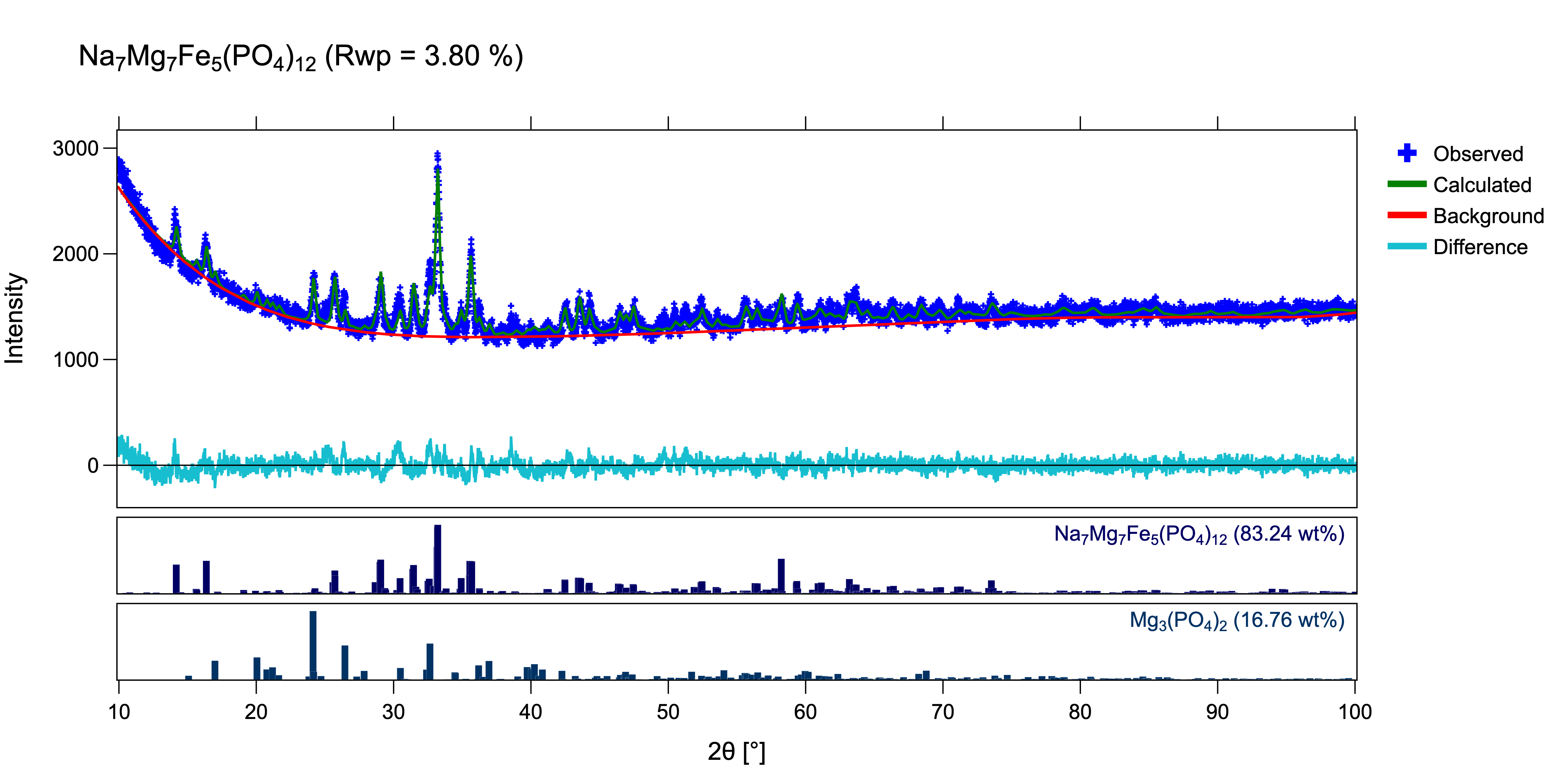

Supplement: Supplementary file 3 — This file contains the refined X-ray diffraction data from the successful syntheses performed by the A-Lab. The corresponding crystal structures used during refinement are also included in CIF format. [file 41586_2023_6734_MOESM3_ESM.zip › Manual_Refinement_Results/Na7Mg7Fe5(PO4)12/Na7Mg7Fe5(PO4)12.png]

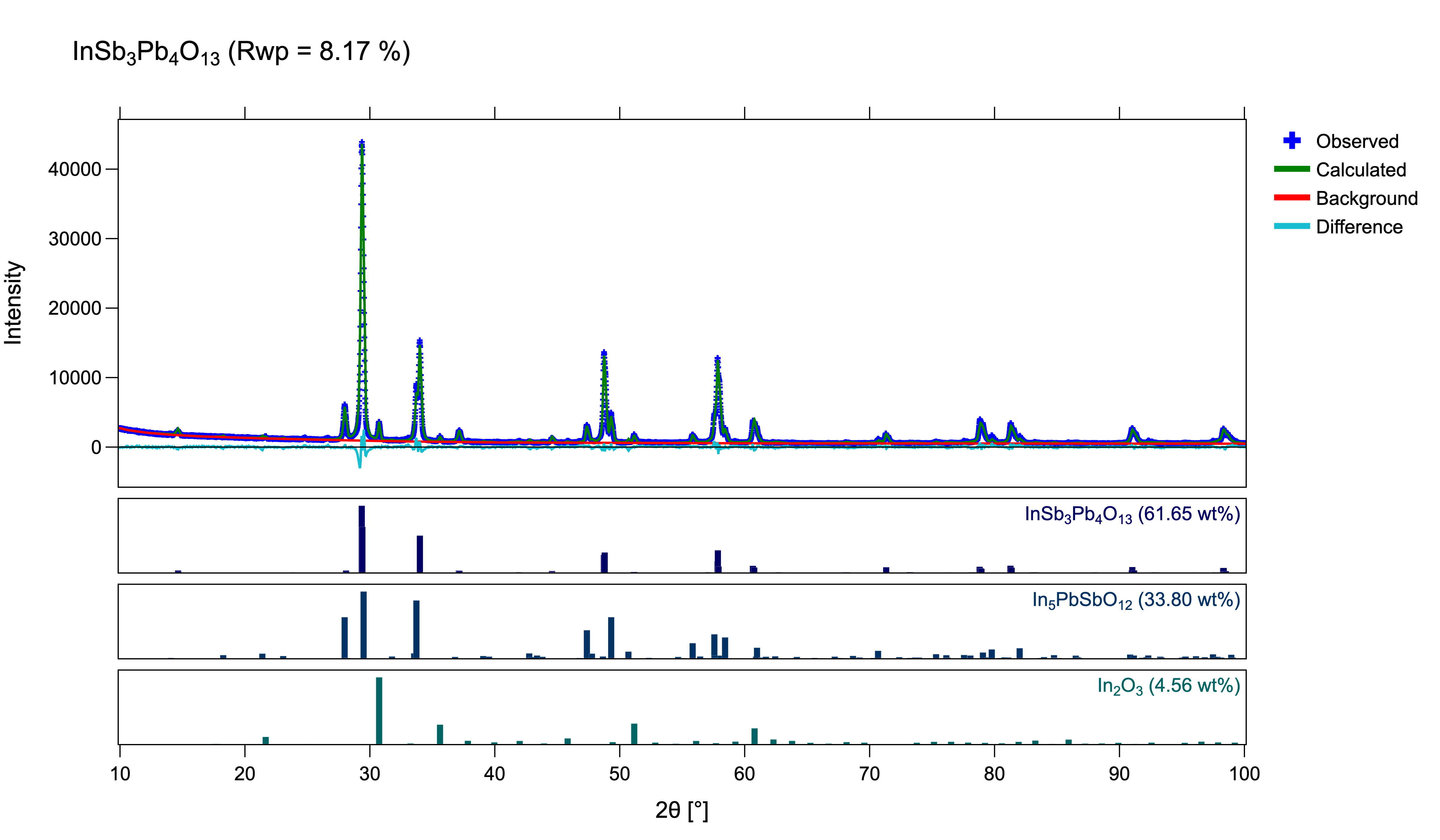

Supplement: Supplementary file 3 — This file contains the refined X-ray diffraction data from the successful syntheses performed by the A-Lab. The corresponding crystal structures used during refinement are also included in CIF format. [file 41586_2023_6734_MOESM3_ESM.zip › Manual_Refinement_Results/InSb3Pb4O13/InSb3Pb4O13.png]

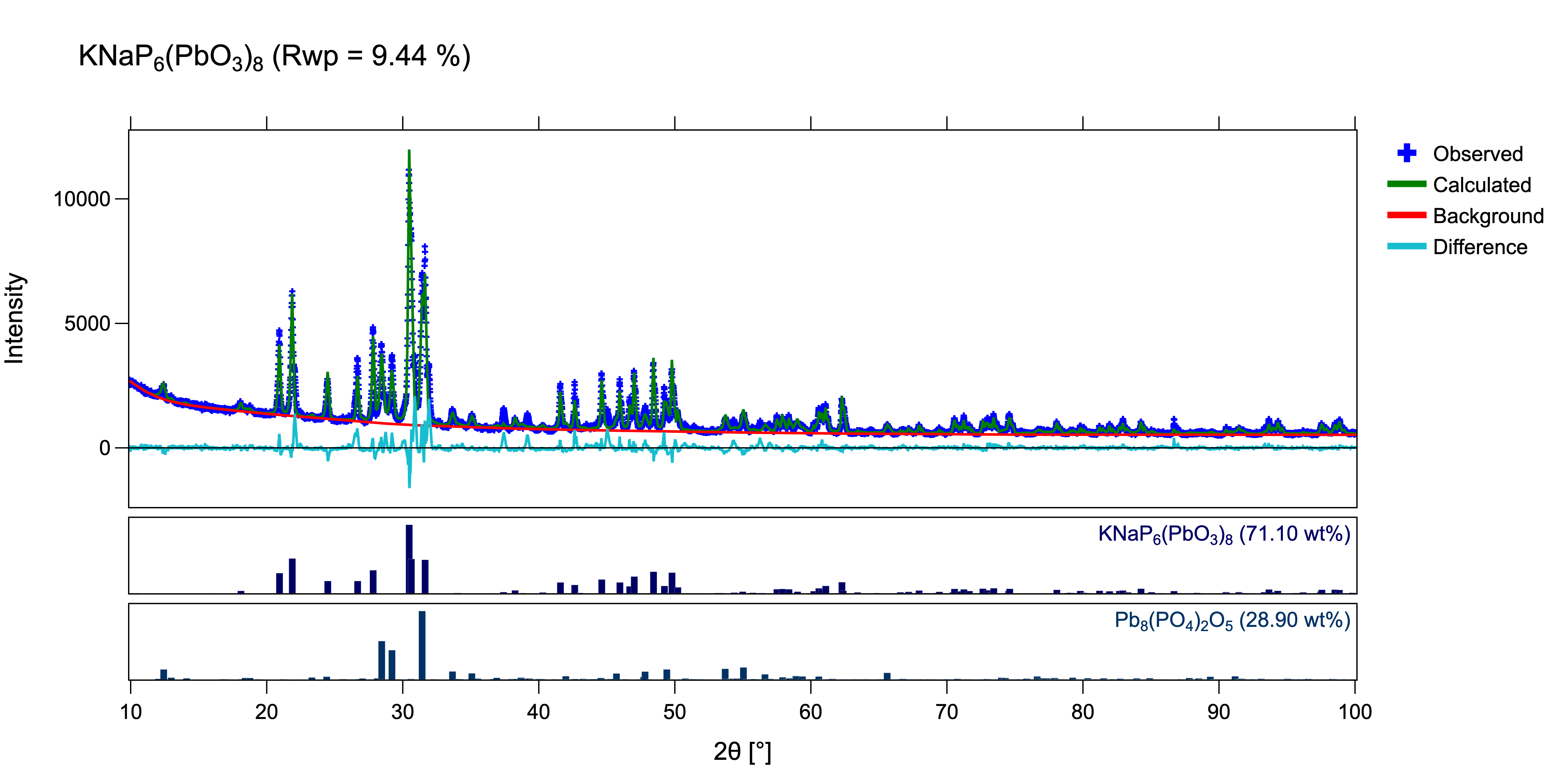

Supplement: Supplementary file 3 — This file contains the refined X-ray diffraction data from the successful syntheses performed by the A-Lab. The corresponding crystal structures used during refinement are also included in CIF format. [file 41586_2023_6734_MOESM3_ESM.zip › Manual_Refinement_Results/KNaP6(PbO3)8/KNaP6(PbO3)8.png]

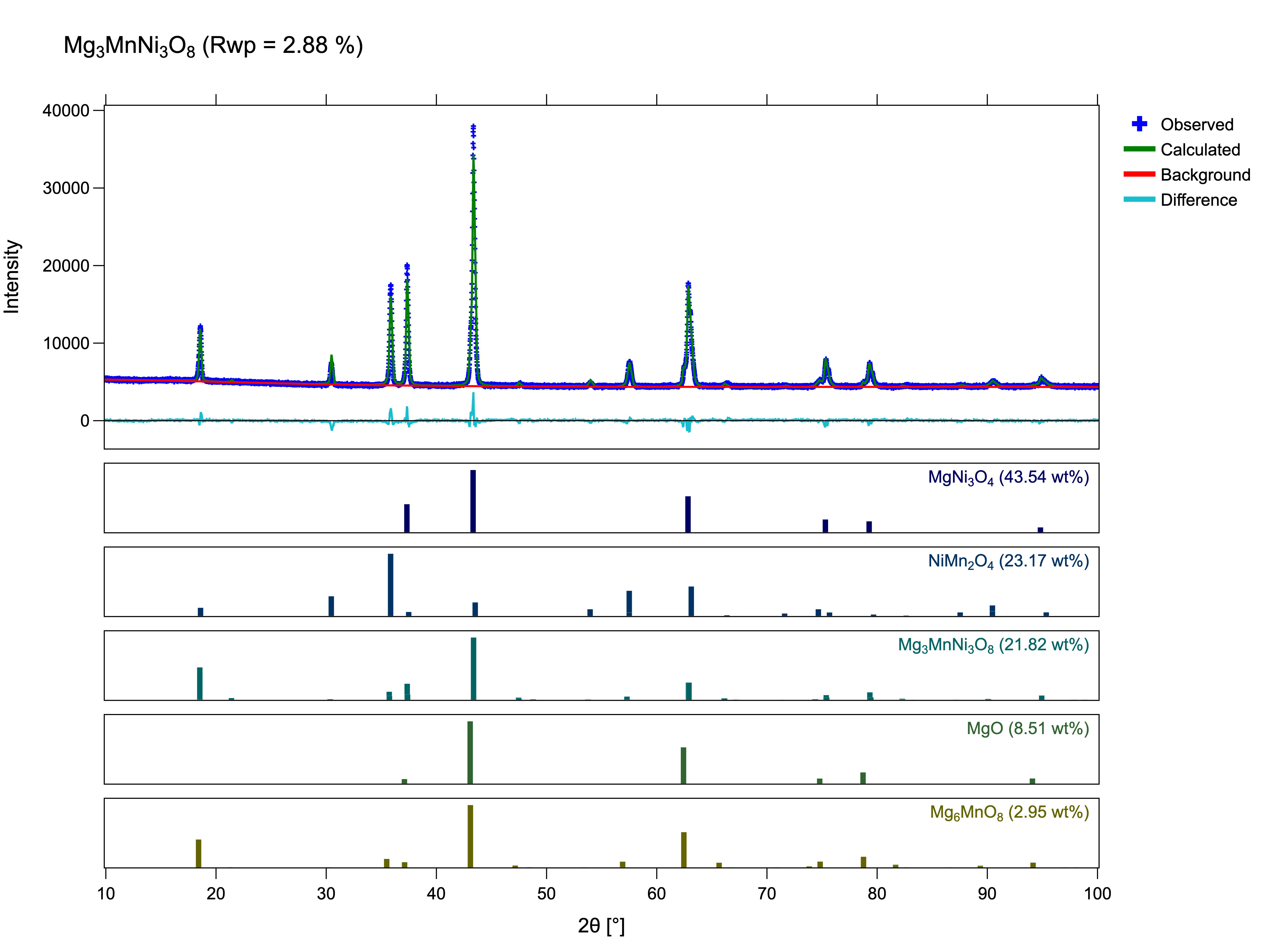

Supplement: Supplementary file 3 — This file contains the refined X-ray diffraction data from the successful syntheses performed by the A-Lab. The corresponding crystal structures used during refinement are also included in CIF format. [file 41586_2023_6734_MOESM3_ESM.zip › Manual_Refinement_Results/Mg3MnNi3O8/Mg3MnNi3O8.png]

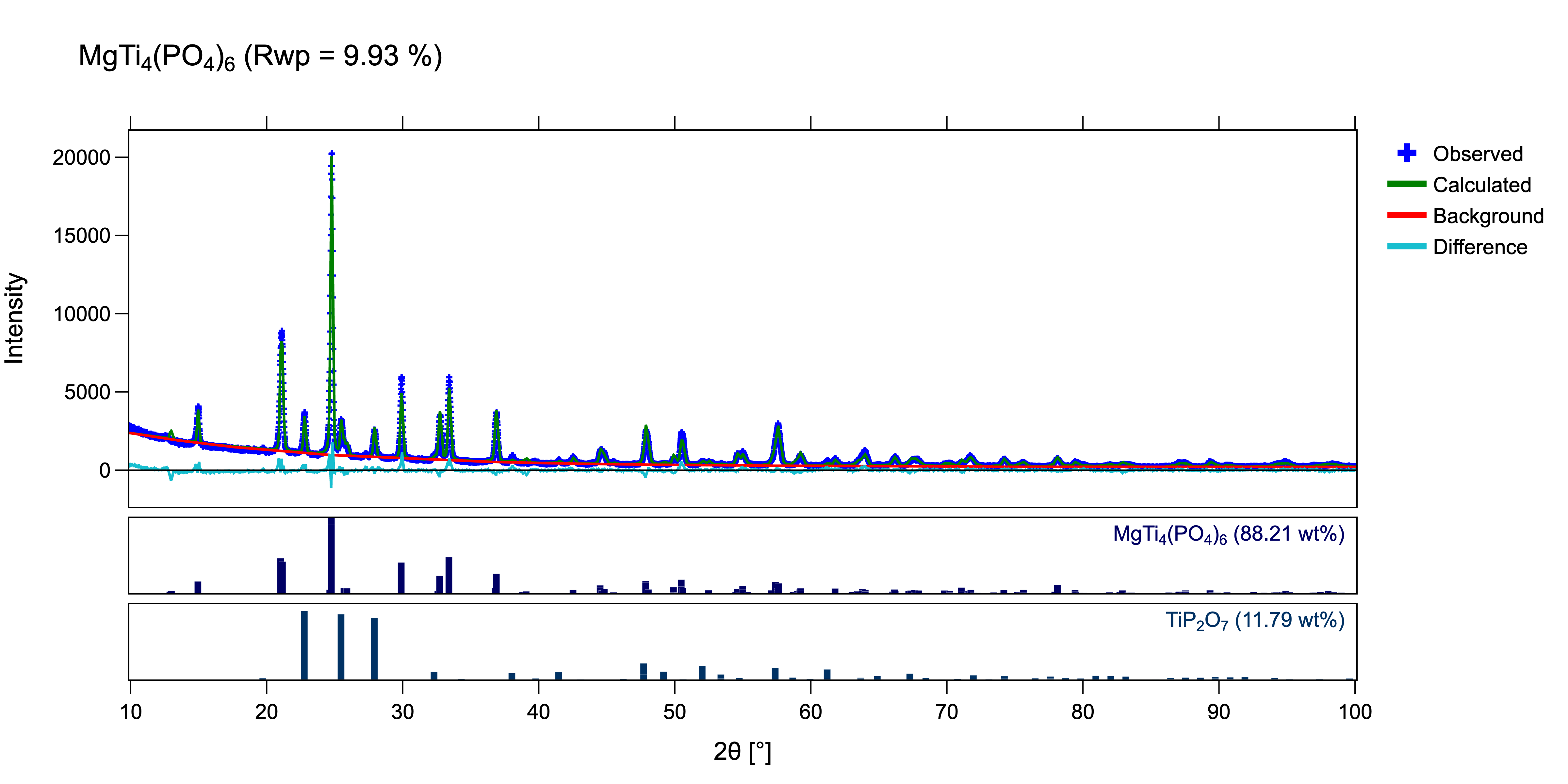

Supplement: Supplementary file 3 — This file contains the refined X-ray diffraction data from the successful syntheses performed by the A-Lab. The corresponding crystal structures used during refinement are also included in CIF format. [file 41586_2023_6734_MOESM3_ESM.zip › Manual_Refinement_Results/MgTi4(PO4)6/MgTi4(PO4)6.png]

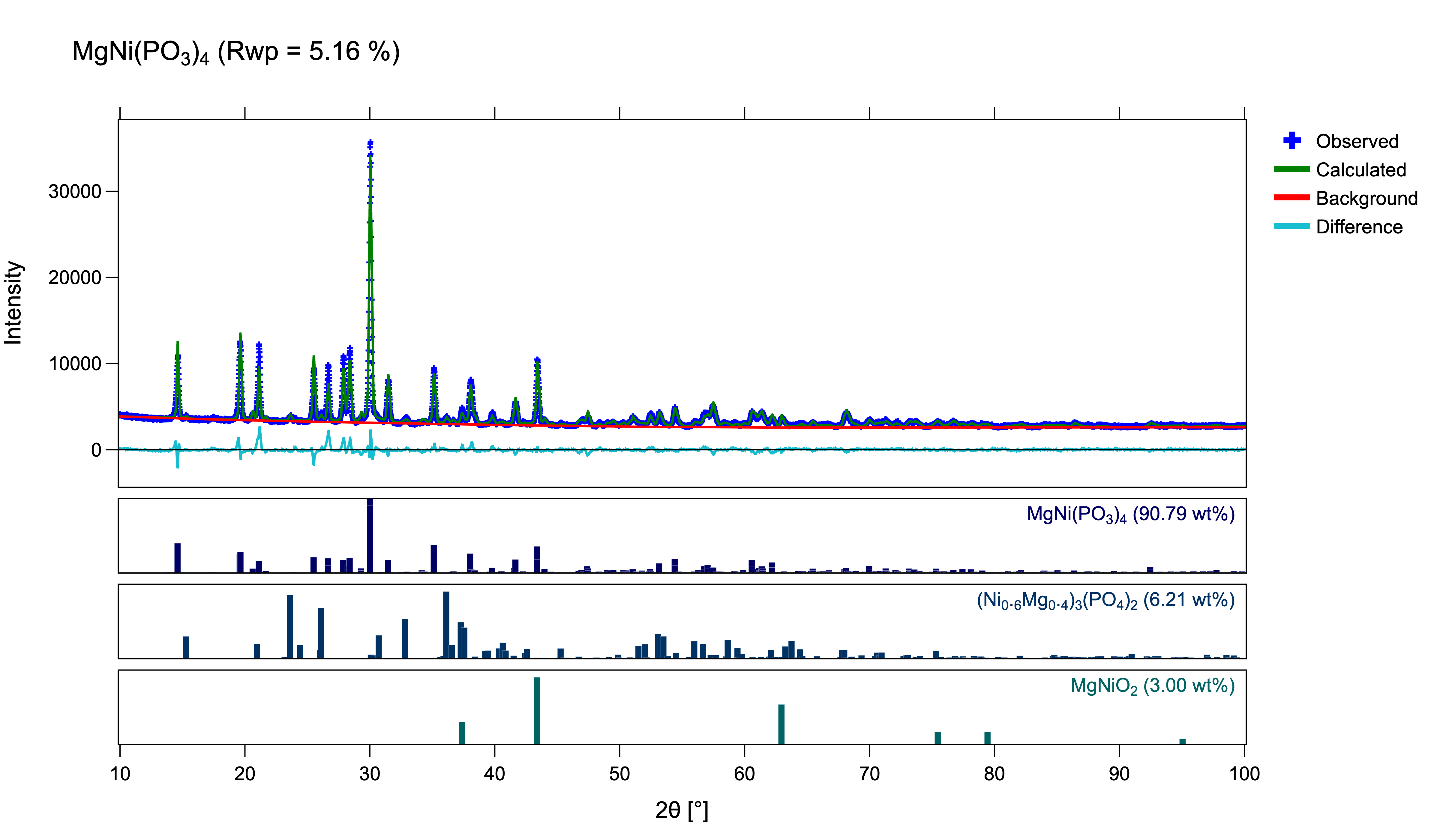

Supplement: Supplementary file 3 — This file contains the refined X-ray diffraction data from the successful syntheses performed by the A-Lab. The corresponding crystal structures used during refinement are also included in CIF format. [file 41586_2023_6734_MOESM3_ESM.zip › Manual_Refinement_Results/MgNi(PO3)4/MgNi(PO3)4.png]

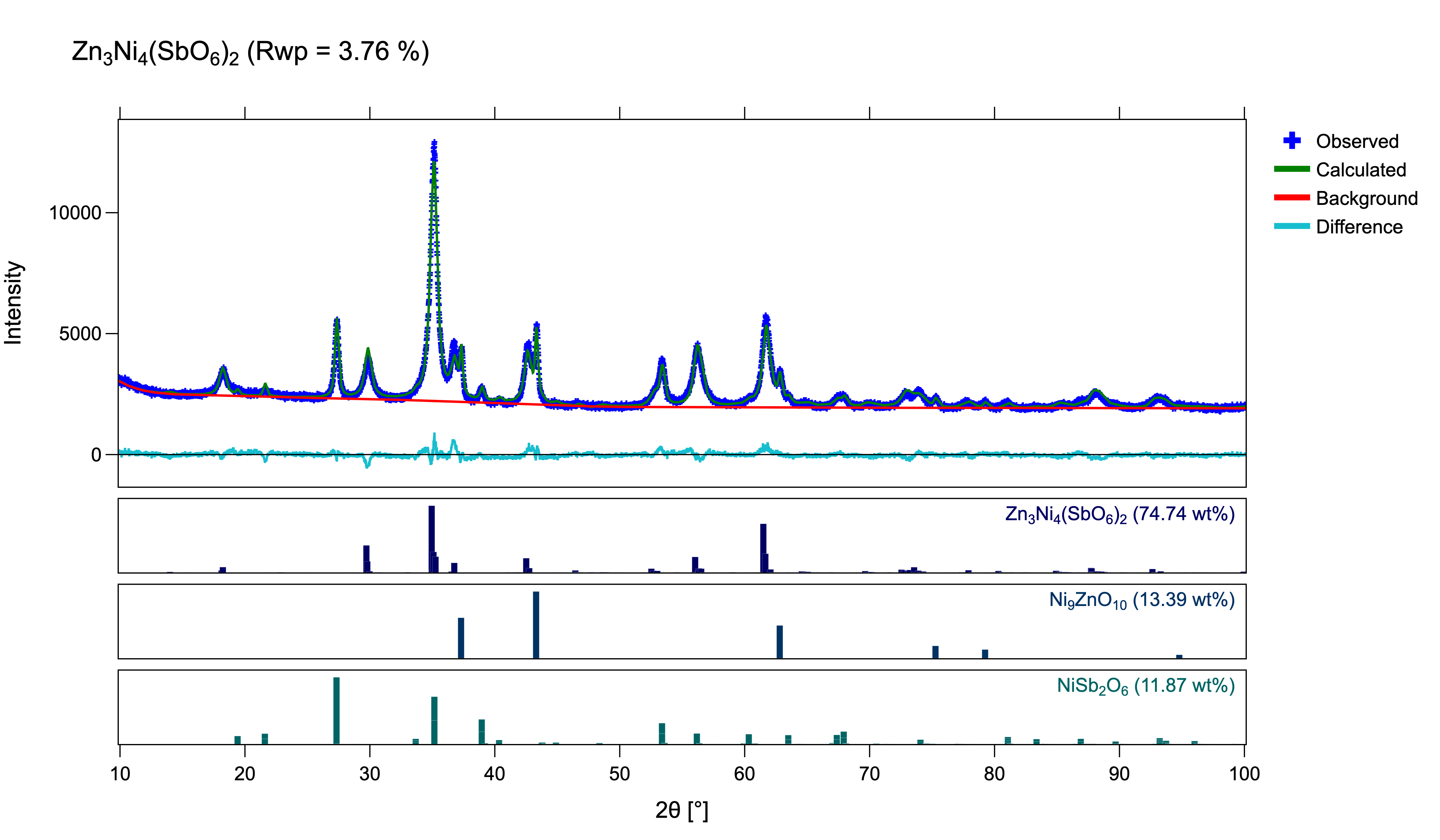

Supplement: Supplementary file 3 — This file contains the refined X-ray diffraction data from the successful syntheses performed by the A-Lab. The corresponding crystal structures used during refinement are also included in CIF format. [file 41586_2023_6734_MOESM3_ESM.zip › Manual_Refinement_Results/Zn3Ni4(SbO6)2/Zn3Ni4(SbO6)2.png]

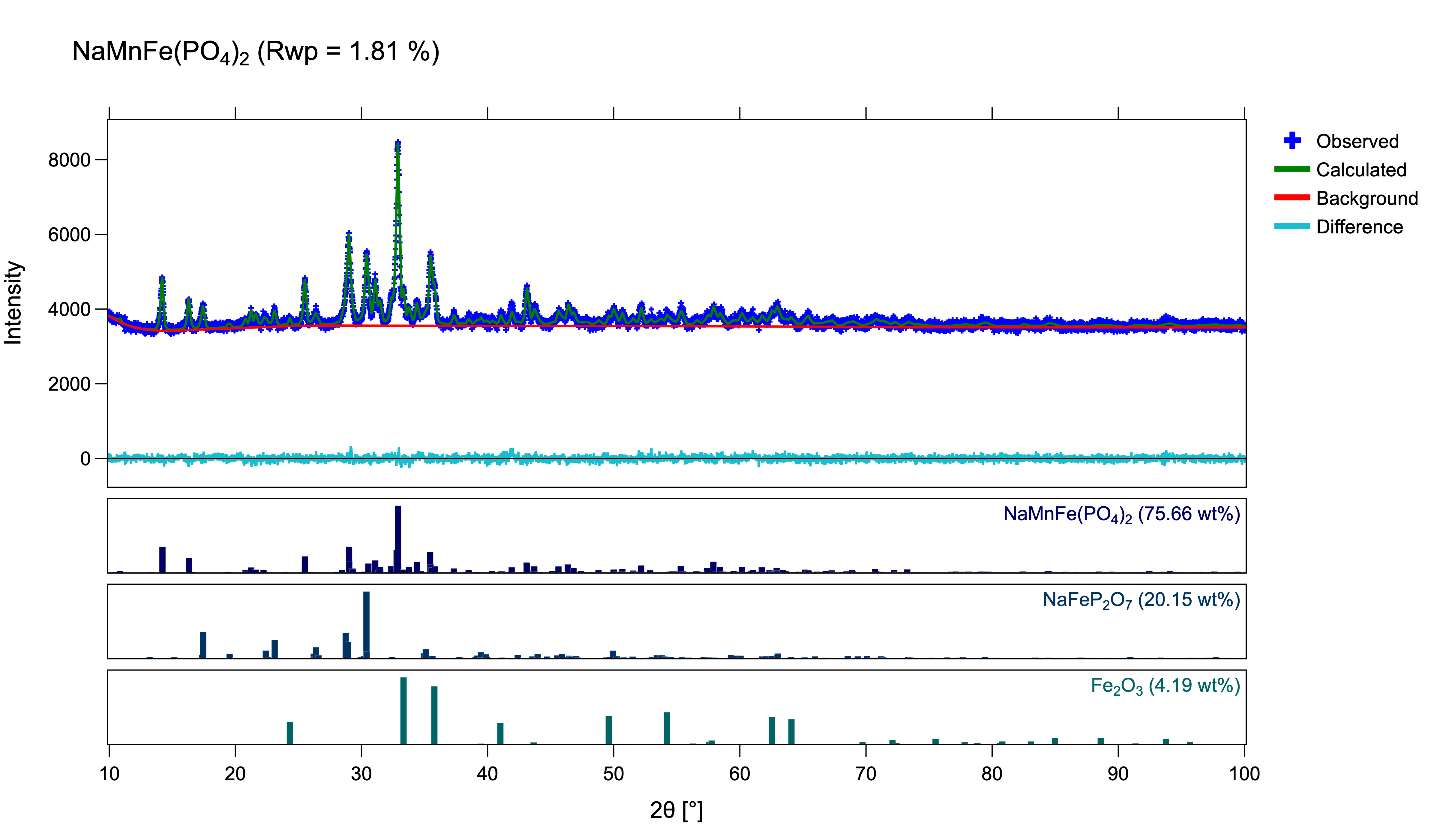

Supplement: Supplementary file 3 — This file contains the refined X-ray diffraction data from the successful syntheses performed by the A-Lab. The corresponding crystal structures used during refinement are also included in CIF format. [file 41586_2023_6734_MOESM3_ESM.zip › Manual_Refinement_Results/NaMnFe(PO4)2/NaMnFe(PO4)2.png]

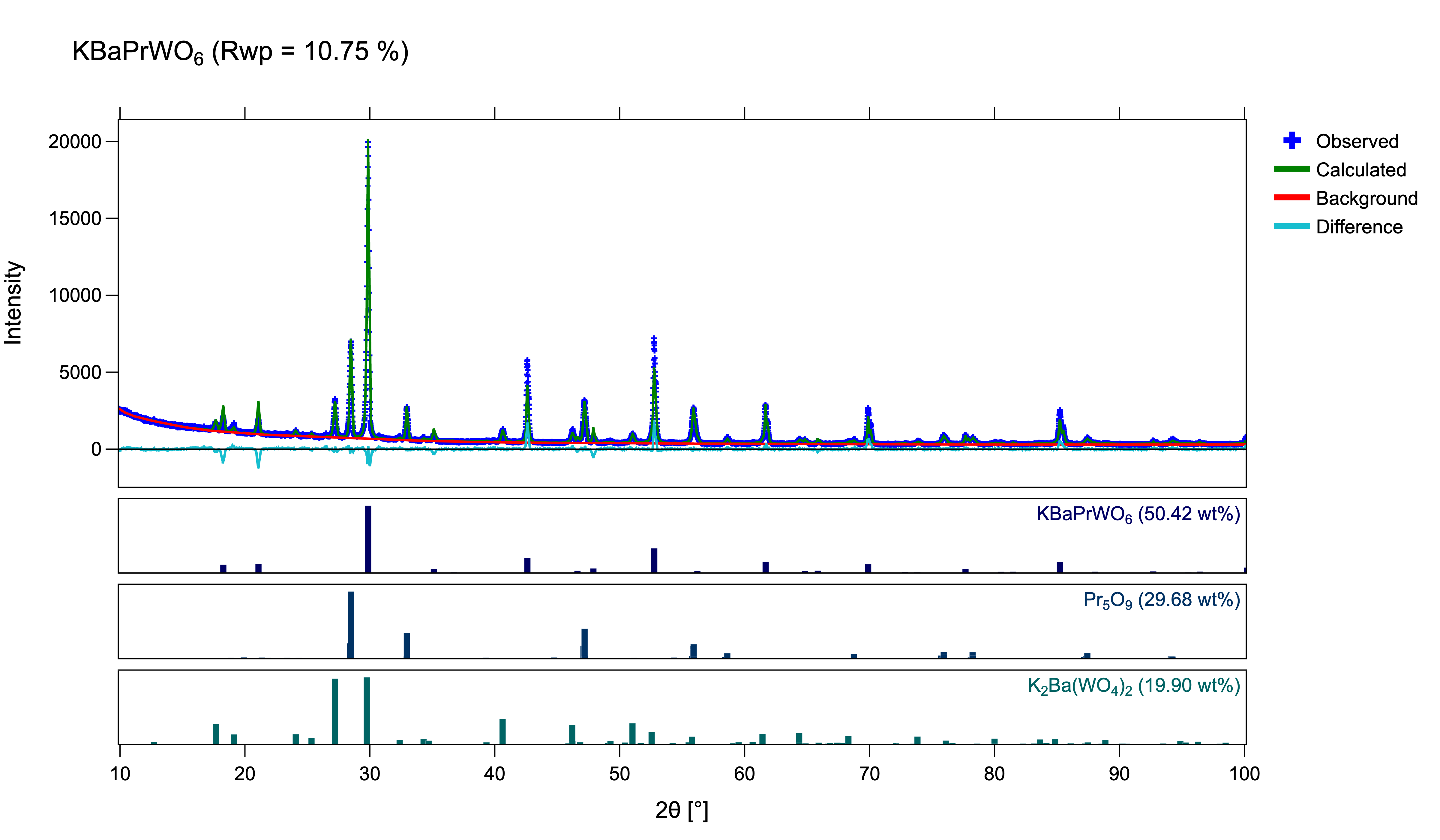

Supplement: Supplementary file 3 — This file contains the refined X-ray diffraction data from the successful syntheses performed by the A-Lab. The corresponding crystal structures used during refinement are also included in CIF format. [file 41586_2023_6734_MOESM3_ESM.zip › Manual_Refinement_Results/KBaPrWO6/KBaPrWO6.png]

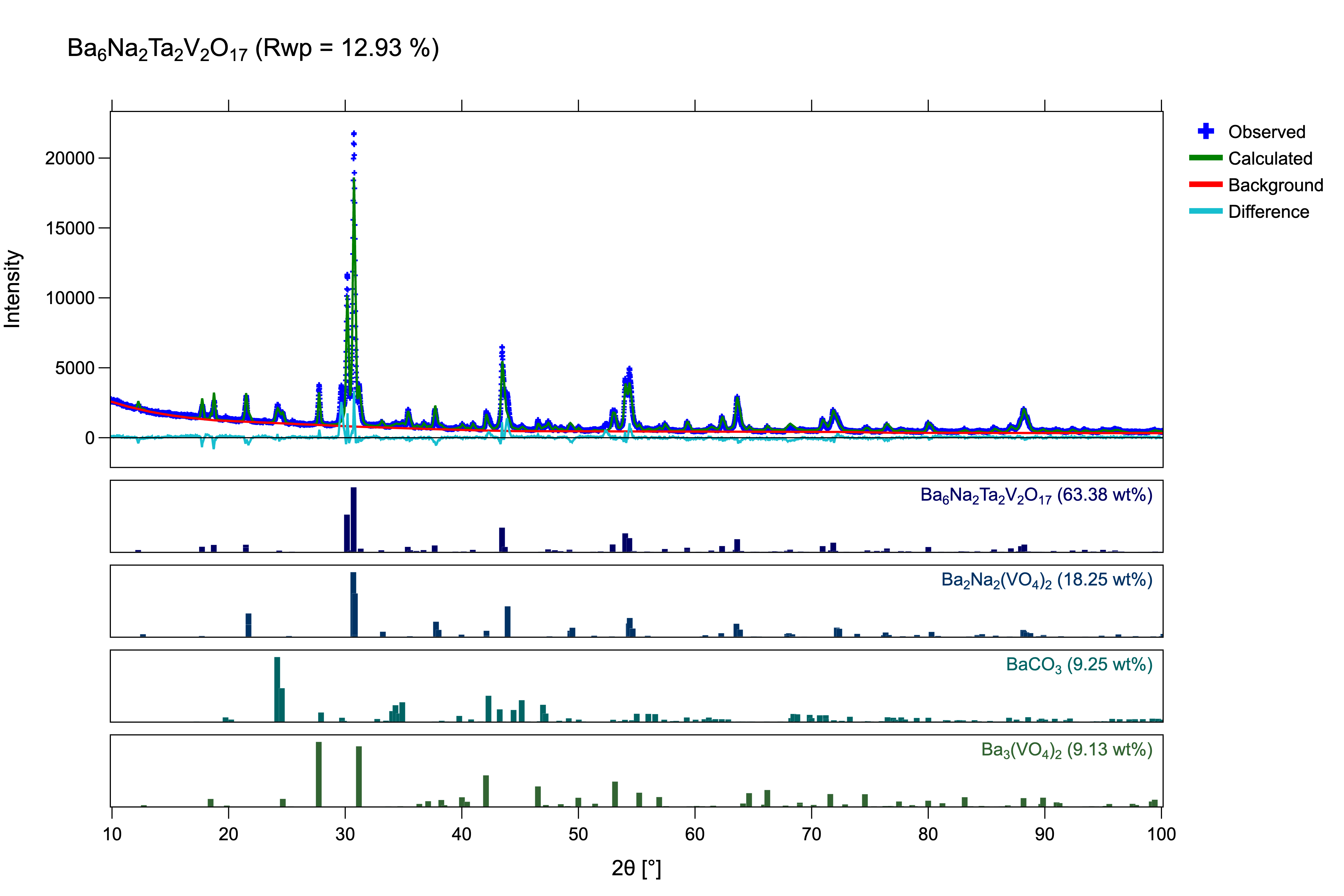

Supplement: Supplementary file 3 — This file contains the refined X-ray diffraction data from the successful syntheses performed by the A-Lab. The corresponding crystal structures used during refinement are also included in CIF format. [file 41586_2023_6734_MOESM3_ESM.zip › Manual_Refinement_Results/Ba6Na2Ta2V2O17/Ba6Na2Ta2V2O17.png]

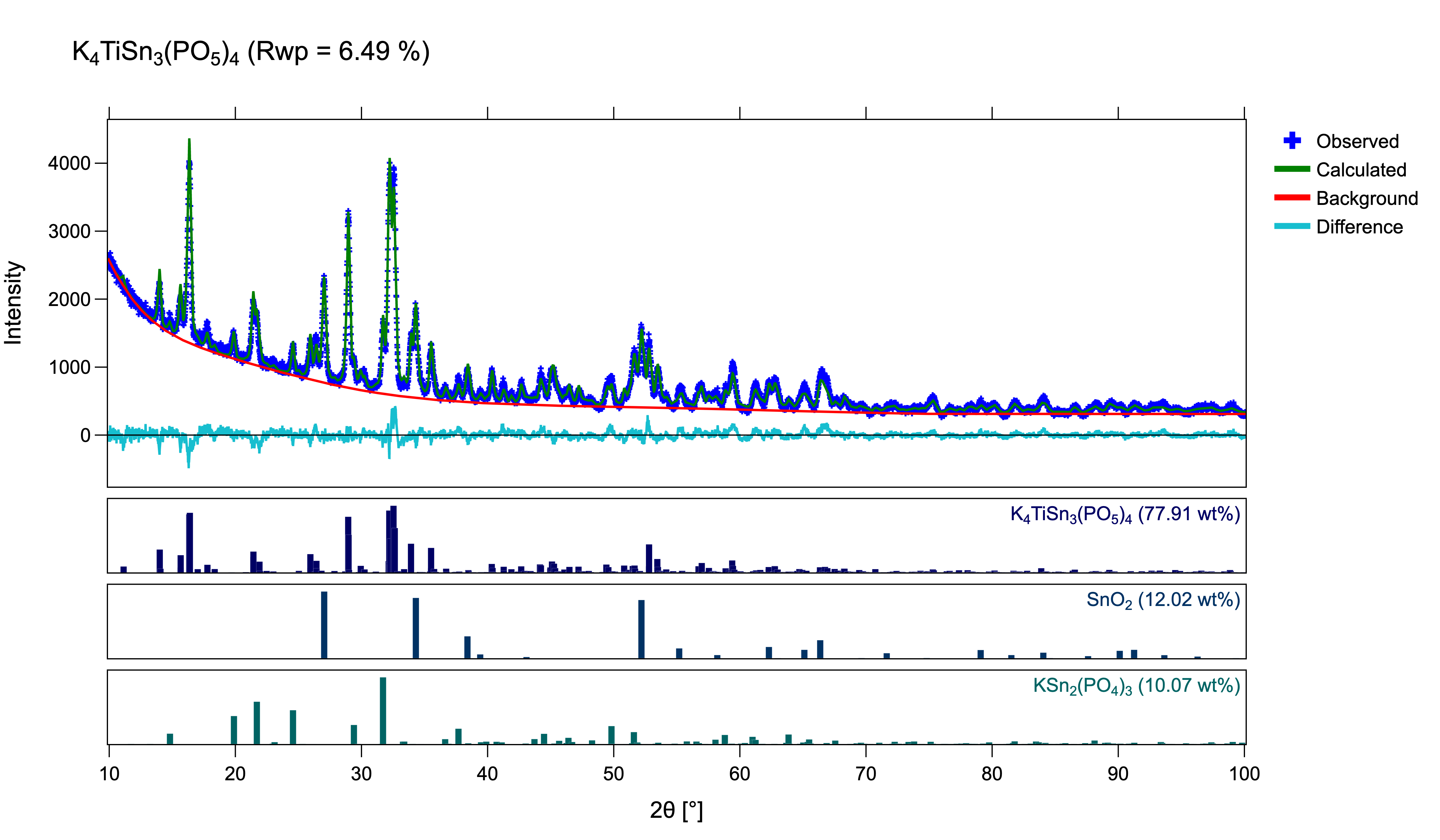

Supplement: Supplementary file 3 — This file contains the refined X-ray diffraction data from the successful syntheses performed by the A-Lab. The corresponding crystal structures used during refinement are also included in CIF format. [file 41586_2023_6734_MOESM3_ESM.zip › Manual_Refinement_Results/K4TiSn3(PO5)4/K4TiSn3(PO5)4.png]

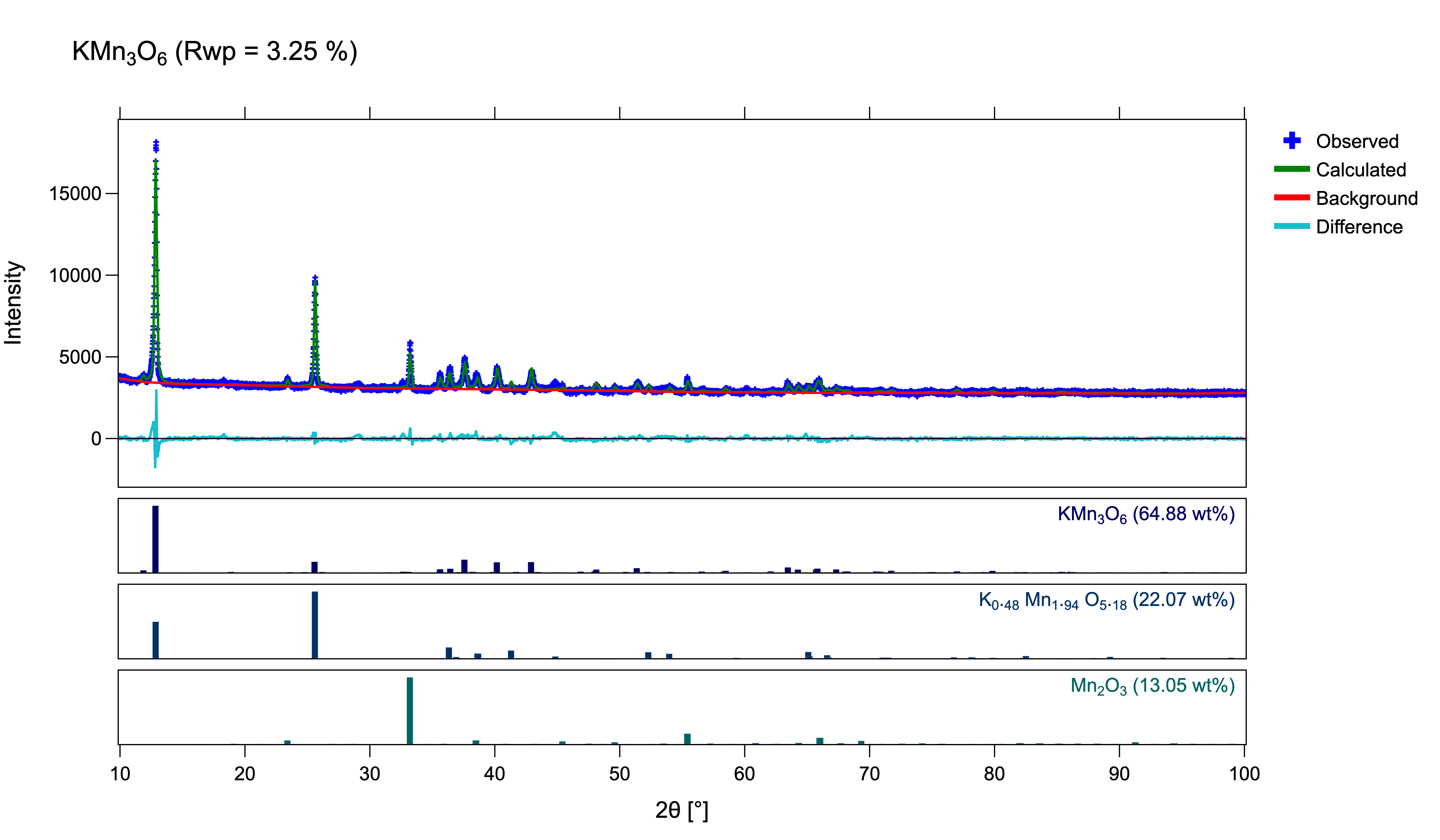

Supplement: Supplementary file 3 — This file contains the refined X-ray diffraction data from the successful syntheses performed by the A-Lab. The corresponding crystal structures used during refinement are also included in CIF format. [file 41586_2023_6734_MOESM3_ESM.zip › Manual_Refinement_Results/KMn3O6/KMn3O6.png]

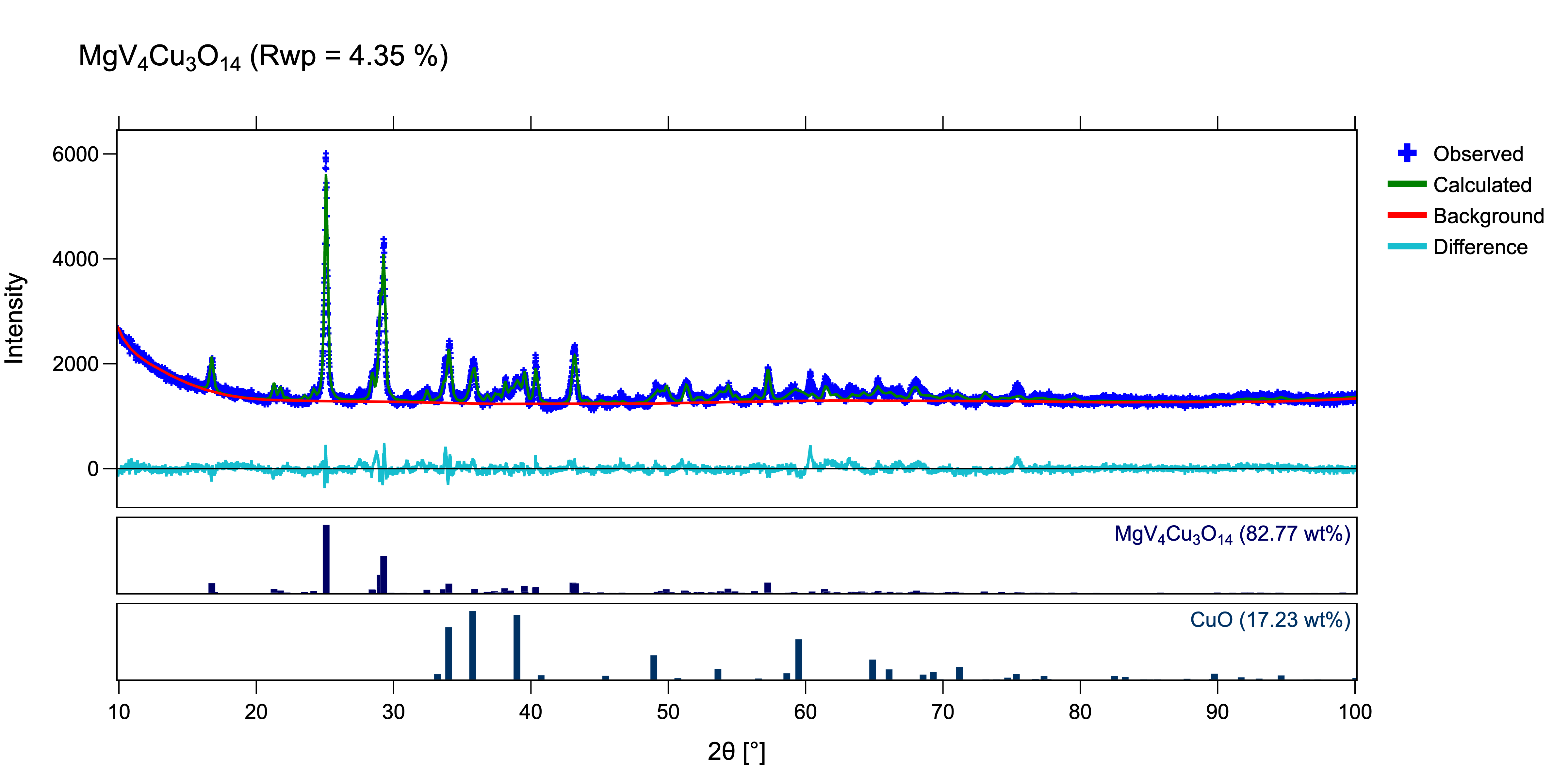

Supplement: Supplementary file 3 — This file contains the refined X-ray diffraction data from the successful syntheses performed by the A-Lab. The corresponding crystal structures used during refinement are also included in CIF format. [file 41586_2023_6734_MOESM3_ESM.zip › Manual_Refinement_Results/MgV4Cu3O14/MgV4Cu3O14.png]

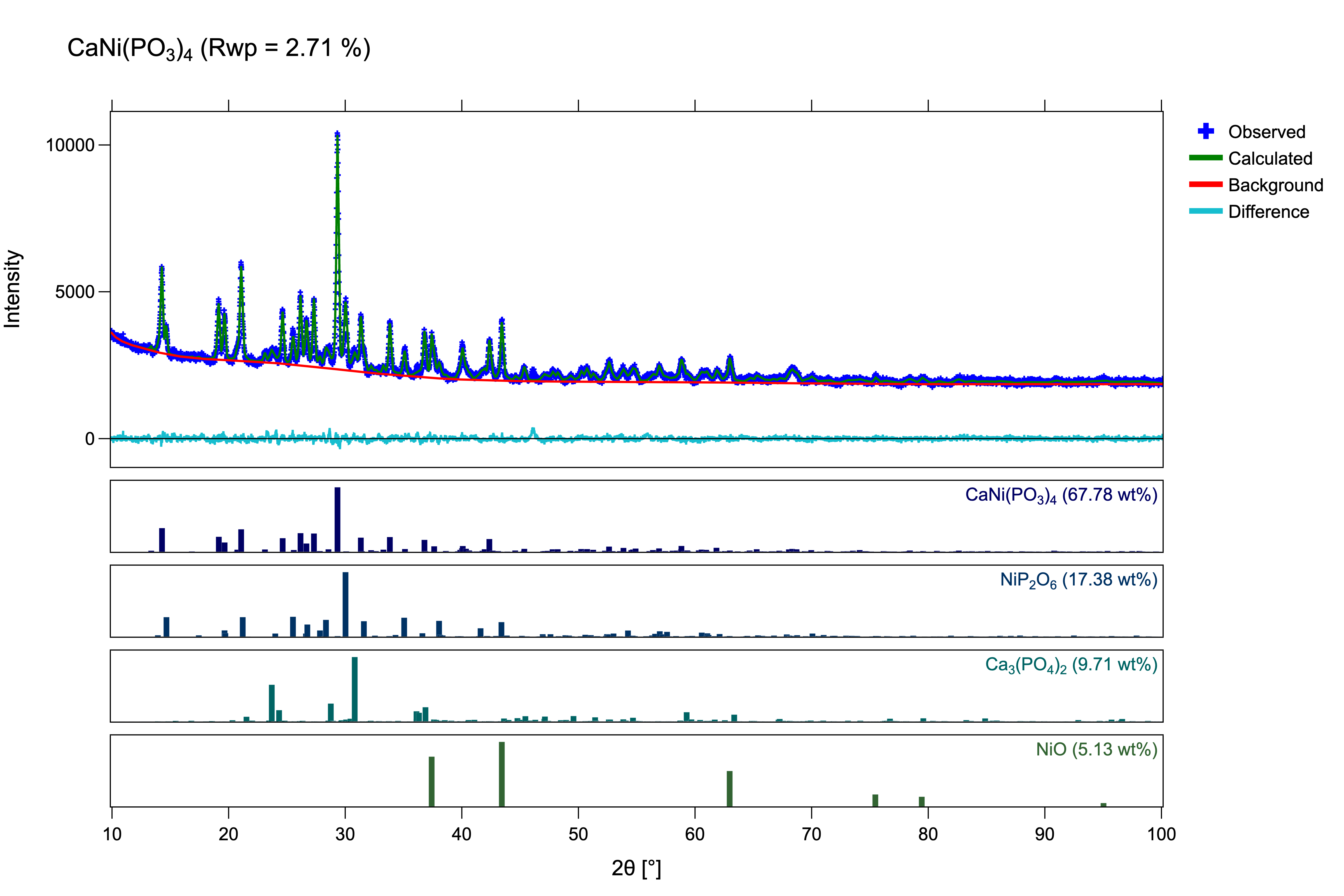

Supplement: Supplementary file 3 — This file contains the refined X-ray diffraction data from the successful syntheses performed by the A-Lab. The corresponding crystal structures used during refinement are also included in CIF format. [file 41586_2023_6734_MOESM3_ESM.zip › Manual_Refinement_Results/CaNi(PO3)4/CaNi(PO3)4.png]

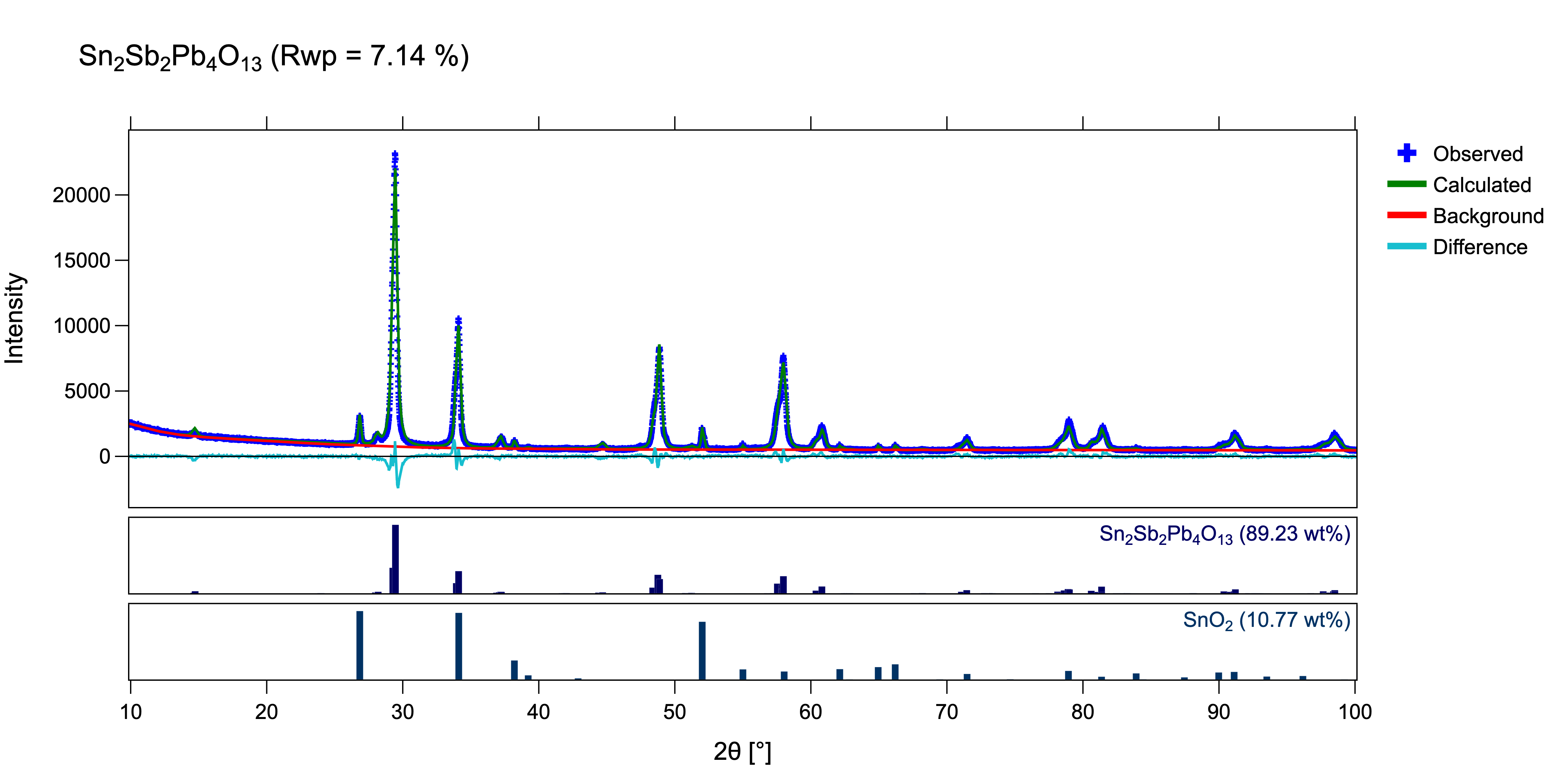

Supplement: Supplementary file 3 — This file contains the refined X-ray diffraction data from the successful syntheses performed by the A-Lab. The corresponding crystal structures used during refinement are also included in CIF format. [file 41586_2023_6734_MOESM3_ESM.zip › Manual_Refinement_Results/Sn2Sb2Pb4O13/Sn2Sb2Pb4O13.png]

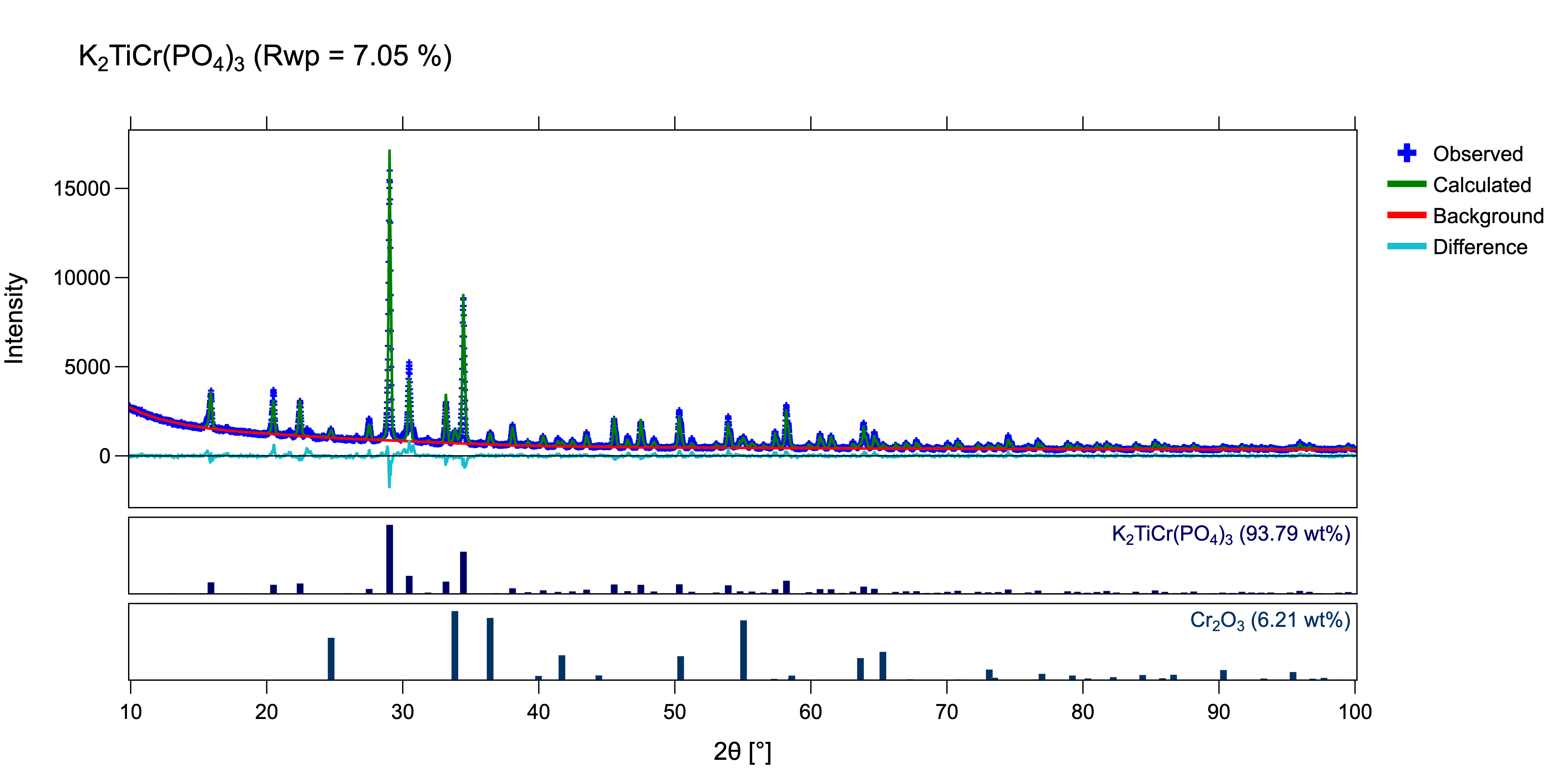

Supplement: Supplementary file 3 — This file contains the refined X-ray diffraction data from the successful syntheses performed by the A-Lab. The corresponding crystal structures used during refinement are also included in CIF format. [file 41586_2023_6734_MOESM3_ESM.zip › Manual_Refinement_Results/K2TiCr(PO4)3/K2TiCr(PO4)3.png]

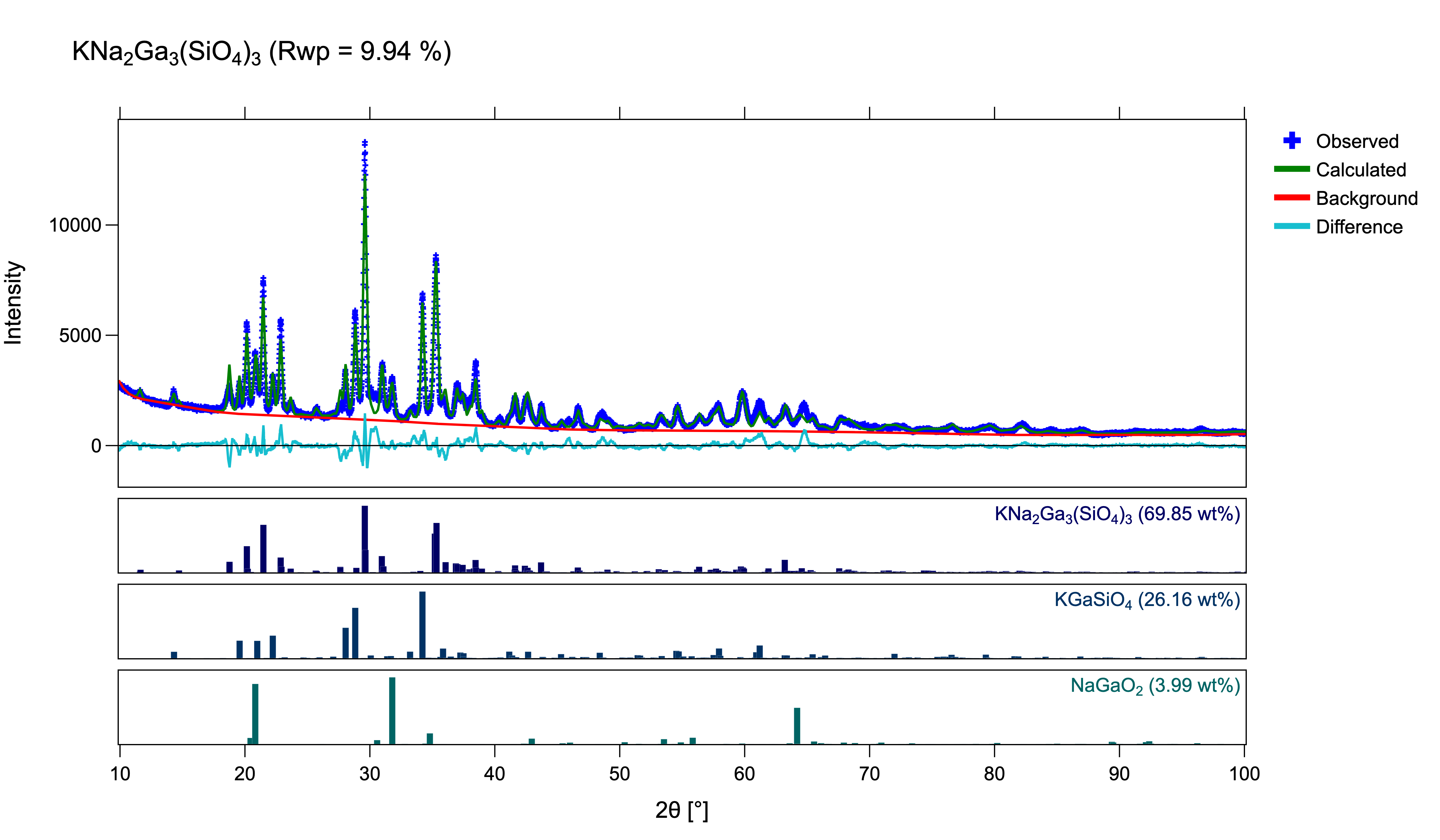

Supplement: Supplementary file 3 — This file contains the refined X-ray diffraction data from the successful syntheses performed by the A-Lab. The corresponding crystal structures used during refinement are also included in CIF format. [file 41586_2023_6734_MOESM3_ESM.zip › Manual_Refinement_Results/KNa2Ga3(SiO4)3/KNa2Ga3(SiO4)3.png]

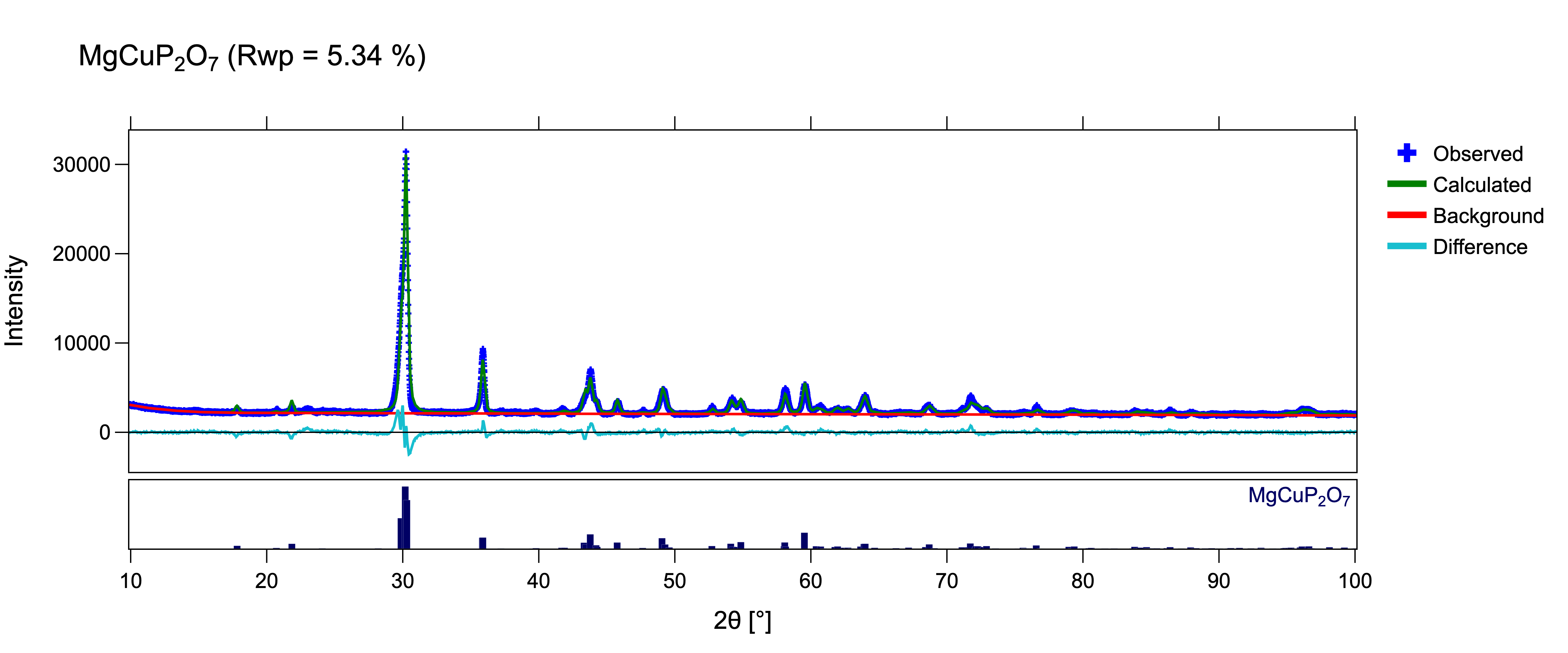

Supplement: Supplementary file 3 — This file contains the refined X-ray diffraction data from the successful syntheses performed by the A-Lab. The corresponding crystal structures used during refinement are also included in CIF format. [file 41586_2023_6734_MOESM3_ESM.zip › Manual_Refinement_Results/MgCuP2O7/MgCuP2O7.png]

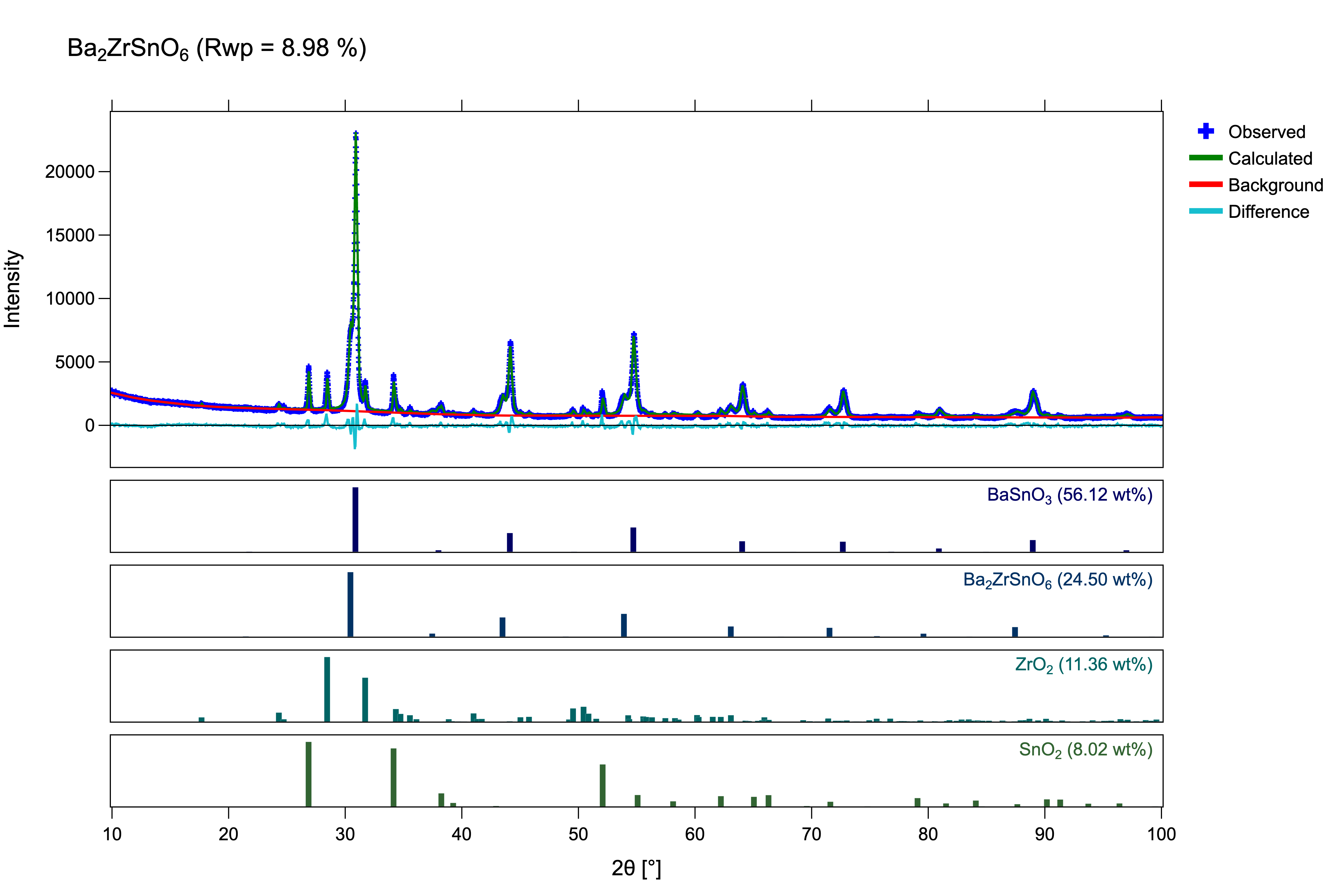

Supplement: Supplementary file 3 — This file contains the refined X-ray diffraction data from the successful syntheses performed by the A-Lab. The corresponding crystal structures used during refinement are also included in CIF format. [file 41586_2023_6734_MOESM3_ESM.zip › Manual_Refinement_Results/Ba2ZrSnO6/Ba2ZrSnO6.png]

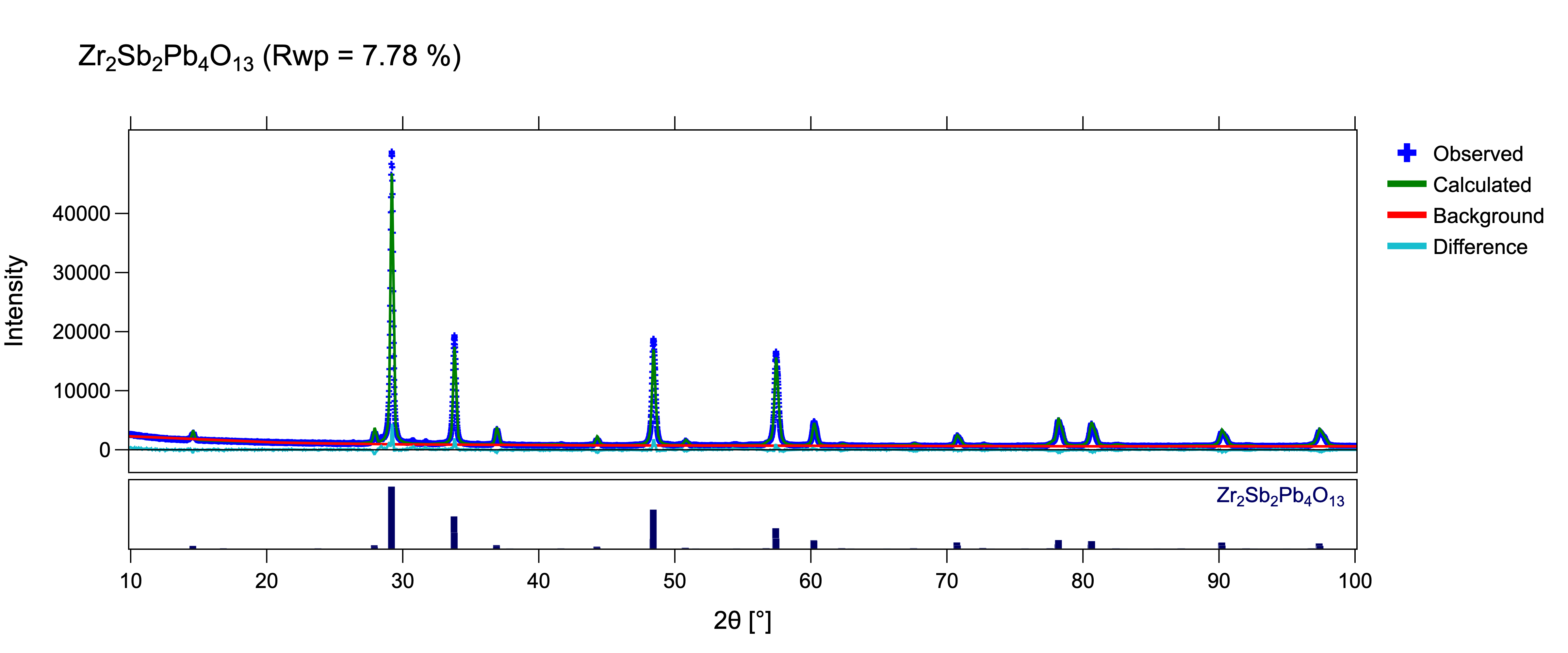

Supplement: Supplementary file 3 — This file contains the refined X-ray diffraction data from the successful syntheses performed by the A-Lab. The corresponding crystal structures used during refinement are also included in CIF format. [file 41586_2023_6734_MOESM3_ESM.zip › Manual_Refinement_Results/Zr2Sb2Pb4O13/Zr2Sb2Pb4O13.png]

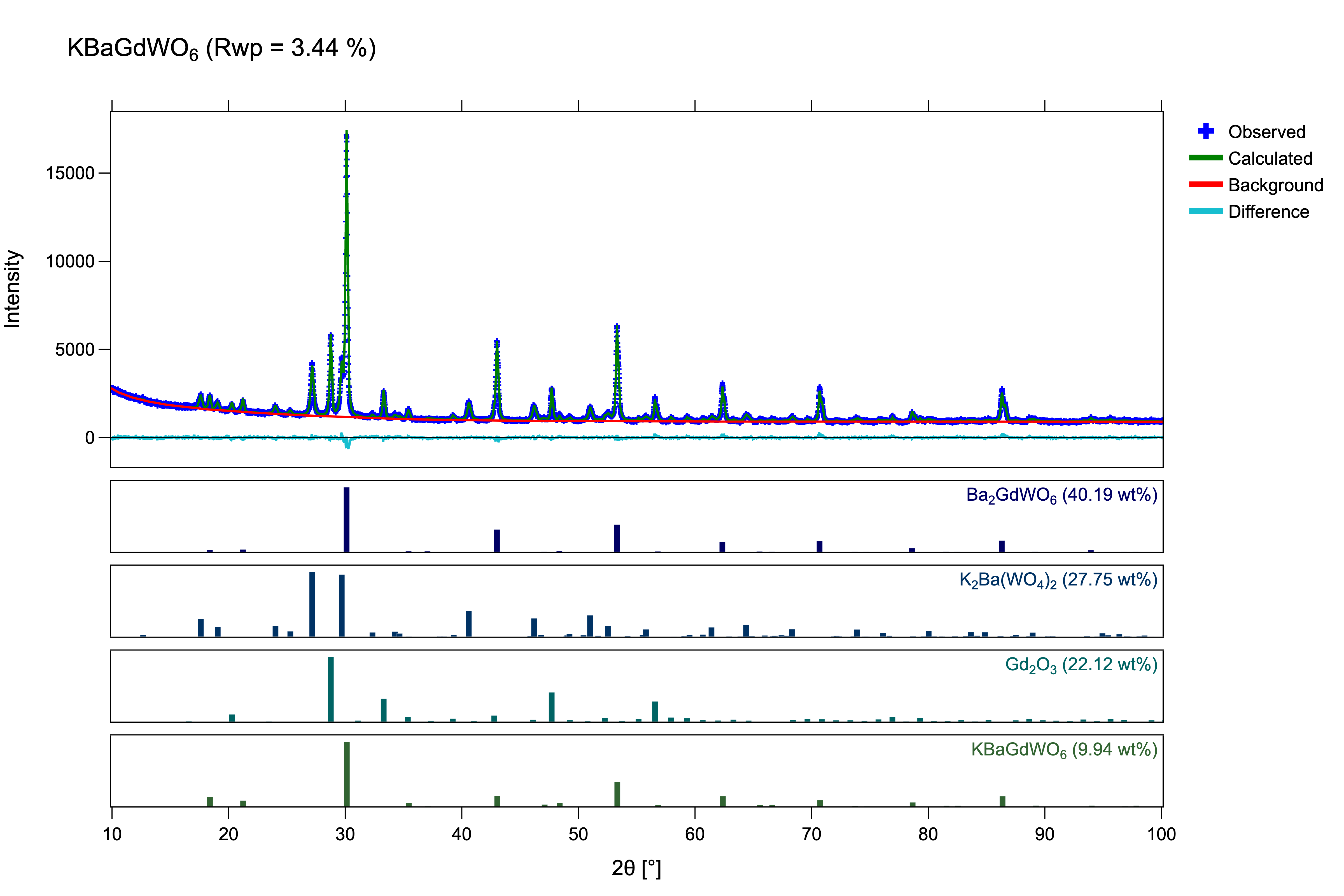

Supplement: Supplementary file 3 — This file contains the refined X-ray diffraction data from the successful syntheses performed by the A-Lab. The corresponding crystal structures used during refinement are also included in CIF format. [file 41586_2023_6734_MOESM3_ESM.zip › Manual_Refinement_Results/KBaGdWO6/KBaGdWO6.png]

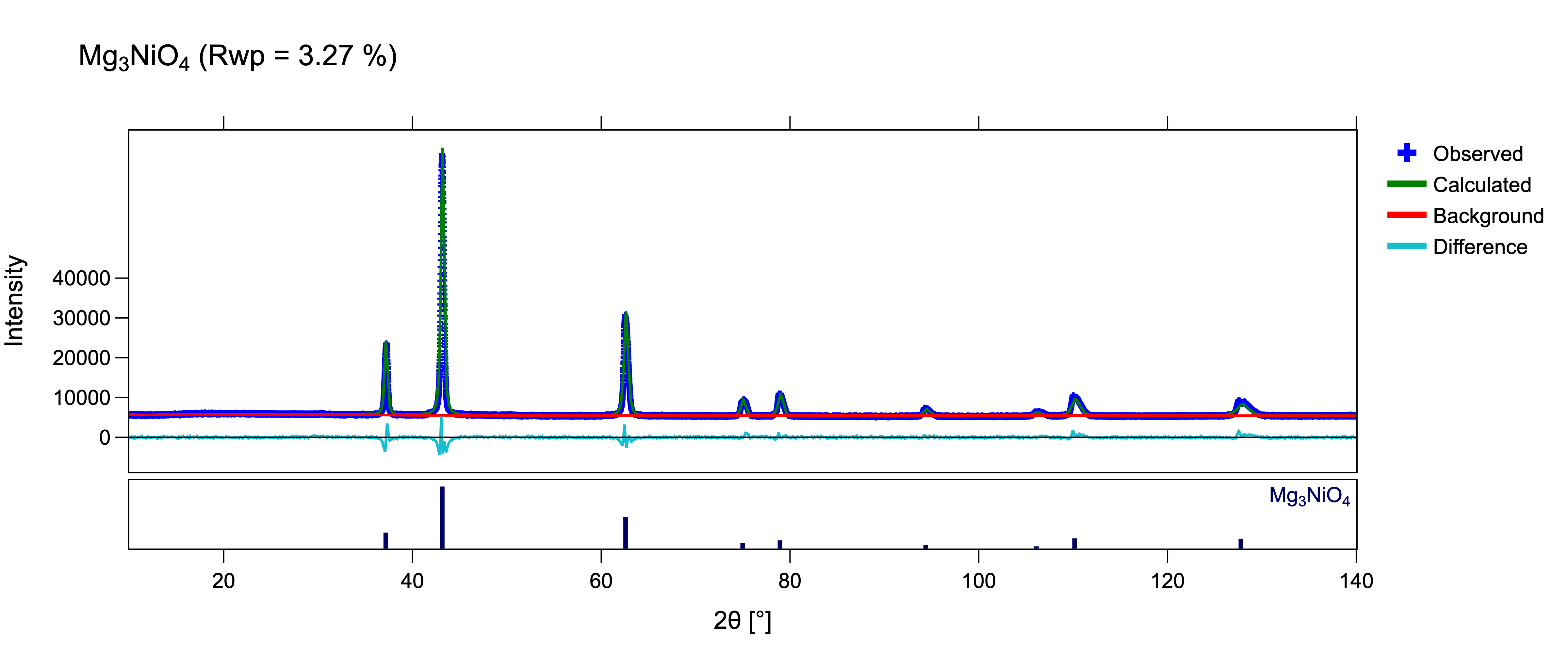

Supplement: Supplementary file 3 — This file contains the refined X-ray diffraction data from the successful syntheses performed by the A-Lab. The corresponding crystal structures used during refinement are also included in CIF format. [file 41586_2023_6734_MOESM3_ESM.zip › Manual_Refinement_Results/Mg3NiO4/Mg3NiO4.png]

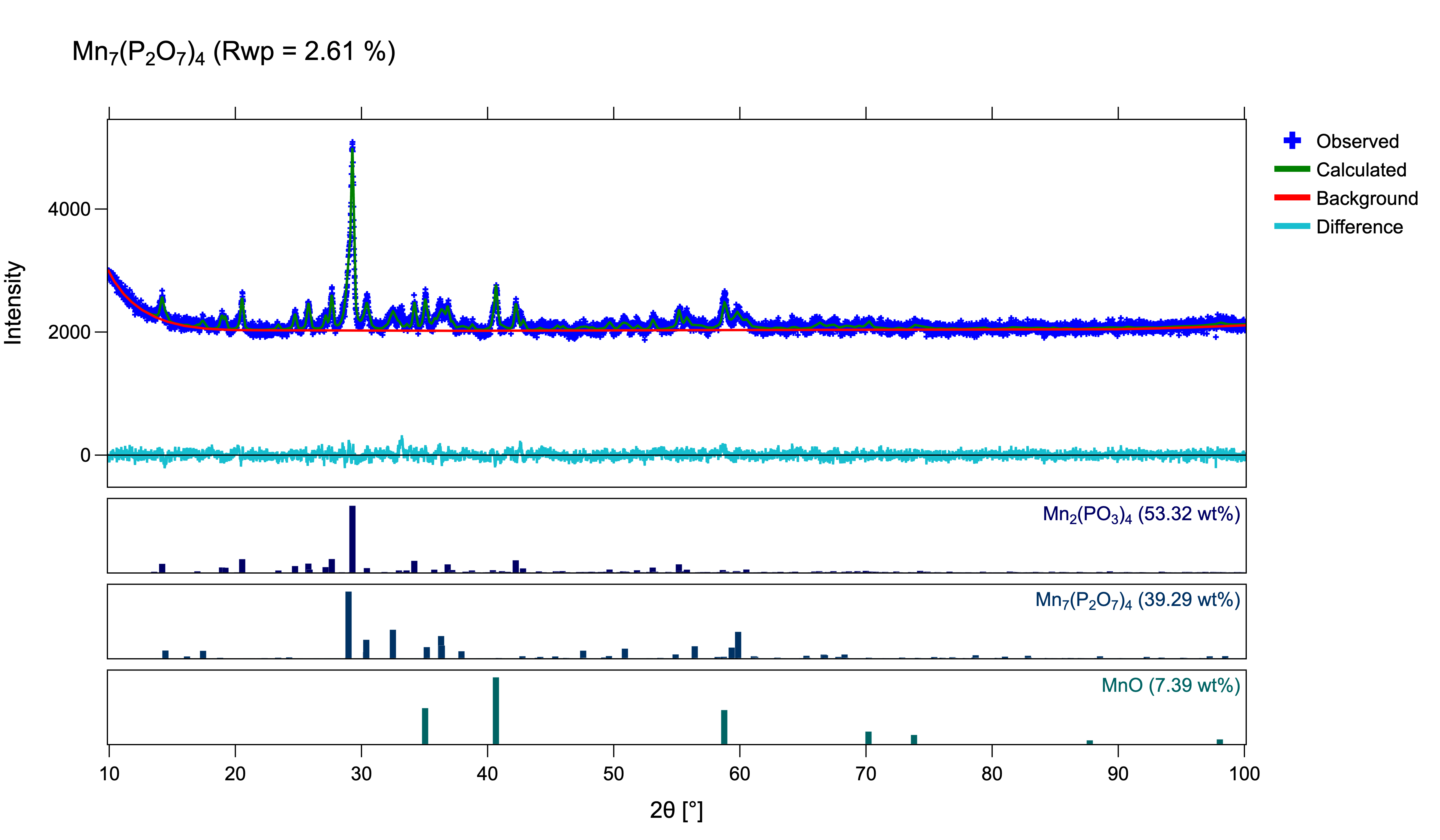

Supplement: Supplementary file 3 — This file contains the refined X-ray diffraction data from the successful syntheses performed by the A-Lab. The corresponding crystal structures used during refinement are also included in CIF format. [file 41586_2023_6734_MOESM3_ESM.zip › Manual_Refinement_Results/Mn7(P2O7)4/Mn7(P2O7)4.png]
