# Supplementary material for: Author Correction: An autonomous laboratory for the accelerated synthesis of inorganic materials
Source: Nature. 2026 Jan 19;650(8100):E1. doi: 10.1038/s41586-025-09992-y (PMC12872444; doi:10.1038/s41586-025-09992-y)
Supplement: Supplementary file 2 — Edits to Supplementary Information [file 41586_2025_9992_MOESM2_ESM.pdf]

nature portfolio

https://doi.org/10.1038/s41586-023-06734-w

Supplementary information

An autonomous laboratory for the accelerated synthesis of ~~novel~~ materials

In the format provided by the authors and unedited

Supplementary Information

An autonomous laboratory for the accelerated synthesis of ~~novel~~ materials

Nathan J. Szymanski[1,2,†], Bernardus Rendy[1,2,†], Yuxing Fei[1,2,†], Rishi E. Kumar[3,†], Tanjin He[1,2], David Milsted[2], Matthew J. McDermott[1,2], Max Gallant[1,2], Ekin Dogus Cubuk[4], Amil Merchant[4], Haegyeom Kim[2], Anubhav Jain[3], Christopher J. Bartel[2], Kristin Persson[1,2], Yan Zeng[2,*], and Gerbrand Ceder[1,2,*]

Table of contents

Supplementary Notes 1-11

Supplementary Figs. 1-6

Supplementary Tables 1-5

Supplementary Videos 1-3

Supplementary Note 1. Correction of DFT-calculated lattice parameters

Structures calculated using the PBE functional tend to underbind chemical bonds, resulting in unit cell volumes that are larger than experimentally observed. Indeed, comparing the volumes of 11,519 structures with matching entries from the Materials Project and the ICSD, the DFT-calculated structures overestimate the volumes of their experimental counterparts by 4.32%, on average. This volume difference can have a substantial effect on the positions of each phase's diffraction peaks, thus complicating phase identification based on XRD. Accordingly, a lattice correction is necessary before DFT-calculated structures can be included in the training set for our phase identification algorithm, XRD-AutoAnalyzer.

To machine learn an appropriate lattice correction for each structure, we use a mixture of graph neural network models with four different architectures: E3NN[1], GATGNN[2], CrysXPP[3], and ElemNet[4]. Each network was separately trained based on 9,330 structures (~80% of the total dataset available), which were randomly selected with replacement (bootstrap). A dropout rate of 75% was implemented for regularization. Once the models were finished training, we also employed a Monte Carlo dropout approach at inference, whereby 75% of the connections were randomly excluded from each network during its forward pass. A total of 150 passes were applied for each network, and their predictions were averaged. We further averaged the predictions from each distinct architecture to obtain a final prediction, which led to significant error reduction. On average, the volume error on the test set of 1,152 structures was reduced from 4.02% (before correction) to just 1.78% (post correction).

Supplementary Note 2. Automated Rietveld refinement

After obtaining phase predictions from XRD-AutoAnalyzer, we determine their weight fractions and associated parameters by using a closed-loop reinforcement learning (RL) approach to Rietveld refinement. To this end, we developed a Python workflow that wraps the GSAS-II refinement engine[5] in a custom gym environment. It can refine parameters or reset them to their initial values based on decisions made by the RL agent. The environment automatically resets certain parameters should they exceed a predefined set of bounds. For example, if the lattice parameters change by more than ±10%, the environment automatically resets them to their initial values. The parameters included during the refinement process are sample displacement, lattice parameter, particle size, isotropic microstrain, and phase fractions. After each step in the refinement, the experimental and calculated XRD patterns are provided to the RL agent to inform its next decision (*i.e.*, which parameter to change and by how much). The RL agent was trained with the objective to achieve minimal R_{wp} while using as few refinement steps as possible. As such, the agent is given a positive reward by reducing R_{wp}, and it is penalized if it either 1) received an error state that crashes the GSAS-II refinement engine, or 2) required too many refinement steps without improving R_{wp}.

An actor-critic algorithm is used to train our agent, where both actor and critic networks consist of two hidden layers, each with 64 neurons. Training is carried out using the proximal policy optimization (PPO) algorithm[6] as implemented in RLLIB[7]. The PPO parameters used are 5×10^{-5} learning rate, $\lambda_{GAE} = 0.95$, $\gamma = 0.99$, $\epsilon = 0.2$, initial $\beta = 0.2$, $d_{target} = 0.01$, and $c_2 = 1.0$. The dataset used for training includes eight XRD patterns measured from separately synthesized Li_2MnO_3 samples. We used a batch size of 1024 with 64 stochastic gradient descent minibatch size, and 10 training steps for each pattern, resulting in a total of ~85000 environment steps. We evaluated the trained policy on three separately prepared two-phase mixtures of Li_2CO_3 and MnO_2. The agent recovered reasonably accurate fits with weight fractions matching the expected values of 56% and 44% for Li_2CO_3 and MnO_2, respectively.

Supplementary Note 3. Literature-inspired synthesis recipe generation for $MgNi(PO_3)_4$

To demonstrate the methods used to generate initial synthesis recipes for ~~novel~~ targets, we provide a detailed walkthrough of the process for one target, $MgNi(PO_3)_4$. Our literature-inspired recommendation engine generates the first recipe by proposing the most common precursors for Mg, Ni, and P in reported solid-state synthesis experiments: MgO, NiO, and $NH_4H_2PO_4$. Additional recipes are then generated using a similarity-based strategy[16], which operates under the assumption that highly similar target materials can be produced using the same (or related) precursors. By using the PrecursorSelector encoding model[16], we calculated and ranked the similarity between $MgNi(PO_3)_4$ and each of the 28,598 target materials contained in our database of 33,343 solid-state synthesis procedures extracted from 24,304 publications. $BaMg_2(PO_4)_2$ was identified as the target material that is most similar to $MgNi(PO_3)_4$, and which is also synthesized using precursors different from the most common ones. The precursors used to synthesize $BaMg_2(PO_4)_2$ include $BaCO_3$, MgO, and $(NH_4)_2HPO_4$. A masked precursor completion (MPC) model[16] was then used to exchange the necessary elements (Ni/Ba) to reach our current target, $MgNi(PO_3)_4$. This algorithm identified NiO as the most likely replacement for $BaCO_3$, resulting in the following set of precursors: NiO, MgO, and $(NH_4)_2HPO_4$.

Following the process outlined above, three more recipes are generated by referring to three less similar targets, $Ca_8LuMg(PO_4)_7$, $Mg_{1.9}Ni_{0.1}TiO_4$, and Y_2MoO_6. The corresponding synthesis recipes are listed in Supplementary Table 3. For each set of precursors, the synthesis temperature was predicted using a pre-trained XGBoost regressor[17] based on the composition and thermodynamic properties of $MgNi(PO_3)_4$ and its precursors. Although the predicted temperature may vary for each recipe, we chose to use one fixed temperature for each target to maximize the possibility of batching multiple precursor sets in a single furnace. The actual synthesis temperature for $MgNi(PO_3)_4$ was calculated by averaging the five temperatures proposed across these recipes and rounded to the nearest hundred (900 °C).

Supplementary Note 4. Identification of unique synthesis pathways

The number of experiments required to exhaustively sample each target's search space, which consists of various precursor combinations and synthesis temperatures, can be substantially reduced by avoiding "redundant" reaction pathways. We consider the pathway for a given precursor set to be redundant if it forms the same intermediates at low temperature (T_1) as those formed by another precursor set that has already been tested at all temperatures. Because their intermediate phases are identical at T_1, it can be inferred that their products formed at higher temperatures ($T > T_1$) will also be identical. We therefore do not sample such pathways at $T > T_1$.

The formation of redundant intermediates can also sometimes be predicted based on previously observed pairwise reactions, in which case no experiments are required for the associated precursor set. Here we assume a pairwise reaction will occur in a given precursor set if its observed onset temperature is less than the minimum temperature considered for that set. Further details on this process are given in previous work[8].

Consider the targeted synthesis of $CaTiNiP_2O_9$ as an example, for which we have the following precursors available:

Ca: CaO, $Ca(OH)_2$, and $CaCO_3$

Ti: TiO_2

Ni: NiO, $NiCO_3$, $Ni(OH)_2$

P: $(NH_4)H_2PO_4$, $(NH_4)_2HPO_4$

These can be combined to form 18 different precursor sets, each of which may be heated to four different synthesis temperatures extracted from our text-based models (800, 900, 1000, 1100 °C). This results in a search space consisting of 72 possible experiments. From the start, these can be reduced by skipping any precursor sets containing $Ca(OH)_2$, which was previously observed to decompose to CaO below 700 °C (Supplementary Table 2), effectively reducing the search space to 48 experiments. Of these, many do not require explicit testing as the A-Lab quickly finds that every precursor set collapses onto one of two distinct reaction pathways defined by the following sets of intermediates: 1) TiO_2, NiO, $CaTi_4(PO_4)_6$, and $Ca_2P_2O_7$; or 2) $CaNi_3(P_2O_7)_2$, $NiTiO_3$, and TiO_2. Both sets remain inert within the heating time allotted by the A-Lab, likely owing to their low driving force to react (< 40 meV/atom), thus exhausting the search space for $CaTiNiP_2O_9$.

Supplementary Note 5. Exhausting the search space for $BaGdCrFeO_6$ synthesis

The A-Lab's experimental campaign targeting $BaGdCrFeO_6$ began with four literature-inspired synthesis recipes that included the following precursor sets with a hold temperature of 1000 °C:

Set A: $BaCO_3$, Cr_2O_3, Fe_2O_3, Gd_2O_3

Set B: $BaCO_3$, Cr_2O_3, Fe_3O_4, Gd_2O_3

Set C: BaO, Cr_2O_3, Fe_2O_3, Gd_2O_3

Set D: BaO_2, Cr_2O_3, Fe_2O_3, Gd_2O_3

Sets A and **B** produced samples that predominantly contained $BaCrO_4$, in addition to minority amounts of $GdCrO_3$ and Fe_2O_3. In contrast, the samples resulting from **Sets C** and **D** contained substantially larger amounts of $GdCrO_3$ and only small amounts of $BaCrO_4$. These outcomes suggest some differences between the reaction pathway of each set. To further investigate these differences, the active learning algorithm (ARROWS[3]) implemented in the A-Lab proposed that all four precursor sets be tested at lower temperature (700 °C). The products obtained from **Sets A** and **B** contained $BaCrO_4$ in addition to Gd_2O_3 and Fe_2O_3. From these results, the algorithm gained information regarding two reactions:

$$4\ BaCO_3 + 2\ Cr_2O_3 + 3\ O_2 \rightarrow 4\ BaCrO_4 + 2\ CO_2\ (T_{rxn} \leq 700\ °C)$$

$$4\ Fe_3O_4 + O_2 \rightarrow 6\ Fe_2O_3\ (T_{rxn} \leq 700\ °C)$$

In contrast, the samples produced by **Sets C** and **D** at 700 °C predominantly contained unreacted precursors, with just minority amounts of $BaCrO_4$. The formation of $GdCrO_3$, while evident at 1000 °C for each set, was not yet initiated at a lower temperature of 700 °C. Accordingly, ARROWS[3] learned the following information from these outcomes:

$$4\ BaO + 2\ Cr_2O_3 + 3\ O_2 \rightarrow 4\ BaCrO_4\ (T_{rxn} \leq 700\ °C)$$

$$4\ BaO_2 + 2\ Cr_2O_3 + O_2 \rightarrow 4\ BaCrO_4\ (T_{rxn} \leq 700\ °C)$$

$$Gd_2O_3 + Cr_2O_3 \rightarrow 2\ GdCrO_3\ (700\ °C < T_{rxn} \leq 1000\ °C)$$

Because the four initially tested precursor sets were found to react and form $BaCrO_4$ at the lower bound of the temperature range considered (700-1000 °C), ARROWS[3] next suggested a set of precursors that was not yet observed to form such intermediates. This set also excludes Fe_3O_4 as the algorithm learned that it simply oxidizes to Fe_2O_3 at a temperature lower than 700 °C.

Set E: $Ba(OH)_2$, Cr_2O_3, Fe_2O_3, Gd_2O_3

However, when evaluated at 700 °C, this precursor combination was found to produce large amounts of $BaCrO_4$, appearing similar to the samples made by **Sets A** and **B**. The algorithm gleaned the following information from this outcome:

$$4\ Ba(OH)_2 + 2\ Cr_2O_3 + 3\ O_2 \rightarrow 4\ BaCrO_4 + 2\ H_2O\ (T_{rxn} \leq 700\ °C)$$

At this point, ARROWS[3] identified two unique reaction pathways characterized by the amount of $BaCrO_4$ observed at low temperature: **Sets A**, **B**, and **E** formed $BaCrO_4$ in majority amounts at 700 °C while **Sets C** and **D** produced only minority amounts of that phase and instead contained a larger weight fraction of unreacted precursors. To further investigate these synthesis routes, the algorithm selected one representative set (**D** and **E**) from each pathway to evaluate at 800 °C. In either case, the products made at this temperature appeared similar to those obtained at 1000 °C. **Set D** (**E**) contained large (small) amounts of $BaCrO_4$ and small (large) amounts of $GdCrO_3$. From these results, ARROWS[3] refined the temperature range in which $GdCrO_3$ forms:

$$Gd_2O_3 + Cr_2O_3 \rightarrow 2\ GdCrO_3\ (700\ °C < T_{rxn} \leq 800\ °C)$$

The algorithm also determined that there was no need to probe additional temperatures (900 °C) as the products formed at 800 °C appeared similar to those obtained at 1000 °C. At this point, all unique reaction pathways had been exhausted. As such, no more experiments were performed, and the synthesis was deemed “failed.”

Supplementary Note 6. Successful optimization of $CaFe_2P_2O_9$ synthesis

The A-Lab's experimental campaign targeting $CaFe_2P_2O_9$ began with four synthesis recipes that were suggested by the literature-inspired machine learning algorithms. These include the following precursor sets, each evaluated at 1100 °C:

Set A: $CaCO_3$, Fe_2O_3, $(NH_4)_2HPO_4$

Set B: CaO, Fe_2O_3, $(NH_4)_2HPO_4$

Set C: $Ca(OH)_2$, Fe_2O_3, $(NH_4)_2HPO_4$

Set D: CaO, Fe_3O_4, $NH_4H_2PO_4$

These produced glassy samples with low target yield and poor signal-to-noise ratio, likely due to melting at high temperature and subsequent amorphization upon cooling. As such, the same precursor sets were evaluated at lower temperature (800 °C). They were all found to produce identical phases: $Ca_3(PO_4)_2$ and $FePO_4$. From these results, the active learning algorithm (ARROWS[3]) learned five pairwise reactions that occurred ≤ 800 °C, each listed below:

$$3\ CaCO_3 + 2\ (NH_4)_2HPO_4 \rightarrow Ca_3(PO_4)_2 + 4\ NH_3 + 3\ H_2O + CO_2$$

$$3\ Ca(OH)_2 + 2\ (NH_4)_2HPO_4 \rightarrow Ca_3(PO_4)_2 + 4\ NH_3 + 6\ H_2O$$

$$3\ CaO + 2\ NH_4H_2PO_4 \rightarrow Ca_3(PO_4)_2 + 2\ NH_3 + 3\ H_2O$$

$$Fe_2O_3 + 2\ (NH_4)_2HPO_4 \rightarrow 2\ FePO_4 + 4\ NH_3 + 3\ H_2O$$

$$4\ Fe_3O_4 + 12\ NH_4H_2PO_4 + O_2 \rightarrow 12\ FePO_4 + 12\ NH_3 + 18\ H_2O$$

Using its interface with the Materials Project, the algorithm also determined that there is little thermodynamic driving force (8 meV/atom) remaining to form the target when $Ca_3(PO_4)_2$ and $FePO_4$ precede it. As such, it proposed two new sets of precursors to be tested at 800 °C:

Set E: CaO, Fe_2O_3, $NH_4H_2PO_4$

Set F: CaO, Fe_3O_4, $(NH_4)_2HPO_4$

In each set, ARROWS[3] predicted a reaction between CaO and the phosphate precursor to form $Ca_3(PO_4)_2$ at a temperature ≤ 800 °C. However, the algorithm did not predict the formation of $FePO_4$ in either set as the below precursor pairs were not yet tested:

$Fe_2O_3|NH_4H_2PO_4$ and $Fe_3O_4|(NH_4)_2HPO_4$

After evaluating both precursor sets at 800 °C, it was found that **Set E** followed a similar reaction pathway as **Sets A-D**. However, **Set F** was clearly distinguished by the formation of new peaks in

its corresponding XRD pattern that could not be attributed to any known phases reported in the Materials Project or the ICSD.

Based on the first six experimental outcomes, ARROWS[3] identified two different reaction pathways, one originating from **Sets A-E**, and another originating from **Set F**. As detailed in Supplementary Note 4, each unique reaction pathway only needs to be probed once throughout the full range of temperatures to determine whether it will successfully lead to the target's formation. Accordingly, the algorithm chose one precursor set from the first pathway (**Set E**) and another from the second (**Set F**) to be evaluated at higher temperature (900 °C). While the products from **Set E** exhibit little change from those observed at 800 °C, it was found that **Set F** produced a new phase, $CaFe_3P_3O_{13}$. This phase is computed to have a large driving force (77 meV/atom) to react with CaO and form the target. Indeed, heating **Set F** at 1000 °C led to the formation of $CaFe_2P_2O_9$ with a yield of 75%. The experimental campaign was therefore halted as the results satisfied our stopping criterion of > 50% target yield.

Supplementary Note 7. Synthesis modifications to overcome slow reaction kinetics

To overcome the slow kinetics associated with reactions that have a low thermodynamic driving force, we used two common strategies[9]: 1) heating the precursor set to a higher synthesis temperature than originally used, and 2) regrinding the synthesis products and reheating them to their original synthesis temperature for a longer hold time. The first method was used in six cases when the original (literature-inspired) synthesis temperature was lower than 1000 °C, and therefore increased temperature was accessible with a standard box furnace. The second method was used in five cases, when the temperature was already high (≥ 1000 °C). The targets attempted using each approach and their corresponding outcomes are listed in Supplementary Table 4.

For synthesis experiments that were repeated at higher temperatures, the same mixing protocol was used to prepare the samples. The heating rate was also kept the same, with the only difference being the increased dwell temperature. Of the six targets that we attempted at higher temperatures, one additional compound ($Y_3Ga_3In_2O_{12}$) was successfully obtained with high purity.

For the samples that were subject to regrinding and reheating, the original synthesis products were first manually ground in an agate mortar, followed by wet grinding in a Dual Asymmetric Centrifuge mixer for 10 minutes with six 5 mm ZrO_2 balls and ethanol. The resulting slurry was then dried and heated in a box furnace with the same heating profile as used previously (Methods). Of the five targets that were attempted with this strategy, one additional compound (Mg_3NiO_4) was successfully obtained with high purity.

Supplementary Note 8. Characterization of precursor volatility

To quantify the suspected loss of the ammonium phosphate precursors during the synthesis experiments targeting $CaCr_2P_2O_9$, we used energy-dispersive X-ray spectroscopy (EDS) to analyze the elemental composition in of its synthesis product that was made by holding $CaCO_3$, Cr_2O_3, and $NH_4H_2PO_4$ at 900 °C for 4 h. The resulting EDS mapping is shown in Supplementary Fig. ~~6~~, which reveals that the atomic ratio of phosphorus to the metals (Ca/Cr) is only 0.37, being much lower than expected given the starting ratio based on the precursors (0.66). This result suggests that some of the phosphate ions were successfully trapped by the formation of calcium phosphate, which was also detected in XRD, but the remaining became volatile during the heating process. The only other phase detected was an oxide, Cr_2O_3, which appeared not to react with the phosphate prior to its volatility.

Supplementary Note 9. Assessing the novelty of target materials

We began this work by selecting 58 targets for synthesis by the A-Lab (Methods). Given that these targets were not present in the ICSD or Synterra, we considered them to be previously unreported. However, once a target material was successfully synthesized, we conducted a more thorough manual investigation to verify whether it has indeed been previously synthesized. This level of scrutiny was difficult to apply at scale during initial target screening, but critical when disseminating our results.

Upon closer examination, we discovered that six of the 58 targets were, in fact, reported as experimentally obtained yet not present in our reference databases. Many of these were reported in a fashion that was difficult to query (*e.g.*, as impurity phases for a different synthesis or one of many compounds in a study of elemental substitution). We have listed these targets along with their corresponding sources in Supplementary Table 5. Two additional targets (Mg_3NiO_4 and $YbMoO_4$) are mentioned in previous reports[10,11] as components of multi-phase reaction products but without conclusive structural characterization, so we consider these to be newly confirmed by our experiments. To the best of our knowledge, all remaining (52/58) compounds are previously unreported.

Supplementary Note 10. Specifications of robots in the A-Lab

The A-Lab contains three 6-axis robot arms: one Mitsubishi RV7-FL and two Universal Robots (UR5e), each with force sensors and bespoke grippers (Extended Data Fig. 2a-c) designed to accommodate different types of consumables. Here we refer to the Mitsubishi robot as R1, the first UR5e robot as R2, and the second UR5e robot as R3. The Mitsubishi and UR5e robots can handle maximum payloads of 7 kg and 5 kg, respectively, excluding the tools and grippers mounted on them. The precision of each robot is within ±0.02 and ±0.03 mm, respectively. R1 uses two custom grippers to interact with the Quantos powder dosing heads, crucibles, and plastic vials/mixing pots (Supplementary Video 1). The grip is controlled using pneumatic actuators, regulated by pressure to achieve the specified force setting. R2 uses a Robotiq Hand-E gripper to handle crucibles and ceramic racks (Supplementary Video 2). R3 uses a Robotiq 2F-85 gripper to handle plastic vials, crucibles, acrylic discs, and XRD sample holders (Supplementary Video 3). Both UR5e robots grippers force can be adjusted, controlled by electric motors. This mechanism is convenient for programming delicate sample transfers, especially for strong but brittle alumina crucibles. R1 has a reach of 908 mm while R2 and R3 have a reach of 850 mm, before being extended by the grippers.

R1 and R3 are each mounted on stationary platforms, while R2 is mounted on a linear rail so that it can transfer samples from the precursor preparation station to the box furnaces. This linear rail was designed and installed in coordination with Olympus Controls. The rail uses a 4000 mm belt-driven HMRB15CCD0-4000-CD500K100 from Parker with a precision of ±0.05 mm. Limit sensors are employed on both ends of the rail for recalibration (Extended Data Fig. 2d). The belt is driven by 3:1 PV60TA-003 (Parker) gearbox connected to LMDCE853C Novanta IMS Lexium stepper motor with ethernet connection that directly interfaces with UR teach pendant through URCap program provided by Schwarz Automation Inc. The aluminum platform to mount the linear rail is designed and manufactured by Olympus Controls. The sub-millimeter repeatability and direct power connection to the wall allows for little-to-no downtime required for maintenance and recalibration. While a linear rail only extends the work envelope in one axis, it also ensures improved repeatability relative to a system with increased degrees of freedom.

A carousel with four quadrants and a light gate is used in the precursor preparation station to arrange samples and place them for collection by the first UR5e robot (R2), as depicted in

Extended Data Fig. 2e. For most operations, the carousel moves in coordination with the robot (R1) that prepares samples prior to heating. However, R2 can also request control when it needs to collect the samples and transfer them to the box furnaces. Once control is given, the carousel is directed to rotate such that the proper quadrant faces R2 and the samples are accessible from the robot's side (Supplementary Video 2). The use of this carousel ensures that many samples can be made and stored simultaneously in the precursor preparation station, allowing some queue to be developed prior to the heating step.

Supplementary Note 11. Exceptions to pairwise reaction analysis

The active learning algorithm (ARROWS[3]) implemented in the A-Lab learns from synthesis outcomes by determining which pairwise reactions led to the formation of any newly observed products. From previous studies that utilized *in situ* XRD[12-14], there is an abundance of evidence supporting the idea that solid phases generally react in pairs. Such reactions occur locally at the interfaces between precursors, where little diffusion is needed to form the resulting product. However, there are likely to be exceptions to this rule when precursors or reaction intermediates deviate from the solid state. For example, we anticipate that reactions may take place between more than two phases at a time when melting occurs. This would enable atoms to travel more freely throughout the sample, thereby alleviating the requirement that two phases react locally at an interface. Indeed, recent work has shown that reactions proceed in a non-pairwise sequence when a multi-component oxide forms a eutectic melt[15].

Pairwise reaction analysis can also be complicated by difficulties in characterization. For example, as more precursors are introduced to synthesize targets with many components, it may be challenging to interpret their XRD patterns owing to substantial peak overlap. Such cases may therefore warrant the use of additional characterization techniques that can spatially resolve distinct phases (*e.g.*, SEM/EDS measurements). We note that even in cases where pairwise reaction analysis is insufficient, or where the synthesis products are not completely characterized, the active learning algorithm will continue to operate. It will simply do so with reduced efficiency as it may not learn which precursors contribute to the formation of a desired synthesis product or its competing phases.

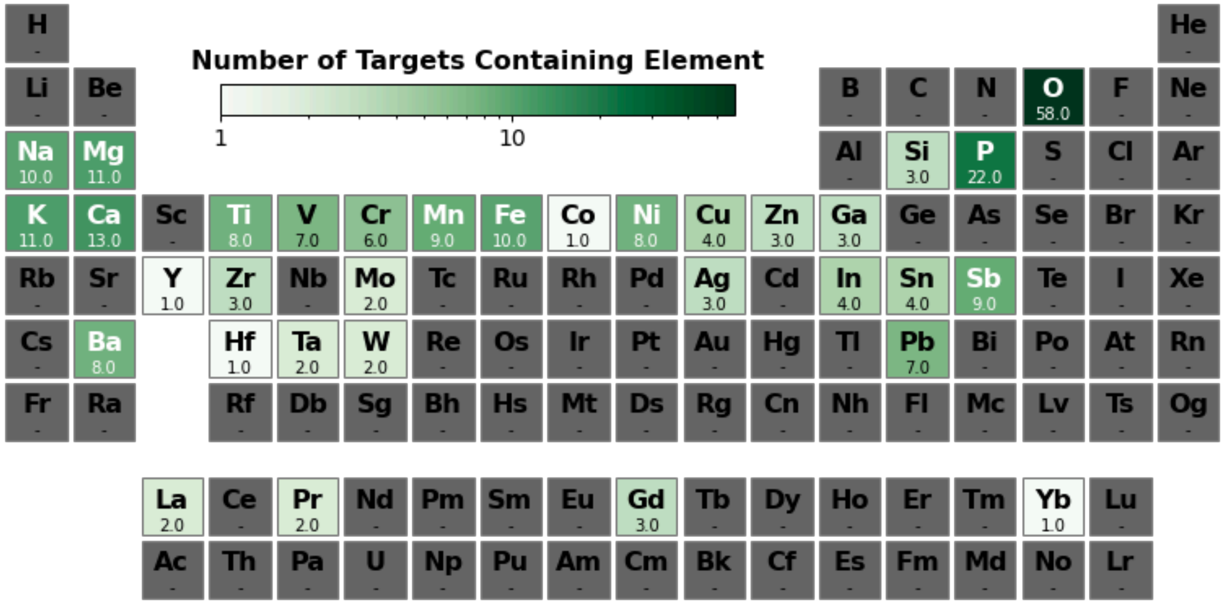

Supplementary Fig. 1 | Frequency of elements across the A-Lab's 58 target materials. The periodic table above shows the frequency at which each of 33 elements appears in the compounds targeted by the A-Lab.

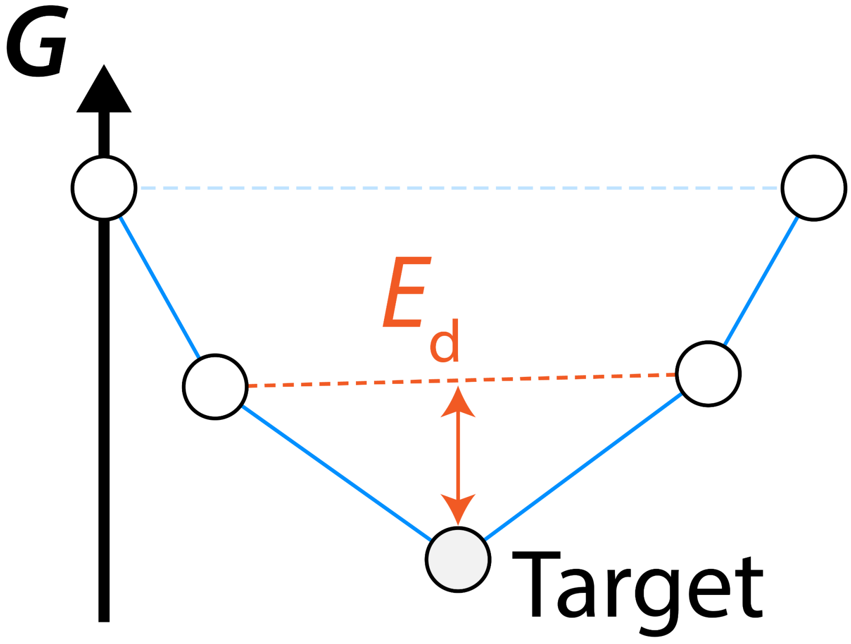

Supplementary Fig. 2 | Schematic illustration of the decomposition energy. A binary convex hull is shown, where each circle represents a distinct phase that is thermodynamically stable. The y-axis represents the Gibbs free energy (G) while the x-axis represents a composition parameter. The decomposition energy (E_d) of a given target phase is determined by calculating the distance between its energy and the energy of a tieline formed by the two neighboring phases along the convex hull.

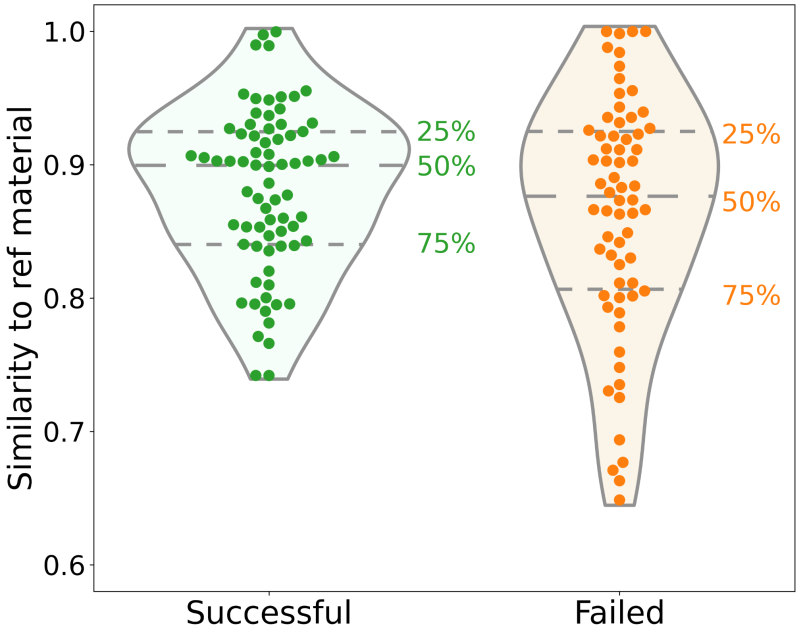

Supplementary Fig. 3 | Successes and failures of literature-inspired recipes. For each target material, up to five recipes are recommended as summarized in the Methods. The distribution of similarity – defined in previous work[16] – to the reference material for recipe recommendation is categorized by successful and failed syntheses. Each colored dot represents one recipe generated through the similarity-based recommendation. The width in each violin plot reflects the probability density of the number of recipes generated at different similarity values. The quartiles (25%, 50%, and 75%) are displayed to indicate the different statistics between successful and unsuccessful recommendation

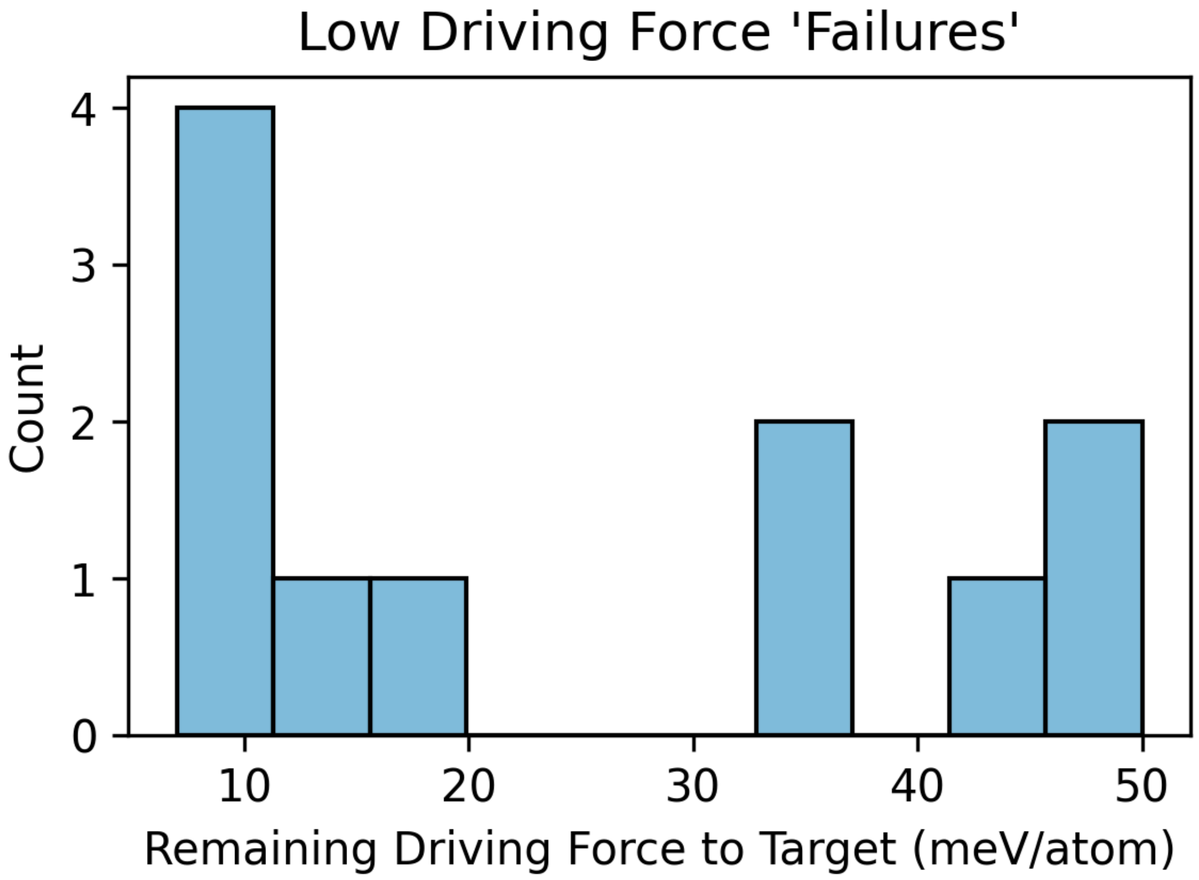

Supplementary Fig. 4 | Reactions limited by low driving force. For eleven of the failed syntheses, the reaction energy that remains after intermediates are formed (or directly from the precursors) was below 50 meV/atom. These values were determined by calculating the free energy difference between the observed intermediates and the desired target. Exact values are also given in Supplementary Table 4.

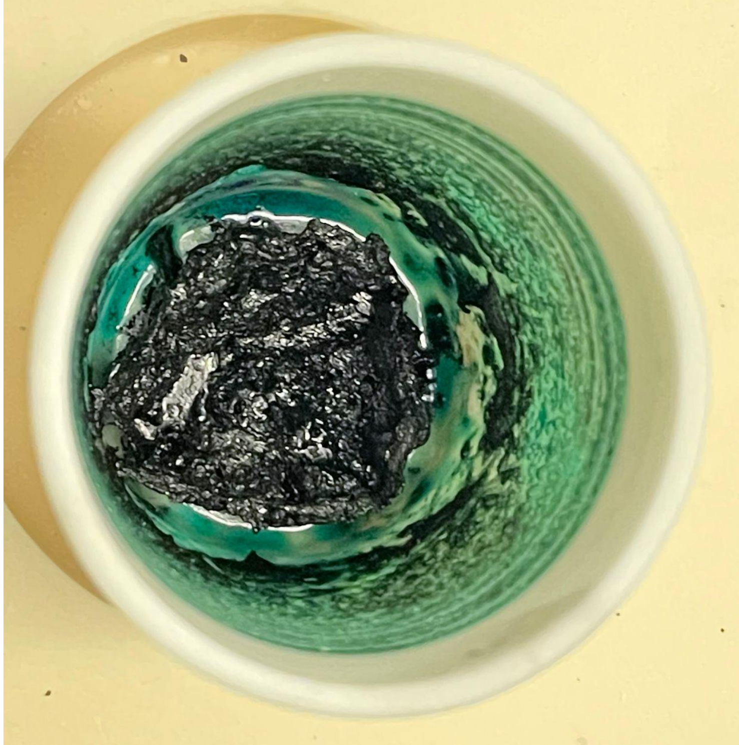

Supplementary Fig. 5 | Example of an amorphous synthesis product. The sample imaged above was obtained from an attempted synthesis of $Mo(PO_3)_5$ based on a precursor mixture of MoO_3 and $(NH_4)_2HPO_4$ annealed at 500 °C for 4 h, followed by a cooling rate of 2 °C/min.

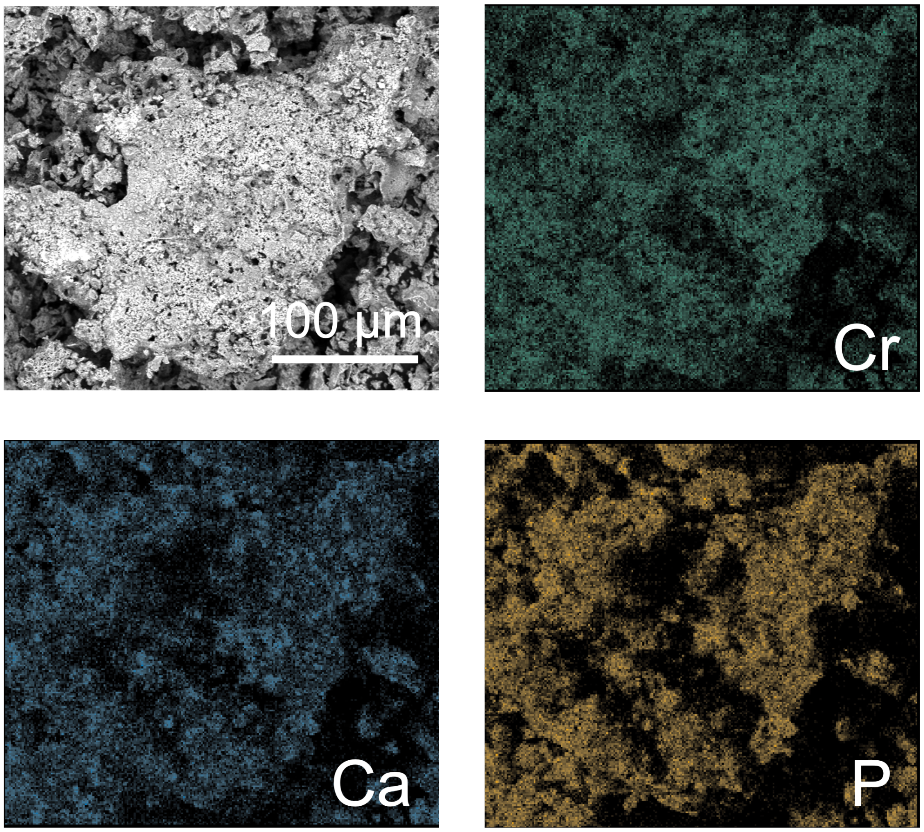

Supplementary Fig. 6 | SEM/EDS mapping of the synthesis product targeting $CaCr_2P_2O_9$. These measurements were taken from a sample made using $CaCO_3$, Cr_2O_3, and $NH_4H_2PO_4$ held at 300 °C for 6 hours, and then sintered at 900 °C for 4 hours.

Supplementary Table 1 | All 58 targets evaluated by the A-Lab, grouped into 41 unique structural prototypes with space groups listed. When a similar reference compound is available, we include it as a corresponding ICSD prototype. Materials Project (MP) entry ID numbers are listed for each target.

Group #	ICSD Prototype	Space group	Target	MP Entry #
1	Ni2(PO3)4	C2/c	MgNi(PO3)4	mp-1045786
			CaMn(PO3)4	mp-1045779
			CaNi(PO3)4	mp-1045813
			CaCo(PO3)4	mp-1045787
2	Pyrochlore(defect)#Gd1.8 Ti2O6+x	Imm2	Hf2Sb2Pb4O13	mp-1224490
			Zr2Sb2Pb4O13	mp-1215826
			Sn2Sb2Pb4O13	mp-1219056
		R3m	FeSb3Pb4O13	mp-1224890
			InSb3Pb4O13	mp-1223746
3	Garnet#Ca3Al2(SiO4)3	Ia-3d	Ca3Ti3Cr2O12	mp-1214263
			Na3V3Cr2O12	mp-1202673
			Y3In2Ga3O12	mp-1207946
4	Perovskite(12H)#Ba6Nd2 Ti4O17	F-43m	KBaPrWO6	mp-1523149
			BaGdCrFeO6	mp-1518724
			KBaGdWO6	mp-1523079

		P6_3/mmc	Ba6Na2Ta2V2O17	mp-1214664
			Ba6Na2V2Sb2O17	mp-1214658
5	Spinel#MgAl2O4	C2/c	Zn3Ni4(SbO6)2	mp-1216023
			Mn4Zn3(NiO6)2	mp-1222033
6	Cd2Mn3O8	C2/m	Ca2Ti3O8	mp-1397126
			Ca2Sn3O8	mp-1386610
7	CdP2V2O9	Pnma	CaFe2P2O9	mp-1040941
			CaCr2P2O9	mp-1040889
8	Ba6Nd2Al4O15	P1	Ba9Ca3La4(Fe4O15)2	mp-1228537
9	Perovskite#LaAlO3	P1	La5Mn5O16	mp-531717
10	Alluaudite-frame#Na1.5Mn2.5Al(PO4)3	P1	Na7Mg7Fe5(PO4)12	mp-1173791
11	Fe7(P2O7)4	C222_1	Mn7(P2O7)4	mp-778008
12	Na2CuP2O7#Na2PdP2O7	C2/c	CuAg2P2O7	mp-1213198
13	Beryllonite#NaBePO4#SrGa2O4	P2_1/c	KNa2Ga3(SiO4)3	mp-1211711
14		R-3m	Zn2Cr3FeO8	mp-1215741
15	Whitlockite#β-Ca3(PO4)2	P1	Na3Ca18Fe(PO4)14	mp-725491
16	Mg6MnO8	R-3m	Mg3MnNi3O8	mp-1222170
17	NaCl	Pm-3m	Mg3NiO4	mp-1099253
18	K2Cr2O7(H_type)	P1	MgV4Cu3O14	mp-1222158
19	KTiOPO4#KFePO4F	P2_1	K4TiSn3(PO5)4	mp-1224290
20	K4Fe4(PO4)5	Cc	K4MgFe3(PO4)5	mp-532755

21		C2/c	KMn3O6	mp-1016190
22	Fe2(SO4)3	Pc	InSb3(PO4)6	mp-1224667
23	LiV3O8	P2_1/m	V3AgO8	mp-1100976
24	CaTa4O11	R32	Ta4PbO11	mp-1194594
25	CdZr4(PO4)6	R-3	MgTi4(PO4)6	mp-1222070
26		P2_1/m	Mo(PO3)5	mp-504210
27		P3	KPr9(Si3O13)2	mp-1223421
28	NaNiO2(mS8)	C2/m	MnAgO2	mp-996995
29	Langbeinite#K2Mg2(SO4)3	Pna2_1	KNaTi2(PO5)2	mp-1211611
		P2_13	K2TiCr(PO4)3	mp-1224541
30	Garnet(YAG)#Y3Al5O12	P-1	CaGd2Zr(GaO3)4	mp-686296
31	Ilmenite#TiFeO3	R3	MgTi2NiO6	mp-1221952
32	Double_Perovskite#Elpasolite#K2NaAlF6	Fm-3m	Ba2ZrSnO6	mp-1228067
33	Sc2Si2O7	Cm	Mn2VPO7	mp-1210613
34	CaMnP2O7	P-1	MgCuP2O7	mp-1041741
35	KMgCu4V3O13	C2/c	KMg3V3CuO12	mp-1211434
36	Scheelite#CaWO4	I4_1/a	YbMoO4	mp-1207551
37	K2NiF4	Cmmm	Ba4InSbO8	mp-1228129
38	Er3Pb1.5(SiO4)3	P3	KNaP6(PbO3)8	mp-1223429
39	Olivine#Mg2SiO4	P-1	NaMnFe(PO4)2	mp-1173592
40	CdP2V2O9	Pnma	CaTiNiP2O9	mp-1043092
41		C2	NaCaMgFe(SiO3)4	mp-1221075

Supplementary Table 2 | All pairwise reactions autonomously learned by the A-Lab during its active learning cycle.

Reactants	Products	Onset Temperature (°C)
Ca(OH)2|NH4H2PO4	Ca3(PO4)2	≤ 800
Ca(OH)2|(NH4)2HPO4	Ca3(PO4)2	≤ 800
CaCO3|(NH4)2HPO4	Ca3(PO4)2	≤ 800
CaCO3|NH4H2PO4	Ca3(PO4)2	≤ 800
CaO|NH4H2PO4	Ca3(PO4)2	≤ 800
CaO|(NH4)2HPO4	Ca3(PO4)2	≤ 800
CaO|TiO2	CaTiO3	≤ 800
Ca(OH)2|TiO2	CaTiO3	≤ 800
CaCO3|TiO2	CaTiO3	≤ 800
Fe2O3|(NH4)2HPO4	FePO4	≤ 800
Fe3O4|NH4H2PO4	FePO4	≤ 800
CaFe3P3O13|CaO	CaFe2P2O9	900 < T ≤ 1000
Na2CO3|FePO4	NaFeP2O7	≤ 700
MgO|NiO	MgNiO2	800 < T ≤ 900
MgCO3|NiO	MgNiO2	≤ 900
MgCO3|air	MgO	≤ 700
Ni(OH)2|air	NiO	≤ 700
MnO|(NH4)2HPO4	Mn2P4O12	≤ 500
MnO|NH4H2PO4	Mn2P4O12	≤ 500
MnO2|NH4H2PO4	Mn2P4O12	≤ 500
MnCO3|NH4H2PO4	Mn2P4O12	≤ 500
Mn2O3|(NH4)2HPO4	Mn2P4O12	≤ 500
Mn3O4|NH4H2PO4	Mn2P4O12	≤ 500
Mn3O4|(NH4)2HPO4	Mn2P4O12	≤ 500
MnO|air	Mn2O3	≤ 600
MnCO3|air	Mn2O3	≤ 600
Mn3O4|air	Mn2O3	≤ 600

MnO2|air	Mn2O3	≤ 600
MgCO3|air	MgO	≤ 600
TiO2|NiO	NiTiO3	800 < T ≤ 900
Y2(CO3)3|Fe2O3	YFeO3	≤ 900
Y2O3|Fe2O3	YFeO3	≤ 900
MoO2|(NH4)2HPO4	Mo(PO3)3, Mo2P4O15	≤ 900
MoO2|NH4H4PO4	Mo(PO3)3, Mo2P4O15	≤ 900
MoO3|(NH4)2HPO4	Mo(PO3)3, Mo2P4O15	≤ 900
MoO3|NH4H4PO4	Mo(PO3)3, Mo2P4O15	≤ 900
CuO|(NH4)2HPO4	Cu2P2O7	≤ 900
NiO|Sb2O5	NiSb2O6	≤ 900
Ni(OH)2|Sb2O5	NiSb2O6	≤ 900
ZnO|NiO	ZnNiO2	≤ 900
Sb2O5|air	SbO2	≤ 900
Sb2O3|air	SbO2	≤ 600
Ag2CO3|air	Ag	≤ 700
La2O3|Mn3O4	LaMnO3	≤ 1000
MgO|V2O5	Mg3V2O8	≤ 900
MgCO3|V2O5	Mg3V2O8	≤ 900
Y2(CO3)3|air	Y2O3	≤ 600
Fe3O4|air	Fe2O3	≤ 600
CaCO3|SnO2	CaSnO3	≤ 800
Ca(OH)2|SnO2	CaSnO3	≤ 800
Na2CO3|V2O5	NaVO3	≤ 500
CuO|Ta2O5	CuTa2O6	≤ 800
Ag2CO3|NH4H2PO4	Ag3PO4	≤ 600
Ag2CO3|(NH4)2HPO4	Ag3PO4	≤ 600
Sb2O3|NH4H2PO4	SbPO4	≤ 600
Sb2O5|NH4H2PO4	SbPO4	≤ 600
Sb2O3|(NH4)2HPO4	SbPO4	≤ 600
Sb2O5|(NH4)2HPO4	SbPO4	≤ 600
Ca(OH)2|air	CaO	≤ 700
BaCO3|Fe2O3	BaFeO3	≤ 800

BaO2|Fe2O3	BaFeO3	≤ 800
Ag2CO3|V2O5	AgV4O11	≤ 500
Cr2O3|BaO	BaCrO4	≤ 700
Cr2O3|Ba(OH)2	BaCrO4	≤ 700
Cr2O3|BaCO3	BaCrO4	≤ 700
Cr2O3|BaO2	BaCrO4	≤ 700
Cr2O3|Gd2O3	GdCrO3	$700 < T \leq 800$
CaO|Gd2O3	CaGd3O6	≤ 1100
CaCO3|Gd2O3	CaGd3O6	≤ 1100
Ca(OH)2|Gd2O3	CaGd3O6	≤ 1100
K2CO3|Mn2O3	KMn3O6	≤ 900
BaO|SnO2	Ba2SnO4	≤ 800
BaO2|SnO2	Ba2SnO4	≤ 800
BaCO3|SnO2	Ba2SnO4	≤ 800
Ba(OH)2|SnO2	Ba2SnO4	≤ 800
Ba2SnO4|ZrO2	Ba2ZrSnO6	$800 < T \leq 900$
CaO|Cr2O3	CaCrO4	≤ 800
Ca(OH)2|Cr2O3	CaCrO4	≤ 800
TiO2|NiO	No rxn (inert)	≤ 800
Y2O3|Ga2O3	No rxn (inert)	≤ 900
SbO2|Ga2O3	No rxn (inert)	≤ 700
Y2O3|Ga2O3	No rxn (inert)	≤ 700
BaCO3|Mn2O3	No rxn (inert)	≤ 600
Ag|Mn2O3	No rxn (inert)	≤ 500
In2O3|Y2O3	No rxn (inert)	≤ 600
In2O3|Ga2O3	No rxn (inert)	≤ 600
NaVO3|Cr2O3	No rxn (inert)	≤ 900
BaCO3|In2O3	No rxn (inert)	≤ 800

Supplementary Table 3 | Five precursor sets generated for the synthesis of $MgNi(PO_3)_4$ by using a literature-inspired recommendation engine. The similarity between $MgNi(PO_3)_4$ and each reference target is evaluated using the PrecursorSelector encoding model[16] and ranges from -1 to 1. The reference target does not apply to the precursor set with index of 0 because this precursor set is generated based on the most common precursors for Mg, Ni, and P as reported in the literature.

Index	Reference target	Similarity to $MgNi(PO_3)_4$	Precursors for reference target	Recommended precursors for $MgNi(PO_3)_4$
0	N/A	N/A	N/A	MgO, NiO, $NH_4H_2PO_4$
1	$BaMg_2(PO_4)_2$	0.901	$BaCO_3$, MgO, $(NH_4)_2HPO_4$	MgO, NiO, $(NH_4)_2HPO_4$
2	$Ca_8LuMg(PO_4)_7$	0.864	$CaCO_3$, Lu_2O_3, $MgCO_3$, $NH_4H_2PO_4$	$MgCO_3$, NiO, $NH_4H_2PO_4$
3	$Mg_{1.9}Ni_{0.1}TiO_4$	0.830	$MgCO_3$, NiO, TiO_2	$MgCO_3$, NiO, $(NH_4)_2HPO_4$
4	Y_2MoO_6	0.425	$Y_2(C_2O_4)_3$, MoO_3	$MgCO_3$, $Ni(OH)_2$, $(NH_4)_2HPO_4$

Supplementary Table 4 | Compounds that were not synthesized by the A-Lab owing to sluggish kinetics. The driving force listed for each target is calculated either from its precursors or the intermediates that formed during its attempted synthesis. Note that although multiple recipes can be related to one target, we only choose the one recipe for the sake of conciseness.

Target compound	Driving force (meV/atom)	Previous recipe	Strategy	Result
CuAg2P2O7	43	CuO, Ag2CO3, (NH4)2HPO4 @ 900 °C	Redo at 1000 °C (+100 °C)	No obvious change
Mg3NiO4	11	MgO, NiO @ 1000 °C	Regrind and reheat for 4 h	**Pure phase**
Ca2Sn3O8	18	CaO, SnO2 @ 1000 °C	Regrind and reheat for 4 h	No obvious change
V3AgO8	33	Ag2CO3, V2O5 @ 800 °C	Redo at 1000 °C (+200 °C)	No obvious change
Ca2Ti3O8	7	CaO, TiO2 @ 1100 °C	Regrind and reheat for 1 day	No obvious change
KMg3V3CuO12	7	CuO, K2CO3, MgCO3, V2O5 @ 900 °C	Redo at 1100 °C (+200 °C)	No obvious change
Na3V3Cr2O12	8	Cr2O3, Na2CO3, V2O5 @ 800 °C	Redo at 1000 °C (+200 °C)	No obvious change
Y3Ga3In2O12	12	Y2(CO3)3·3H2O, Ga2O3, In2O3 @ 1000 °C	Redo at 1100 °C (+100 °C)	**Pure phase**
CaTiNiP2O9	35	CaO, NH4H2PO4, NiO, TiO2 @ 1100 °C	Regrind and reheat for 1 day	No obvious change

Ca3Ti3Cr2O12	48	CaO, Cr2O3, TiO2 @ 1100 ℃	Regrind and reheat for 4 h	No obvious change
Ba4InSbO8	50	BaCO3, In2O3, Sb2O3 @ 1000 ℃	Redo at 1100 ℃ (+100 ℃)	No target detected

Supplementary Table 5 | Target compounds that were discovered in previous reports after being tested by the A-Lab. For each target, we provide the relevant citations as well as notes regarding its presence in those reports.

Target	Notes	Citations
$CaMn(PO_3)_4$	Reported as "$CaMnP_4O_{12}$" solid solution with same symmetry and similar XRD pattern.	Boonchom, B. & Danvirutai, C. Rapid synthesis, kinetics and thermodynamics of binary Mn0.5Ca0.5(H2PO4)2 · H2O. Journal of Thermal Analysis and Calorimetry vol. 98 717–723 (2009).
$Na_3Ca_{18}Fe(PO_4)_{14}$		Strunenkova, T.V., Morozov, V.A., Khasanov, S., Pokholok, K.V., Redox reactions in ternary Ca, Na, Fe phosphates. I. Crystal structure of o-Ca18Na3Fe(PO4)14. Kristallografiya vol. 42 64-76 (1997)
$Zn_3Ni_4(SbO_6)_2$	Reported as "Ni-doped $Zn_7Sb_2O_{12}$"	Harrington, R., Miles, G. C. & West, A. R. Crystallography of Ni-doped Zn7Sb2O12 and phase equilibria in the system ZnOSb2O5NiO. Journal of the European Ceramic Society vol. 26 2307–2311 (2006).
$MgTi_4(PO_4)_6$	Similar polymorph, space group 167 (previously reported) and 148 (this study).	Zhao, J. & Liu, S. Scalable synthesis of millimeter-sized porous MgTi4(PO4)6 photocatalyst via glass-ceramic combined acid-leaching route. Journal of Non-Crystalline Solids vol. 596 121870 (2022).
Ba_2ZrSnO_6	Reported as solid solution of $BaZrO_3$ - $BaSnO_3$	Hinatsu, Y. Electron Paramagnetic Resonance Spectra of Pr4+in BaCeO3, BaZrO3, BaSnO3, and Their Solid Solutions. Journal of Solid State Chemistry vol. 122 384–389 (1996).
Ba_4InSbO_8	Reported as one of a few compounds in $A_2In_{0.5}Sb_{0.5}O_4$ (A = Sr, Ba)	Heap, R., Islam, M. S. & Slater, P. R. Synthesis and structural characterisation of the new K2NiF4-type phases, A2In0.5Sb0.5O4(A = Sr, Ba). Dalton Transactions 460 (2005)

References

1. M. Geiger and T. Smidt, e3nn: Euclidean Neural Networks. Preprint available at arXiv:2207.09453 (2022).
2. S.-Y. Louis *et al.*, Graph convolutional neural networks with global attention for improved materials property prediction, *Phys. Chem. Chem. Phys.* **22**, 18141-18148 (2020).
3. K. Das *et al.*, CrysXPP: An explainable property predictor for crystalline materials, *npj Comp. Mater.* **8**, 43 (2022).
4. D. Jah *et al.*, ElemNet: Deep Learning the Chemistry of Materials From Only Elemental Composition, Sci. Rep. **8**, 17593 (2018).
5. B. H. Toby and R. B. Von Dreele, GSAS-II: the genesis of a modern open-source all purpose crystallography software package, *J. Appl. Cryst.* **46**, 544-549 (2013).
6. J. Schulman *et al.*, Proximal Policy Optimization Algorithms, Preprint available at arXiv:1707.06347 (2017).
7. E. Liang *et al.*, RLlib: Abstractions for Distributed Reinforcement Learning, Preprint available at arXiv:1712.09381 (2017).
8. N. J. Szymanski *et al.*, Autonomous decision making for solid-state synthesis of inorganic materials. Preprint available at arXiv:2304.09353 (2023).
9. A. R. West, Solid state chemistry and its applications. John Wiley & Sons (2022).
10. S. Sivasangar *et al.*, Screening of modified CaO-based catalysts with a series of dopants for the supercritical water gasification of empty palm fruit bunches to produce hydrogen., *RSC Adv.* **5**, 36798-36808 (2015).
11. A. Sobhani-Nasab *et al.*, Synergetic effect of graphene oxide and C3N4 as co-catalyst for enhanced photocatalytic performance of dyes on Yb2(MoO4)3/YbMoO4 nanocomposite, *Ceram. Int.* **45**, 17847-17858 (2019).
12. D. P. Shoemaker et al. In situ studies of a platform for metastable inorganic crystal growth and materials discovery. *Proc. Natl. Acad. Sci. U.S.A.* 111, 30 (2014).
13. A. J. Martinolich, J. A. Kurzman, & J. R. Neilson. Circumventing Diffusion in Kinetically Controlled Solid-State Metathesis Reactions. *J. Am. Chem.* Soc. 138, 11031–11037 (2016).
14. M. Bianchini et al. The interplay between thermodynamics and kinetics in the solid-state synthesis of layered oxides. *Nat. Mater.* 19, 1088–1095 (2020).
15. A. Miura et al. Observing and Modeling the Sequential Pairwise Reactions that Drive Solid-State Ceramic Synthesis. *Adv. Mater.* 33, 2100312 (2021).
16. T. He *et al.*, Precursor recommendation for inorganic synthesis by machine learning materials similarity from scientific literature. *Sci. Adv.* **9**, eadg8180 (2023).
17. H. Huo et al. Machine-Learning Rationalization and Prediction of Solid-State Synthesis Conditions. *Chem. Mater.* 34, 7323–7336 (2022).

Supplementary Video 1: https://www.youtube.com/watch?v=-XUUI-ThU1Y&feature=youtu.be
Robot arm R1 (Mitsubishi) handling powders and slurries in the sample preparation station used to dispense and mix precursors prior to heating. The video is played at 20-times speed.

Supplementary Video 2: https://youtu.be/26K5r68fzwQ
Robot arm R2 (UR5e) moving crucibles from the sample preparation station to the box furnaces. The video is played at 20-times speed.

Supplementary Video 3: https://www.youtube.com/watch?v=UlLAUEkd06w
Robot arm R3 (UR5e) retrieving powder samples (post-annealing) and cooperating with an Aeris X-ray diffractometer for their characterization. The video is played at 12-times speed.
