# Supplementary material for: Author Correction: An autonomous laboratory for the accelerated synthesis of inorganic materials
Source: Nature. 2026 Jan 19;650(8100):E1. doi: 10.1038/s41586-025-09992-y (PMC12872444; doi:10.1038/s41586-025-09992-y)
Supplement: Supplementary file 3 — Edits to Supplementary Data [file 41586_2025_9992_MOESM3_ESM.pdf]

Update to Supplementary Data of
"*An autonomous laboratory for the accelerated synthesis of inorganic materials*"

06/19/2025

1. Removed the line of $Zn_2Cr_3FeO_8$ from "*20230502 Synthesis Results with Recipes.csv*"
2. Removed "*Zn2Cr3FeO8_800_240_Cr2O3_Fe2O3_ZnO_recipe238_701a5736-30bf-4010-9337-708b06298a8c.jpg*"
3. Removed "*Zn2Cr3FeO8.cif*" from the structure file.
4. Replaced "*Mg3NiO4_1000_240_MgO_NiO_MPReheatrecipe6_reheat-manual-pellet-mixing_structure_1.jpg*" with "*Mg3NiO4_1000_240_MgO_NiO_MPReheatrecipe6_reheat-manual-pellet-mixing_structure_1_updated.jpg*" to correct the Bragg lines of Mg_3NiO_4 in the plot.
5. Added "*Manual_Refinement_Results*" folder for refinement results that were done manually.
6. Merged "*Refinement_Results*" and "*Structure_Files*" folders and structured them to be similar to "*Manual_Refinement_Results*".
7. Added "*Refinement-Table.xlsx*" for details on lattice parameters analysis.
